# Development of an AmpliSeq^TM^ Panel for Next-Generation Sequencing of a Set of Genetic Predictors of Persisting Pain

**DOI:** 10.3389/fphar.2018.01008

**Published:** 2018-09-19

**Authors:** Dario Kringel, Mari A. Kaunisto, Catharina Lippmann, Eija Kalso, Jörn Lötsch

**Affiliations:** ^1^Institute of Clinical Pharmacology, Goethe-University, Frankfurt, Germany; ^2^Institute for Molecular Medicine Finland, HiLIFE, University of Helsinki, Helsinki, Finland; ^3^Fraunhofer Institute for Molecular Biology and Applied Ecology – Project Group Translational Medicine and Pharmacology, Frankfurt, Germany; ^4^Division of Pain Medicine, Department of Anesthesiology, Intensive Care and Pain Medicine, University of Helsinki and Helsinki University Hospital, Helsinki, Finland

**Keywords:** pain, data science, knowledge discovery, functional genomics, next generation sequencing (NGS)

## Abstract

**Background:** Many gene variants modulate the individual perception of pain and possibly also its persistence. The limited selection of single functional variants is increasingly being replaced by analyses of the full coding and regulatory sequences of pain-relevant genes accessible by means of next generation sequencing (NGS).

**Methods:** An NGS panel was created for a set of 77 human genes selected following different lines of evidence supporting their role in persisting pain. To address the role of these candidate genes, we established a sequencing assay based on a custom AmpliSeq^TM^ panel to assess the exomic sequences in 72 subjects of Caucasian ethnicity. To identify the systems biology of the genes, the biological functions associated with these genes were assessed by means of a computational over-representation analysis.

**Results:** Sequencing generated a median of 2.85 ⋅ 10^6^ reads per run with a mean depth close to 200 reads, mean read length of 205 called bases and an average chip loading of 71%. A total of 3,185 genetic variants were called. A computational functional genomics analysis indicated that the proposed NGS gene panel covers biological processes identified previously as characterizing the functional genomics of persisting pain.

**Conclusion:** Results of the NGS assay suggested that the produced nucleotide sequences are comparable to those earned with the classical Sanger sequencing technique. The assay is applicable for small to large-scale experimental setups to target the accessing of information about any nucleotide within the addressed genes in a study cohort.

## Introduction

Persisting pain has been proposed to result from a gene environment interaction where nerve injuries or inflammatory processes act as triggers while the clinical symptoms develop only in a minority of subjects (Lee and Tracey, 2013). A role of the genetic background in pain is supported by evidence of many variants modulating the individual perception of pain and the development of its persistence (Diatchenko et al., 2005; Lötsch et al., 2009b; Mogil, 2012). Genetic variants have been reported to confer protection against pain such as the rs1799971 variant in the μ-opioid receptor gene (*OPRM1*) (Lötsch et al., 2006), or to increase the risk for persisting pain such as the rs12584920 variant of the 5-hydroxytryptamine receptor 2A gene (*HTR2A*) (Nicholl et al., 2011) or the rs734784 polymorphism in the voltage-gated potassium ion channel modifier, subfamily S member 1, gene (*KCNS1*) (Costigan et al., 2010). Nevertheless, the genetic background of persisting pain is still incompletely understood (Mogil, 2009; Lötsch and Geisslinger, 2010) and under intense discussion.

Until recently, research focused on the role of selected functional genetic variants as protective or risk factors of persisting pain. This has changed with the broader availability of next generation sequencing (NGS) (Metzker, 2010). To make use of these technical advancements, we developed a custom AmpliSeq^TM^ library and sequencing assay for efficient detection of genetic variants possibly associated with persisting pain. We propose an assay of a set of 77 genes supported by evidence of an involvement in pain and its development toward persistence. The set size fully uses the technical specifications of the AmpliSeq^TM^ gene sequencing library technique.

## Materials and Methods

### Selection of Genes Relevant for Persisting Pain

A set of candidate genes with shown or biologically plausible relevance to persisting pain was created by applying a combination of criteria, which provided three different genetic subsets. **Subset 1** was chosen exclusively on the basis of computational functional genomics based on a recently published analysis of persisting pain regarded as displaying systemic features of learning and neuronal plasticity (Mansour et al., 2014). As discussed previously (Ultsch et al., 2016), the view of chronic pain as a dysregulation in biological processes of learning and neuronal plasticity (Alvarado et al., 2013) seems to be captured by the controlled vocabulary (Camon et al., 2004) of the Gene Ontology (GO) knowledge base by the GO terms “learning or memory” (GO:0007611)^1^ and “nervous system development” (GO:0007399)^2^. An intersection of the genes annotated to these GO terms with a set of 539 “pain genes” identified empirically as relevant to pain provided the first subset of 34 genes described in detail previously (Ultsch et al., 2016). Briefly, the intersecting set of so-called “pain genes” consists of a combination of (i) genes listed in the PainGenes database (Lacroix-Fralish et al., 2007)^3^, (ii) genes causally involved in human hereditary diseases associated with extreme pain phenotypes, (iii) genes found to be associated with chronic pain in at least three human studies, and (iv) genes coding for targets of novel analgesics under clinical development (Lötsch et al., 2013).

**Subset 2** consisted of genes that were reported to carry variants modulating the risk or the phenotypic symptoms in at least two different clinical settings of persisting pain. They were obtained using (i) a PubMed database search for the string “(chronic OR persisting OR neuropathic OR back OR inflammatory OR musculoskeletal OR visceral OR widespread OR idiopathic OR fibromyalgia) AND pain AND (polymorphism OR variant) NOT review,” to which genes highlighted in overviews on pain genetics (e.g., Edwards, 2006) were added. The intersection of the queried genes with the set of 539 “pain genes” (see above) provided a subset of 13 genes (**Table 1**).

**Table 1:** Genes included in the proposed NGS panel of persisting pain, combined from three subsets included on different bases.

Gene symbol	NCBI	Gene description	Reference
**Subset #1**			
*ADCY1*	107	Adenylate cyclase 1	Vadakkan et al., 2006
*BDNF*	627	Brain-derived neurotrophic factor	Obata and Noguchi, 2006
*CDK5*	1020	Cyclin-dependent kinase 5	Yang et al., 2014
*CHRNB2*	1141	Cholinergic receptor, nicotinic, beta 2	Dineley et al., 2015
*CNR1*	1268	Cannabinoid receptor 1 (brain)	Smith et al., 1998
*DLG4*	1742	Disks, large homolog 4 (Drosophila)	Florio et al., 2009
*DRD1*	1812	Dopamine receptor D1	Onojjighofia et al., 2014
*DRD2*	1813	Dopamine receptor D2	Onojjighofia et al., 2014
*DRD3*	1814	Dopamine receptor D3	Potvin et al., 2009
*EGR1*	1958	Early growth response 1	Ko et al., 2005
*FOS*	2353	Cellular oncogene FOS	Abbadie et al., 1994
*FYN*	2534	Src family tyrosine kinase	Liu et al., 2014
*GABRA5*	2558	GABA A receptor, alpha 5	Bravo-Hernández et al., 2016
*GALR2*	8811	Galanin receptor 2	Hulse et al., 2012
*GRIN1*	2902	Glutamate receptor, NMDA 1	Petrenko et al., 2003
*GRIN2A*	2903	Glutamate receptor, NMDA 2A	Petrenko et al., 2003
*GRIN2B*	2904	Glutamate receptor, NMDA 2B	Petrenko et al., 2003
*GRM5*	2915	Glutamate receptor, metabotropic 5	Walker et al., 2001
*HRH3*	11255	Histamine receptor H3	Huang et al., 2007
*KIT*	3815	Tyrosine kinase KIT	Sun et al., 2009
*NF1*	4763	Neurofibromin 1	Wolters et al., 2015
*NGF*	4803	Nerve growth factor	Kumar and Mahal, 2012
*NTF4*	4909	Neurotrophin 4	Kumar and Mahal, 2012
*NTRK1*	4914	Neurotrophic tyrosine kinase 1	Kumar and Mahal, 2012
*OXT*	5020	Oxytocin prepropeptide	Goodin et al., 2015
*PLCB1*	23236	Phospholipase C, beta 1	Shi T.-J.S. et al., 2008
*PRKCG*	5582	Protein kinase C, gamma	Sluka and Audette, 2006
*PRNP*	5621	Prion protein	Gadotti and Zamponi, 2011
*PTN*	5764	Pleiotrophin	Gramage and Herradon, 2010
*PTPRZ1*	5803	Protein tyrosine phosphatase Z 1	Ultsch et al., 2016
*RELN*	5649	Reelin	Buchheit et al., 2012
*S100B*	6285	S100 calcium binding protein B	Zanette et al., 2014
*SLC6A4*	6532	Serotonin transporter	Offenbaecher et al., 1999
*TH*	7054	Tyrosine hydroxylase	Bravo et al., 2014
**Subset #2**			
*ADRB2*	154	Adrenoceptor beta 2	Hocking et al., 2010
*COMT*	1312	Catechol-*O*-methyltransferase	Feng et al., 2013
*ESR1*	2099	Extrogen Receptor 1	Ribeiro-Dasilva et al., 2009
*GCH1*	2643	GTP cyclohydrolase 1	Tegeder et al., 2006
*IL1B*	3553	Interleukin 1B	Loncar et al., 2013
*IL4*	3565	Interleukin 4	Sugaya et al., 2002
*IL6*	3569	Interleukin 6	Shoskes et al., 2002
*IL10*	3586	Interleukin 10	Stephens et al., 2014
*P2RX7*	5027	Purinergic Receptor P2X7	Sorge et al., 2012
*SCN9A*	6335	Sodium voltage-gated alpha subunit 9	Reimann et al., 2010
*SOD2*	6648	Superoxide dismutase 2	Schwartz et al., 2009
*TNF*	7124	Tumor necrosis factor	Leung and Cahill, 2010
*TRPV1*	7442	Transient receptor potential cation channel, subfamily V, member 1	Bourinet et al., 2014
**Subset #3**			
*ABHD12*	26090	Abhydrolase domain containing 12	Kim, 2015
*ABHD16A*	7920	Abhydrolase domain containing 16A	Kim, 2015
*ABHD6*	57406	Abhydrolase domain containing 6	Kim, 2015
*CACNG2*	10369	Calcium voltage-gated channel auxiliary subunit gamma 2	Nissenbaum et al., 2010
*CSF1*	1435	Colony stimulating factor 1	Thuault, 2016
*DRD4*	1815	Dopamine receptor D4	Buskila et al., 2004
*FAAH*	2166	Fatty acid amide hydrolase	Jayamanne et al., 2006
*FKBP5*	2289	Fk506 binding protein 5	Fujii et al., 2014
*GDNF*	2668	Glial cell derived neurotrophic factor	Sah et al., 2005
*GFRA1*	2674	GDNF family receptor alpha 1	Yamamoto et al., 2003
*GPR132*	29933	G protein-coupled receptor 132	Hohmann et al., 2017
*HCN2*	610	Hyperpolarization-activated cyclic nucleotide-gated	Tsantoulas et al., 2016
*HLA-DQB1*	3119	Major histocompatibility complex, class II, DQ beta 1	Dominguez et al., 2013
*HLA-DRB1*	3123	Major histocompatibility complex, class II, DR beta 1	Dominguez et al., 2013
*HTR1A*	3350	5-hydroxytryptamine (serotonin) receptor 1A	Lindstedt et al., 2012
*HTR2A*	3356	5-hydroxytryptamine (serotonin) receptor 2A	Nicholl et al., 2011
*IL1R2*	7850	Interleukin 1 receptor type 2	Stephens et al., 2014
*KCNS1*	3787	Potassium voltage-gated channel, modifier subfamily S, member 1	Costigan et al., 2010
*LTB4R*	1241	Leukotriene b4 receptor	Zinn et al., 2017
*LTB4R2*	56413	Leukotriene b4 receptor 2	Zinn et al., 2017
*OPRD1*	4985	Opioid receptor delta 1	Law et al., 2013
*OPRK1*	4986	Opioid receptor kappa 1	Guerrero et al., 2010
*OPRM1*	4988	Opioid receptor mu 1	Lötsch and Geisslinger, 2005
*RET*	5979	RET receptor tyrosine kinase	Snider and McMahon, 1998
*RUNX1*	861	Runt related transcription factor 1	Chen et al., 2006
*TLR4*	7099	Toll like Receptor 4	Hutchinson et al., 2010
*TRPA1*	8989	Transient receptor potential cation channel, subfamily A, member 1	Bourinet et al., 2014
*TRPM8*	79054	Transient receptor potential cation channel, subfamily M, member 8	Bourinet et al., 2014
*TRPV4*	59341	Transient receptor potential cation channel, subfamily V, member 4	Bourinet et al., 2014
*TSPO*	706	Translocator protein	Loggia et al., 2015

*Subset #1 comprises *d* = 34 genes that had resulted from a computational functional genomics analysis (Ultsch et al., 2016) pursuing the hypothesis that persisting pain displays systemic features of learning and neuronal plasticity (Mansour et al., 2014). Hence, from a set of genes identified empirically as relevant to pain and listed in the PainGenes database (http://www.jbldesign.com/jmogil/enter.html, Lacroix-Fralish et al., 2007), those were selected that are annotated to the Gene Ontology (Ashburner et al., 2000) terms “learning or memory” and “nervous system development.” The references are those found to provide evidence for an association with pain, except for PTPRZ1 that was a novel finding in (Ultsch et al., 2016). Subset #2 comprises *d* = 13 genes identified empirically as relevant to pain and listed in the PainGenes database (http://www.jbldesign.com/jmogil/enter.html, Lacroix-Fralish et al., 2007) and reported to carry variants that modulated the risk or the symptomatology in at least two different clinical settings of persisting paint. Subset #3 comprises *d* = 30 genes repeatedly shown during the last several years to play a role in the human genetics of persisting pain or recently reported as novel players.*

Finally, **subset 3** comprised genes that have consistently been included in human pain research projects over the last several years. One of them is the *OPRM1* gene that codes for the human μ-opioid receptor and which has been shown to modulate the time course of persisting cancer pain by delaying the necessity of opioid treatment (Lötsch et al., 2010). However, further genes were added such as the *GDNF* gene coding for the glial cell derived neurotrophic factor, which has been shown to be involved in a glia-dependent mechanism of neuropathic pain (Wang et al., 2014) although no modulating human genetic variants have been reported so far. Following expert counseling within the EU-funded “glial-opioid interface in chronic pain, GLORIA” research consortium (Kringel and Lötsch, 2015)^4^, a subset of 30 genes (**Table 1**) was identified. Thus, the complete set as the union of the three subsets comprised 43 + 13 + 30 = 77 genes that are proposed to be included in an NGS panel of human persisting pain.

### DNA Sample Origin

Due to the costs of assay development (for details, see second paragraph of the Discussion), the Ampliseq^TM^ panel was established in a limited number of *n* = 72 DNA samples. This corresponds to the number of samples used in comparable recent studies for NGS assay establishment and validation (Bruera et al., 2018; De Luca et al., 2018; Mustafa et al., 2018; Shah et al., 2018). To further limit the project costs, the Ampliseq^TM^ panel was established in a subset of samples originating from a clinical cohort of 1,000 women who had undergone breast cancer surgery (Kaunisto et al., 2013; Lötsch et al., 2018). The study followed the Declaration of Helsinki and was approved by the Coordinating Ethics Committee of the Helsinki University Hospital. Each participating subject had provided a written informed consent including genetic studies.

Specifically, for the presently reported method establishment, a subsample of 72 women (age 58.4 ± 8 years, mean ± standard deviation, weight 69.3 ± 11 kg), was drawn from the clinical subgroup not having developed persisting pain during the observation period. This was believed to come closer to a random sample than a mixture of patients with persisting and without persisting pain. This limitation of the sample selection has probably affected which and how many variants were identified. However, it is unlikely to have jeopardized the general applicability of the gene selection heuristics, assay establishment and validation, and of the functional analysis of the selected subset of genes.

### DNA Template Preparation and Amplification

A multiplex PCR amplification strategy for the coding gene sequences was accomplished online (Ion Ampliseq^TM^ Designer)^5^ to amplify the target region specified above (for primer sequences, see **Supplementary Table 1**) with 25 base pair exon padding. After a comparison of several primer design options, the design providing the maximum target sequence coverage was chosen. The ordered 1,953 amplicons covered approximately 97.5% of the target sequence (**Supplementary Table 2**). A total of 10 ng DNA per sample was used for the target enrichment by a multiplex PCR and each DNA pool was amplified with the Ion Ampliseq^TM^ Library Kit in conjunction with the Ion Ampliseq^TM^ “custom Primer Pool”-protocols according to the manufacturer’s procedures (Life Technologies, Darmstadt, Germany).

After each pool had undergone 18 PCR cycles, the PCR primers were removed with FuPa Reagent and the amplicons were ligated to the sequencing adaptors with short stretches of index sequences (barcodes) that enabled sample multiplexing for subsequent steps (Ion Xpress^TM^ Barcode Adapters Kit; Life Technologies). After purification with AMPure XP beads (Beckman Coulter, Krefeld, Germany), the barcoded libraries were quantified with a Qubit^®^ 2.0 Fluorimeter (Life Technologies, Darmstadt, Germany) and normalized for DNA concentration to a final concentration of 20 pmol/l using the Ion Library Equalizer^TM^ Kit (Life Technologies, Darmstadt, Germany). Equalized barcoded libraries from seven to eight samples at a time were pooled. To clonally amplify the library DNA onto the Ion Sphere Particles (ISPs; Life Technologies, Darmstadt, Germany), the library pool was subjected to emulsion PCR by using an Ion PGM HI-Q View Template Kit on an PGM OneTouch system (Life Technologies, Darmstadt, Germany) following the manufacturer’s protocol.

### Sequencing

Enriched ISPs which carried many copies of the same DNA fragment were subjected to sequencing on an Ion 318 Chip to sequence pooled libraries with seven to eight samples. During this process, bases are inferred from light intensity signals, a process commonly referred to as base-calling (Ledergerber and Dessimoz, 2011). The number of combined libraries that can be accommodated in a single sequencing run depends on the size of the chip, the balance of barcoded library concentration, and the coverage required. The high-capacity 318 chip was chosen (instead of the low-capacity 314 or the medium-capacity 316 chip) to obtain a high sequencing depth of coverage for a genomic DNA library with >95% of bases at 30x. Sequencing was performed using the sequencing kit (Ion PGM Hi-Q Sequencing Kit; Life Technologies, Darmstadt, Germany) as per the manufacturer’s instructions with the 200 bp single-end run configuration. This kit contained the most advanced sequencing chemistry available to users of the Ion PGM System (Life Technologies, Darmstadt, Germany).

### Data Analysis

#### Bioinformatics Generation of Sequence Information

The raw data (unmapped BAM-files) from the sequencing runs were processed using Torrent Suite Software (Version 5.2.2, Life Technologies, Darmstadt, Germany) to generate read alignments which were filtered by the software into mapped BAM-files using the reference genomic sequence (hg19) of target genes. Variant calling was performed with the Torrent Variant Caller Plugin using as key parameters: minimum allele frequency = 0.15, minimum quality = 10, minimum coverage = 20 and minimum coverage on either strand = 3.

The annotation of called variants was done using the Ion Reporter Software (Version 4.4; Life Technologies, Darmstadt, Germany) for the VCF files that contained the nucleotide reads and the GenomeBrowse^®^ software (Version 2.0.4, Golden Helix, Bozeman, MT, United States) to map the sequences to the reference sequences GRCh37 hg19 (dated February 2009). The SNP and Variation Suite software (Version 8.4.4; Golden Helix, Bozeman, MT, United States) was used for the analysis of sequence quality, coverage and for variant identification.

Based on the observed allelic frequency, the expected number of homozygous and heterozygous carriers of the respective SNP (single nucleotide polymorphism) was calculated using the Hardy-Weinberg equation. Only variants within the Hardy-Weinberg equilibrium as assessed using Fisher’s exact test (Emigh, 1980) were retained. The SNP and Variation Suite software (Version 8.4.4; Golden Helix, Bozeman, MT, United States) was used for the analysis of sequence quality, coverage and for variant identification.

### Assay Validation

Method validation was accomplished by means of Sanger sequencing (Sanger and Coulson, 1975; Sanger et al., 1977) in an independent external laboratory (Eurofins Genomics, Ebersberg, Germany). As performed previously with different AmpliSeq^TM^ panels (Kringel et al., 2017) and other genotyping assays (Skarke et al., 2004, 2005), four DNA samples have been chosen randomly from an independent cohort of healthy subjects and sequenced with the current NGS panel. For the detected variant type, single nucleotide polymorphisms from five different genomic regions for which clinical associations have been reported (**Table 2**), i.e., rs324420 (*FAAH*), rs333970 (*CSF1*), rs4986790 (*TLR4)*, rs4633 (*COMT*), and rs17151558 (*RELN*) were chosen for external sequencing. Amplification of the respective DNA segments was done using PCR primer pairs (forward, reverse) of (i) 5′-TTTCTTAAAAAGGCCAGCCTCCT-3′ and 5′-AATGACCCAAGATGCAGAGCA-3′ (ii) 5′-GCCTTCAACCCCGGGATGG-3′ and 5′-CTCCGATCCCTGGTGCTCCTC-3′ (iii) 5′-TTTATTGCACAGACTTGCGGGTTC-3′ and 5′-AGCCTTTTGAGAGATTTGAGTTTCA-3′ (iv) 5′-CCTTATCGGCTGGAACGAGTT-3′ and 5′-GTAAGGGCTTTGATGCCTGGT-3′ (v) 5′-GTTATTCCTCTGTAAGCAGCTGCCT-3′ and 5′-TGTTTGTTTTAGATTGTGGTGGGTT-3′. Results of Sanger sequencing were aligned with the genomic sequence and analyzed using Chromas Lite^®^ (Version 2.1.1, Technelysium Pty Ltd, South Brisbane, QLD, Australia) and the GenomeBrowse^®^ (Version 2.0.4, Golden Helix, Bozeman, MT, United States) was used to compare the sequences obtained with NGS or Sanger techniques.

**Table 2:** A list of coding human variants in the 77 putative chronic pain genes, found in the present random sample of 72 subjects of Caucasian ethnicity, for which clinical associations have been reported.

Gene	Variant	dbSNP^#^ accession number	Known clinical association	Reference
**Pain context**
*FAAH*	1:46870761-SNV	rs324420	Effect of endocannabinoid degradation on pain	Cajanus et al., 2016
*FAAH*	1:46870761-SNV	rs324420	Cold and heat pain sensitivity	Kim et al., 2006b
*CSF1*	1:110466338-SNV	rs333970	Rheumatoid arthritis	Solus et al., 2015
*NGF*	1:115829313-SNV	rs6330	Procedural pain	Ersig et al., 2017
*NGF*	1:115829313-SNV	rs6330	Susceptibility to migraine	Coskun et al., 2016
*IL1B*	2:113590966-SNV	rs1143634	Adverse effects in postoperative pain	Somogyi et al., 2016
*IL1B*	2:113590966-SNV	rs1143634	Low back pain	Feng et al., 2016
*SCN9A*	2:167099158-SNV	rs6746030	Pain susceptibility in Parkinson disease	Greenbaum et al., 2012
*SCN9A*	2:167099158-SNV	rs6746030	Congenital insensitivity to pain	Klein et al., 2013
*SCN9A*	2:167099158-SNV	rs6746030	Basal Pain Sensitivity	Duan et al., 2015
*SCN9A*	2:167145122-SNV	rs188798505	Altered pain perception	Reimann et al., 2010
*DRD3*	3:113890815-SNV	rs6280	Acute pain in sickle cell disease	Jhun et al., 2014
*DRD3*	3:113890815-SNV	rs6280	Higher prevalence of migraine	Hu et al., 2014
*ADRB2*	5:148206646-SNV	rs1042717	Musculoskeletal pain	Diatchenko et al., 2006
*ADRB2*	5:148206885-SNV	rs1800888	Migraine	Schurks et al., 2009
*ESR1*	6:152129077-SNV	rs2077647	Migraine	Schürks et al., 2010
*ESR1*	6:152129077-SNV	rs2077647	Musculoskeletal pain	Wise et al., 2009
*OPRM1*	6:154360797-SNV	rs1799971	Pain of various origins	Lötsch et al., 2009c
*SOD2*	6:160113872-SNV	rs4880	Migraine	Palmirotta et al., 2015
*IL6*	7:22771039-SNV	rs13306435	Low back pain	Eskola et al., 2010
*OPRK1*	8:54142157-SNV	rs702764	Neuropathic pain	Garassino et al., 2013
*TLR4*	9:120475302-SNV	rs4986790	Musculoskeletal pain	Gbbbebura et al., 2017
*TH*	11:2188238-SNV	rs6357	Widespread Pain	Jhun et al., 2015
*TH*	11:2190951-SNV	rs6356	Migraine	Corominas et al., 2009
*BDNF*	11:27679916-SNV	rs6265	Widespread Pain	Ersig et al., 2017
*DRD2*	11:113283459-SNV	rs6277	Post-surgical pain	Kim et al., 2006a
*DRD2*	11:113283477-SNV	rs6275	Migraine	Onaya et al., 2013
*P2RX7*	12:121600253-SNV	rs208294	Cold pain sensitivity	Ide et al., 2014
*P2RX7*	12:121605355-SNV	rs7958311	Neuropathic pain	Ursu et al., 2014
*HTR2A*	13:47409034-SNV	rs6314	Migraine susceptibility	Yücel et al., 2016
*TRPV1*	17:3480447-SNV	rs8065080	Neuropathic pain	Doehring et al., 2011
*KCNS1*	20:43723627-SNV	rs734784	Neuropathic pain	Doehring et al., 2011
COMT	22:19950235-SNV	rs4633	Postoperative pain	Khalil et al., 2017
*COMT*	22:19950263-SNV	rs6267	Widespread Pain	Lin et al., 2017
*COMT*	22:19951271-SNV	rs4680	Altered pain perception	Wang et al., 2015
**Other context**				
*CSF1*	1:110466466-SNV	rs1058885	Periodontitis	Chen et al., 2014
*CSF1*	1:110466555-SNV	rs2229165	Carcinogenesis/breast cancer	Savas et al., 2006
*NTRK1*	1:156846233-SNV	rs6334	Nephropathy	Hahn et al., 2011
*NTRK1*	1:156848946-SNV	rs6339	Acute myeloid leukemia	Schweinhardt et al., 2008
*SCN9A*	2:167143050-SNV	rs41268673	Erythromelalgia	Klein et al., 2013
*TRPM8*	2:234854550-SNV	rs11562975	Hyperresponsiveness in bronchial asthma	Naumov et al., 2015
*TRPM8*	2:234905078-SNV	rs11563208	Anthropometric parameters	Potapova et al., 2014
*DRD3*	3:113890789-SNV	rs3732783	Phenotypic traits relevant to anorexia nervosa	Root et al., 2011
*KIT*	4:55593464-SNV	rs3822214	Cancer risk	Pelletier and Weidhaas, 2010
*KIT*	4:55602765-SNV	rs3733542	Glandular odontogenic cyst	Siqueira et al., 2017
*HTR1A*	5:63257483-SNV	rs1799921	Bipolar disorders	Goodyer et al., 2010
*ADRB2*	5:148206646-SNV	rs1042717	Cognitive dysfunction in opioid-treated patients with cancer	Kurita et al., 2016
*DRD1*	5:174868840-SNV	rs155417	Alcohol dependence	Hack et al., 2011
*HLA-DQB1*	6:32629920-SNV	rs41544112	Ulcerative colitis	Achkar et al., 2012
*FKBP5*	6:35544942-SNV	rs34866878	Clinical response in pediatric acute myeloid leukemia	Mitra et al., 2011
*CNR1*	6:88853635-SNV	rs1049353	Bone mineral density	Woo et al., 2015
*CNR1*	6:88853635-SNV	rs1049353	Alcohol dependence	Marcos et al., 2012
*CNR1*	6:88853635-SNV	rs1049353	Nicotine dependence	Chen et al., 2008
*CNR1*	6:88853635-SNV	rs1049353	Obesity	Schleinitz et al., 2010
*CNR1*	6:88853635-SNV	rs1049353	Psychiatric disorders	Hillard et al., 2012
*ESR1*	6:152129077-SNV	rs2077647	Breast cancer susceptibility	Li et al., 2016
*ESR1*	6:152129077-SNV	rs2077647	Prostate cancer development	Jurečeková et al., 2015
*ESR1*	6:152129077-SNV	rs2077647	Osteoporosis	Sonoda et al., 2012
*ESR1*	6:152129308-SNV	rs746432	Mood disorders	Mill et al., 2008
*ESR1*	6:152201875-SNV	rs4986934	Endometrial cancer risk	Wedrén et al., 2008
*OPRM1*	6:154360508-SNV	rs6912029	Irritable bowel syndrome	Camilleri et al., 2014
*OPRM1*	6:154360797-SNV	rs1799971	Schizophrenia	Serý et al., 2010
*OPRM1*	6:154414573-SNV	rs562859	Depressive disorder	Garriock et al., 2010
*OPRM1*	6:154414563-SNV	rs675026	Treatment response for opiate dependence	Al-Eitan et al., 2012
*SOD2*	6:160113872-SNV	rs4880	Development of type 2 diabetes mellitus	Li et al., 2015
*SOD2*	6:160113872-SNV	rs4880	Breast cancer susceptibility	Rodrigues et al., 2014
*SOD2*	6:160113872-SNV	rs4880	Asthma	Yucesoy et al., 2012
*ADCY1*	7:45703971-SNV	rs1042009	Bipolar disorder	Shi J. et al., 2008
*RELN*	7:103124207-SNV	rs1062831	Attention deficit hyperactivity disorder	Kwon et al., 2016
*RELN*	7:103251161-SNV	rs362691	Childhood epilepsy	Dutta et al., 2011
*OPRK1*	8:54142154-SNV	rs16918875	Susceptibility to addiction	Kumar et al., 2012
*TRPV1*	8:72948588-SNV	rs13280644	Perception olfactory stimuli	Schütz et al., 2014
*TLR4*	9:120475602-SNV	rs4986791	Breast cancer susceptibility	Milne et al., 2014
*GRIN1*	9:140051238-SNV	rs6293	Schizophrenia	Georgi et al., 2007
*RET*	10:43610119-SNV	rs1799939	Hirschsprung’s disease	Vaclavikova et al., 2014
*RET*	10:43615094-SNV	rs1800862	Medullary thyroid carcinoma	Ceolin et al., 2012
*GFRA1*	10:117884950-SNV	rs2245020	Age-related macular degeneration	Schmidt et al., 2006
*DRD4*	11:637537-Del	rs587776842	Acousticous neurinoma	Nöthen et al., 1994
*BDNF*	11:27720937-SNV	rs66866077	Irritable bowel syndrome-diarrhea	Camilleri et al., 2014
*DRD2*	11:113283484-SNV	rs1801028	Neurologic disorders	Doehring et al., 2009
*GRIN2B*	12:13717508-SNV	rs1806201	Alzheimer’s disease	Andreoli et al., 2014
*TRPV4*	12:110252547-SNV	rs3742030	Hyponatremia	Tian et al., 2009
*P2RX7*	12:121592689-SNV	rs17525809	Multiple sclerosis	Oyanguren-Desez et al., 2011
*HTR2A*	13:47466622-SNV	rs6305	Susceptibility to substance abuse	Herman and Balogh, 2012
*LTB4R*	14:24785092-SNV	rs34645221	Asthma susceptibility	Tulah et al., 2012
*GABRA5*	15:27182357-SNV	rs140682	Autism-spectrum disorders	Hogart et al., 2007
*GRIN2A*	16:9943666-SNV	rs2229193	Hyperactivity disorder	Kim et al., 2017
*DLG4*	17:7099811-SNV	rs17203281	Schizophrenia	Tsai et al., 2007
*SLC6A4*	17:28530193-SNV	rs6352	Autism-spectrum disorders	Prasad et al., 2009
*NF1*	17:29553485-SNV	rs2285892	Neurofibromatosis	Maertens et al., 2007
*HCN2*	19:607984-SNV	rs3752158	Risk of depression	McIntosh et al., 2012
*PRKCG*	19:54394965-SNV	rs3745396	Osteosarcoma susceptibility	Lu et al., 2015
*PRNP*	20:4680251-SNV	rs1799990	Creutzfeldt-Jakob disease	Mead et al., 2009
*HRH3*	20:60791422-SNV	rs3787430	Risk of chronic heart failure	He et al., 2016
*S100B*	21:48022230-SNV	rs1051169	Schizophrenia	Liu et al., 2005

*The selection is restricted to one or two publications per variant, and it focuses on a pain context corresponding to the main aim of the present NGS gene panel; however, functional variants highlighted in another clinical context are additionally provided in the lower part of the table. ^#^Database of Single Nucleotide Polymorphisms (dbSNP). Bethesda (MD, United States): National Center for Biotechnology Information, National Library of Medicine. Available from: http://www.ncbi.nlm.nih.gov/SNP/ (Sherry et al., 2001).*

## Results

**FIGURE 1:**
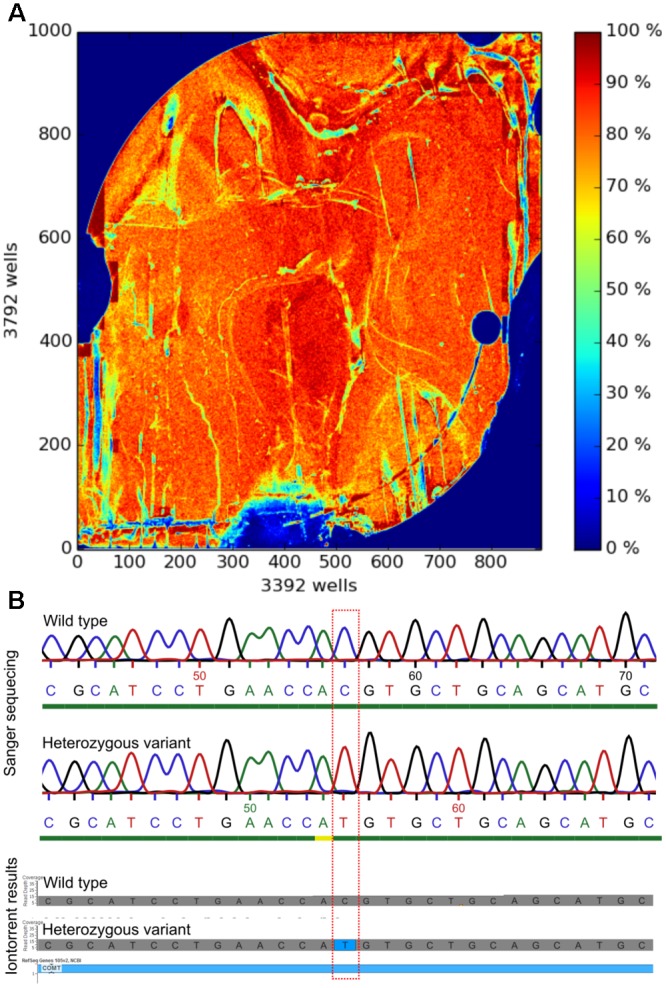
Assay establishment and validation. **(A)** Pseudo-color image of the Ion 318^TM^ v2 Chip plate showing percent loading across the physical surface. This sequencing run had a 76% loading, which ensures a high Ion Sphere Particles (ISP) density. Every 318 chip contains 11 million wells and the color scale on the right side conduces as a loading indicator. Deep red coloration stays for a 100% loading, which means that every well in this area contains an ISP (templated and non-templated) whereas deep blue coloration implies that the wells in this area are empty. **(B)** Alignment of a segment of the ion torrent sequence of the *COMT* gene as a Golden Helix Genome Browse^®^ readout versus the same sequence according to an externally predicted Sanger electropherogram. Highlighted is the *COMT* variant rs4633 (COMT c.186C>T → p.His62 =) as a heterozygous mutation and a non-mutated wild type. The SNP is part of the functional *COMT* haplotype comprising rs4633, rs4818 and rs4680, which showed >11-fold difference in expressed enzyme activity and was reported to be associated with different phenotypes of pain sensitivity (Diatchenko et al., 2005).

The NGS assay of the proposed set of 77 human genes relevant to persisting pain was established in 72 genomic DNA samples. As applied previously (Kringel et al., 2017), only exons including 25 bases of padding around all targeted coding regions for which the realized read-depths for each nucleotide was higher than 20 were contemplated as successfully analyzed. With this acceptance criterion the whole or almost whole coverage of the relevant sequences was obtained (**Table 1**; for details on missing variants, see **Supplementary Table 3**). The NGS sequencing process of the whole patient cohort required ten separate runs, each with samples of *n* = 7 or *n* = 8 patients. Coverage statistics were analogous between all runs and matched the scope of accepted quality levels [20–22]. A median of 2.85 ⋅ 10^6^ reads per run was produced. The mean depth was close to 200 reads, the mean read length of called bases resulted in 205 bases and average chip loading was 71% (**Figure 1A**). To establish a sequencing output with a high density of ISPs on a sequencing chip, the chip loading value should exceed 60% (Life Technologies, Carlsbad, United States). The generated results of all NGS runs matched with the results obtained with Sanger sequencing of random samples (**Figure 1B**), meaning the accordance of nucleotide sequences between NGS and Sanger sequencing was 100% in all validated samples.

**FIGURE 2:**
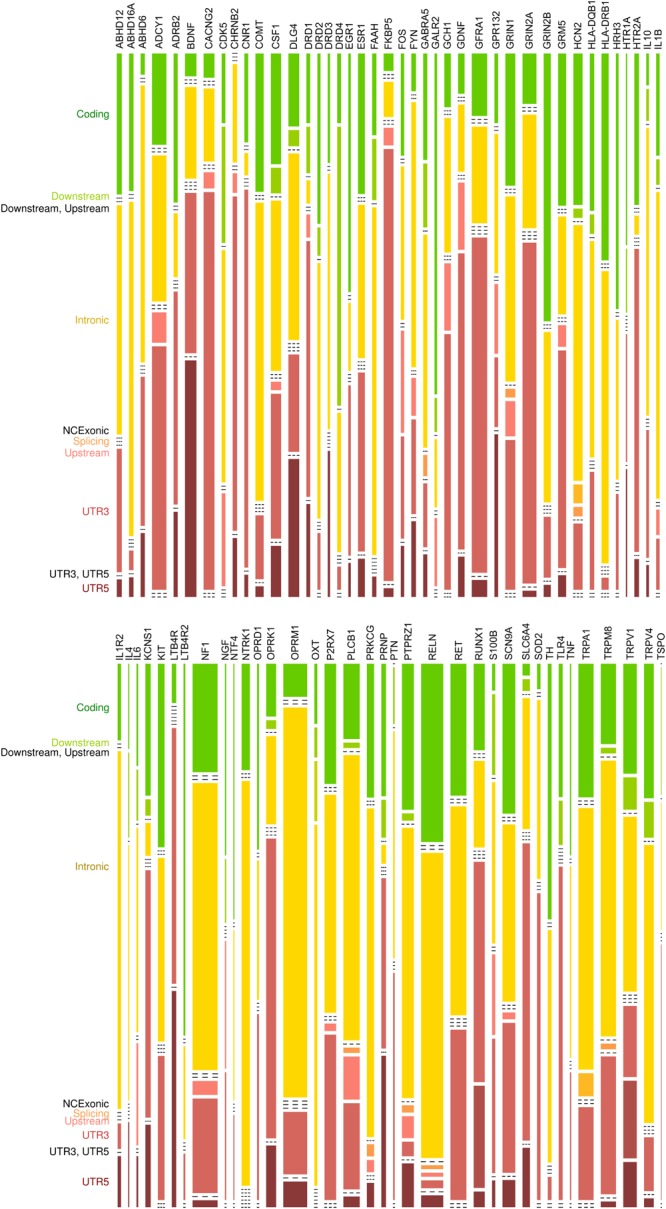
Mosaic plot representing a contingency table of the types of genetic variants detected by means of the present AmpliSeq^TM^ panel versus the genes included in the assay. The vertical size of the cells is proportional to the number of variants of a particular type; the horizontal size of the cells is proportional to the number of variants found in the respective gene. The location of the variants is indicated at the left of the mosaic plot in letters colored similarly to the respective bars in the mosaic plot. Variants were not found at all possible locations of each gene, which causes the reduction of several bars to dashed lines drawn as placeholders and indicating that at the particular location no variant has been found in the respective gene. The figure has been created using the R software package (version 3.4.2 for Linux; http://CRAN.R-project.org/, R Development Core Team, 2008). UTR: untranslated region. NCExonic: Non-coding exonic.

Following elimination of nucleotides agreeing with the standard human genome sequence GRCh37 g1k (dated February 2009), the result of the NGS consisted of a vector of nucleotide information about the *d* = 77 genes for each individual DNA sample (**Figure 2**). This vector had a length equaling the set union of the number of chromosomal positions in which a non-reference nucleotide had been found in any probe of the actual cohort. Specifically, a total of 3,185 genetic variants was found, of which 659 were located in coding parts of the genes, 1,241 were located in introns and 1,285 in the 3′-UTR, 5′-UTR, upstream or downstream regions. The coding variants for which a clinical or phenotypic association have been reported are listed in **Table 2** together with an example of each variant. Most of the observed variants were single nucleotide polymorphisms (*d* = 571) whereas mixed polymorphisms (*d* = 26), nucleotide insertions (*d* = 18) or nucleotide deletions (*d* = 44) were more rarely found.

## Discussion

In this report, development and validation of a novel Ampliseq^TM^ NGS assay for the coding regions and boundary parts of *d* = 77 genes qualifying as candidate modulators of persisting pain is described. The NGS assay produced nucleotide sequences that corresponded, with respect to the selected validation probes, to the results of classical Sanger sequencing. However, the NGS assay substantially reduced the laboratory effort to obtain the genetic information and provides the perquisites to be used in high throughput environments. In particular, the presented NGS assay is convenient for small up to large-scale setups. As mentioned in the methods section, a limitation of the present results applies to the identified genetic variants as only samples from Caucasian women were included. By contrast, the validity of gene selection and assay establishment is unlikely to be reduced by this selection chosen to remain within the financial limits of the present project.

Specifically, as observed previously (Kringel et al., 2017), the comprehensive genetic information and the high throughput are reflected in the assay costs. Specifically, sequencing of the 77 genes in 72 DNA samples required approximately € 18,000 for the AmpliSeq^TM^ custom panel, € 5,500 for library preparation, € 700 for template preparation and € 700 for sequencing. Ten 318 sequencing chips cost around € 7,000 and in addition and basic consumables and laboratory supplies issued approximately € 800. With 7–8 barcoded samples loaded on ten chips, the expense to analyses the gene sequence for a single patient were around € 325. While NGS costs are likely to decrease in the near future (Lohmann and Klein, 2014), present assay establishment was therefore applied in DNA samples planned for future genotype versus phenotype association analysis, which required using DNA from patients of a pain-relevant cohort instead from a true random sample of healthy subjects.

**FIGURE 3:**
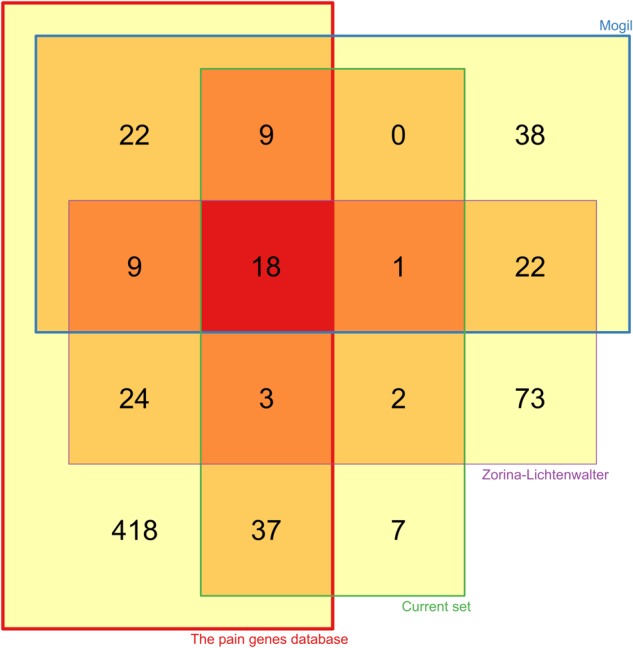
Venn diagram (Venn, 1880) visualizing the intersections between the presently proposed set of human genes involved in modulating the risk or the clinical course of persisting pain (“Current set,” green frame), and two alternative proposals [“Mogil” (Mogil, 2012), blue frame and “Zorina-Lichtenwalter” (Zorina-Lichtenwalter et al., 2016), violet frame]. In addition, a set of *d* = 539 genes identified empirically as relevant to pain and either listed in the PainGenes database (http://www.jbldesign.com/jmogil/enter.html, Lacroix-Fralish et al., 2007) or added because recognized as causing human hereditary diseases associated with extreme pain phenotypes, found to be regulated in chronic pain in at least three studies including human association studies, or being targets of novel analgesics. The number of shared genes between data sets is numerically shown in the respective intersections of the Venn diagram. The figure has been created using the R software package (version 3.4.2 for Linux; http://CRAN.R-project.org/, R Development Core Team, 2008) with the particular package “Vennerable” (Swinton J., https://r-forge.r-project.org/R/?group_id=474).

As a result of the present assay development, a set of *d* = 77 genes was chosen as potentially relevant to persisting pain. The chosen set of genes differs from alternative proposals aiming at similar phenotypes (Mogil, 2012; Zorina-Lichtenwalter et al., 2016). However, when analyzing these alternatives for mutual agreement, only limited overlap could be observed (**Figure 3**). This emphasizes that the genetic architecture of persisting pain is incompletely understood, and several independent lines of research can be pursued. Of note, the present set showed the largest agreement with a set of *d* = 539 genes identified empirically as relevant to pain and listed in the PainGenes database (Lacroix-Fralish et al., 2007)^6^ or recognized as causing human hereditary diseases associated with extreme pain phenotypes (Lötsch et al., 2013; Ultsch et al., 2016). Combining all proposals into a large panel was not an option due to the technical limitations of the IonTorrent restricting the panel size to 500 kb (pipeline version 5.6.2); therefore, further genes would need to be addressed in separate panels.

In the present study sample, selected with a certain bias by using, as explained above for cost saving, clinical samples from only women and only Caucasians, a total of 659 genetic coding variants were found. Regardless of the sample preselection, 105 clinical associations (**Table 2**) could be queried for the observed variants from openly obtainable data sources comprising (i) the Online Mendelian Inheritance in Man (OMIM^®^) database^7^, (ii) the NCBI gene index database^8^, the GeneCards database^9^ [27] and the “1000 Genomes Browser”^10^ (all accessed in December 2017). The observation of functional variants in the present cohort preselected for the absence of pain persistence is plausible as (i) variants can exert protective effects against chronic pain and (ii) most genetic variants identified so far exert only small effects on pain and the individual result of their functional modulations depends on their combined effects or from the sum of positive and negative effects on pain perception (Lötsch et al., 2009a).

The selection of genes (**Table 1**) relied on empirical evidence of their involvement in pain. For subset #1 (*d* = 34), this had been shown for 33 genes in the original paper (Ultsch et al., 2016). As the hypothesis that persisting pain displays systemic features of learning and of neuronal plasticity (Mansour et al., 2014) could be substantiated at a computational functional genomics level, the further gene (*PTPRZ1*, protein tyrosine phosphatase Z 1) can also be regarded as supported by prior knowledge to be included in the present set. The subset comprised, for example, genes associated with the mesolimbic dopaminergic system, i.e., *DRD1, DRD2, DRD3*, which code for dopamine receptors, and *TH*, which is the coding gene for the tyrosine hydroxylase, a metabolic restricting enzyme in dopaminergic pathways, which have been implicated in promoting chronic back pain (Hagelberg et al., 2003, 2004; Jaaskelainen et al., 2014; Martikainen et al., 2015). Further 14 genes were involved in the circadian rhythm recognized as a modulatory factor in various pain conditions such as arthritis (Haus et al., 2012; Gibbs and Ray, 2013) and neuropathic pain (Gilron and Ghasemlou, 2014). The subset further included three NMDA receptor genes (*GRIN1, GRIN2A*, and *GRIN2B*) known to be major players in a number of essential physiological functions including neuroplasticity (Coyle and Tsai, 2004). In addition, metabotropic glutamate receptors (mGluR) have been implemented in several chronic pain conditions. One subtype, mGluR5, coded by *GRM5*, is of particular interest in the context of pain conditions as recent studies showed a pro-nociceptive role of mGluR5 in models of chronic pain (Walker et al., 2001; Crock et al., 2012). Furthermore, genes associated with histaminergic signaling such as *HRH3* have been implicated in pain transmission (Hough and Rice, 2011) and analgesia (Huang et al., 2007).

The second subset of genes relied on a new PubMed search rather than on a previously published and hypothesis-based selection of candidate genes. A computational functional genomics analysis of this subset (details not shown) suggested its involvement in (i) immune processes and (ii) nitric oxide signaling. The genes annotated to the GO term “immune system process” included interleukin (IL1B, IL4, IL6, IL10) (Dinarello, 1994; Choi and Reiser, 1998; Mocellin et al., 2004; Nemeth et al., 2004) and histocompatibility complex related (HLA-B) genes (Dupont and Ceppellini, 1989), which have been shown to be involved in immunological mechanisms of pain (Sato et al., 2002; de Rooij et al., 2009). This is also supported by published evidence for the further genes in this list, such as, *TNF* (Vassalli, 1992; Franchimont et al., 1999), *GCH1* (Schott et al., 1993) and *P2RX7* (Chen and Brosnan, 2006). The second major process group emerging from the functional genomics analysis of the key evidence for genetic modulation of clinical chronic pain was nitric oxide signaling, in particular metabolic processes, summarized in this context under the GO term “reactive oxygen species metabolic process” which includes the genes *IL6* (Deakin et al., 1995), *TNF* (Deakin et al., 1995; Katusic et al., 1998), *ESR1* (Clapauch et al., 2014), *IL10* (Cattaruzza et al., 2003), *GCH1* (Katusic et al., 1998; Zhang et al., 2007), *IL1B* (Katusic et al., 1998), *IL4* (Coccia et al., 2000), *P2RX7* (Gendron et al., 2003), *SOD2* (Fridovich, 1978). Furthermore, catecholamines including noradrenaline, adrenaline and dopamine have multiple functions in the brain and spinal cord including pain perception and processing (D’Mello and Dickenson, 2008). Catechol-*O*-methyltransferase, encoded by the *COMT* gene, is one of several enzymes that degrade dopamine, noradrenaline and adrenaline and has become one of the most frequently addressed genes in pain research (Nackley et al., 2006).

Finally, subset #3 (*d* = 30) consists of genes repeatedly shown to play a role in the genetic modulation of persisting pain in humans or, by contrast, included a few novel items only recently published in the context of pain. This included members of the transient receptor potential (TRP) family (*TRPA1, TRPM8, TRPV4*) that are expressed at nociceptors and which are well established players in the perception of pain via their excitation by chemical, thermal or mechanical stimuli (Clapham, 2003). This similarly applies to the opioidergic system represented by the inclusion of the genes coding for the major opioid receptors (*OPRM1, OPRK1 OPRD1*), which have been associated with variations in pain or opioid response in various settings (Lötsch and Geisslinger, 2005). The most important of this group, the μ-opioid receptor encoded by the *OPRM1* gene, carriers several variants of which the 118 A>G (rs1799971) has been studied most extensively since the early description of its association with a functional phenotype in humans (Lötsch et al., 2002).

**FIGURE 4:**
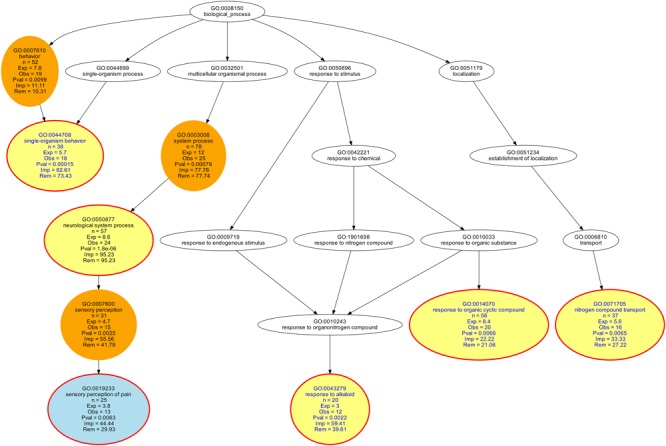
Top–down representation of the annotations (GO terms) representing the taxonomy of the functional differences between the set of *d* = 77 genes included in the proposed NGS panel of persisting pain and two alternative proposals of genes modulating persisting pain in humans (Mogil, 2012; Zorina-Lichtenwalter et al., 2016). The figure represents the results of an over-representation analysis of the present set of *d* = 77 genes against the reference comprising the set intersection of the alternative gene lists. A *p*-value threshold of 0.01 and Bonferroni α-correction were applied. Significant terms are shown as colored circles with the number of member genes, the number of expected genes by change and the significance of the deviation of the observed from the expected number of genes indicated (yellow = headline, red = significant term, blue = significant term located as a leave at the end of a taxonomy in the polyhierarchy). The graphical representation follows the standard of the GO knowledgebase, where GO terms are related to each other by “is-a,” “part-of,” and “regulates” relationships forming a polyhierarchy organized in a directed acyclic graph (DAG, Thulasiraman and Swamy, 1992). The figure has been created using our R library “dbtORA” (https://github.com/IME-TMP-FFM/dbtORA, Lippmann et al., 2018) on the R software package (version 3.4.2 for Linux; http://CRAN.R-project.org/, R Development Core Team, 2008) and the freely available graph visualization software GraphViz (http://www.graphviz.org, Gansner and North, 2000).

Almost half of the present sets of genes were chosen based on a computational functional genomics analysis that attributed persisting pain to GO processes of “learning or memory” and “nervous system development” (Ultsch et al., 2016) as likely to reflect systemic features of persisting pain. This implied a functional bias and therefore, the present set of *d* = 77 genes (**Figure 4**) was analyzed whether this bias prevailed when comparing it with the alternative sets of human genes proposed to modulate persisting pain (Mogil, 2012; Zorina-Lichtenwalter et al., 2016). As applied previously (Lippmann et al., 2018), the biological roles of the set of *d* = 77 genes were queried from the Gene Ontology knowledgebase (GO)^11^ (Ashburner et al., 2000) where the knowledge about the biological processes, the molecular functions and the cellular components of genes is formulated using a controlled and clearly defined vocabulary of GO terms. Particular biological roles of the set of *d* = 77 genes, among all human genes, were analyzed by means of over-representation analysis (ORA). This compared the occurrence of the particular GO terms associated with the present set of genes with their expected occurrence by chance (Backes et al., 2007). In contrast to enrichment analysis, any quantitative criteria such as gene expression values are disregarded (Backes et al., 2007). The analyses were performed using our R library “dbtORA” (Lippmann et al., 2018)^12^ on the R software environment (version 3.4.2 for Linux; R Development Core Team, 2008)^13^.

Surprisingly, the results of this analysis indicated that the functional bias of the present gene set toward “learning or memory” (GO:0007611) and “nervous system development” (GO:0007399) was not maintained against the alternative gene sets. Instead, a few more general GO terms such as “behavior” (“single organism behavior,” GO:0044708), or “response to organic cyclic compound” (GO:0014070) and response to alkaloid (GO:0043279), which could be identified as morphine and cocaine when repeating the analysis with a less conservative α-correction (further details not shown), were overrepresented, as well as the pain specific term “sensory perception of pain” (GO:0019233). A possible explanation that the selection bias of the present gene set was not maintained when comparing it with alternative proposals is that the two biological processes, “learning or memory” and “nervous system development,” reflect indeed an important biological function of persisting pain and even when choosing candidate genes without having these processes in mind as for the alternative gene sets, they are nevertheless included. This may be regarded as support for the present gene set as suitable candidates for future association studies with persisting pain phenotypes.

Although the present gene set has been assembled with a focus of a relevance to pain, many of its members have pharmacological implications. Specifically, 58 of the 77 genes (75%) have been chosen as targets of analgesics, approved or under current clinical development (**Table 3**). Moreover, several of the genes in the present NGS panel have been implicated in pharmacogenetic modulations of drug effects (**Table 4**). Possibly the most widely studied gene in analgesic research is *OPRM1* because coding for the primary target of opioids (Peiro et al., 2016). Several polymorphisms have been described in *OPRM1*, among which the best characterized may be rs1799971 (*OPRM1* 118A>G) that leads to an asparagine to aspartate substitution at the extracellular terminal of the receptor protein (Bond et al., 1998). May studies have addressed this variant (for reviews, see Walter et al., 2013; Somogyi et al., 2015). Summarizing its effects, the variant is associated with decreased receptor expression and signaling efficiency (Oertel et al., 2012) which leads to reproducibly reduced pharmacodynamic effects in human experimental settings while the effect size seems insufficient to be a major factor of opioid response in clinical settings, despite several reports of modulations of opioid demands or side effects. For example, subjects carrying the 118A>G variant were found to have a reduced response to morphine treatment (Hwang et al., 2014), reduced analgesic response to alfentanil (Oertel et al., 2006) and demanded higher doses of morphine for pain relief (Klepstad et al., 2004; Hwang et al., 2014). However, the importance of this variant seems to be comparatively high in patients with an Asian ethnic background, which might be related to the higher allelic frequency as compared to other ethnicities. *COMT* is a key modulator of dopaminergic neurotransmission and in the signaling response to opioids The Val158Met polymorphism (rs4680) causes an amino acid substitution in the enzyme, which reduced the enzyme active to a forth (Peiro et al., 2016). Carriers of the homozygous Met/Met variant had lower morphine requirements than those with a the wild type *COMT* (Rakvag et al., 2005). Furthermore, a modulation of the effects of *TRPV1* targeting analgesics is supported by observations that intronic *TRPV1* variants were associated with insensitivity to capsaicin (Park et al., 2007) while the coding *TRPV1* variant rs8065080 was associated with altered responses to experimentally induced pain(Kim et al., 2004). Moreover, gain-of-function mutations in *TRPV1* have been associated with increased pain sensitivity (Boukalova et al., 2014), for which *TRPV1* antagonists would enable a specific pharmacogenetics-based personalized cure.

**Table 3:** Current targeting of the genes included in the proposed NGS panel of persisting pain by novel drugs that are currently under active clinical development and include analgesia as the main clinical target or at least as one of the intended clinical indication.

Gene	Status	Drug	Action	Company
*ABHD12*	–	–	–	–
*ABHD16A*	–	–	–	–
*ABHD6*	Preclinical	Benzylpiperidin methanone	Acylamino-Acid-Releasing Enzyme	Scripps Research Institute
*ADCY1*	Under Active Development	NB-001	Adenylate Cyclase Inhibitors	Forever Cheer International
*ADRB2*	Phase II/III	Gencaro	Signal Transduction Modulators	ARCA
*BDNF*	Phase I	CXB-909	Nerve Growth Factor (NGF) Enhancers	Krenitsky
*CACNG2*	Preclinical	Hanfangchin	Calcium Channel Blockers	Millenia Hope Kaken
*CDK5*	Biological Testing	Litvinolin	CDK5/p25 Inhibitors	Hong Kong University
*CHRNB2*	Biological Testing	Epiboxidine	Nicotinic alpha4beta2 Receptor Agonists	Pfizer
*CNR1*	Registered	Epidiolex	Cannabinoid Receptor Agonists	InSys Therapeutics
*COMT*	Clinical	Nitecapone	Catechol-*O*-Methyl Transferase (COMT) Inhibitors	Orion
*CSF1*	–	–	–	–
*DLG4*	Preclinical	AB-125	Protein Inhibitors	Lundbeck University of Copenhagen
*DRD1*	Phase II/III	Ecopipam	Dopamine D1 Receptor (DRD1) Antagonists	Merck & Co.
*DRD2*	Phase II/III	Sarizotan hydrochloride	Dopamine D2 Receptor (DRD2) Antagonists	Newron
*DRD3*	Phase II	Brilaroxazine	D3 Receptor (DRD3) Agonists	Reviva Pharmaceuticals
*DRD4*	Biological Testing	Mesulergine hydrochloride	Dopamine Receptor Agonists	Novartis
*EGR1*	Phase II	Brivoligide	EGR1 Expression Inhibitors	Adynxx
*ESR1*	Phase II	Zindoxifene	Selective Estrogen Receptor Modulators	Evonik
*FAAH*	Phase I/II	Minerval	Fatty Acid Amide Hydrolase (FAAH) Inhibitors	Scripps Research Institute
*FKBP5*	Phase II	Barusiban	Oxytocin Receptor Antagonist	Ferring
*FOS*	Registered	Macrilen	FOS Expression Enhancers	Strongbridge Biopharma
*FYN*	Phase II	Bafetinib	Fyn Kinase Inhibitors	Nippon Shinyaku
*GABRA5*	Phase III	Ganaxolone	GABA(A) Receptor Modulators	Marinus Pharmaceuticals
*GALR2*	Preclinical	NAX-810-2	GAL2 Receptor Ligands	NeuroAdjuvants
*GCH1*	–	–	–	–
*GDNF*	Phase II	Edonerpic maleate	Signal Transduction Modulators	Toyama
*GFRA1*	–	–	–	–
*GPR132*	–	–	–	–
*GRIN1*	Phase II	Dimiracetam	Signal Transduction Modulators	Metys Pharmaceuticals
*GRIN2A*	Phase I	Dexanabinol	NMDA Receptor Antagonists	e-Therapeutics Pharmos
*GRIN2B*	Phase I	Gacyclidine	NMDA Receptor Antagonists	INSERM
*GRM5*	Phase II	Mavoglurant	Signal Transduction Modulators	Novartis
*HCN2*	Clinical	Ivabradine	Adrenoceptor Antagonists	Servier
*HLA-DQB1*	–	–	–	–
*HLA-DRB1*	–	–	–	–
*HRH3*	Phase I	Immethridine	Histalean	Abbott
*HTR1A*	Phase II	Eltoprazine hydrochloride	5-HT1A Receptor Agonists	Elto Pharma
*HTR2A*	Phase II	Midomafetamine	5-HT2 Receptor Agonists	Assoc
*IL10*	Phase II	BT-063	Signal Transduction Modulators Anti-IL-10	Biotest AG
*IL1B*	Phase III	Resunab	IL-1beta Inhibitors	Corbus
*IL1R2*	–	–	–	–
*IL4*	–	–	–	–
*IL6*	Preclinical	Azintrel	Signal Transduction Modulators Anti-IL-6	Jazz Pharmaceuticals
*KCNS1*	Preclinical	Crotamine	Voltage-Gated K(V) Channel Blockers	Celtic Biotech
*KIT*	Phase II	Vatalanib succinate	KIT (C-KIT) Inhibitors	Novartis
*LTB4R*	Phase II	Coversin	Signal Transduction Modulators	Akari Therapeutics
*LTB4R2*	Phase II	Coversin	Signal Transduction Modulators	Akari Therapeutics
*NF1*	–	–	–	–
*NGF*	Phase III	Tanezumab	Anti-Nerve Growth Factor (NGF)	Pfizer
*NTF4*	–	–	–	–
*NTRK1*	Phase II	Danusertib	NTRK1 Inhibitors	Pfizer
*OPRD1*	Preclinical	Metenkephalin	Delta-Opioid Receptor Agonists	TNI Pharmaceuticals
*OPRK1*	Phase III	Morphine glucuronide	Opioid Receptor Agonists	PAION
*OPRM1*	Registered	Naltrexone	mu-Opioid Receptor Antagonists	Pfizer
*OXT*	Phase II	Barusiban	Oxytocin Receptor Antagonist	Ferring
*P2RX7*	Preclinical	BIL-06v	Anti-P2RX7	Biosceptre International
*PLCB1*	Biological Testing	Vinaxanthone	Signal Transduction Modulators	Roche
*PRKCG*	Phase III	Rydapt	Protein Kinase C (PKC) Inhibitors	Yeda
*PRNP*	–	–	–	–
*PTN*	–	–	–	–
*PTPRZ1*	–	–	–	–
*RELN*	Preclinical	IAIPs	Serine Protease Inhibitors	ProThera Biologics
*RET*	Phase II	Danusertib	Ret (RET) Inhibitors	Pfizer
*RUNX1*	–	–	–	–
*S100B*	–	–	–	–
*SCN9A*	Phase III	Priralfinamide	Voltage-Gated Sodium Channel Blockers	Newron
*SLC6A4*	Phase II	Litoxetine	Signal Transduction Modulators	Sanofi
*SOD2*	Phase II	Avasopasem manganese	Superoxide Dismutase (SOD) Mimetics	MetaPhore
*TH*	–	–	–	–
*TLR4*	Phase II	Eritoran tetrasodium	Toll-Like Receptor 4 (TLR4) Antagonists	Eisai
*TNF*	Phase III	Givinostat hydrochloride	TNF-alpha Release Inhibitors	Italfarmaco
*TRPA1*	Phase II	Cannabidivarin	TRPA1 Agonists	GW Pharmaceuticals
*TRPM8*	Phase II	Cannabidivarin	TRPM8 Antagonists	GW Pharmaceuticals
*TRPV1*	Phase I/II	Resiniferatoxin	TRPV1 (Vanilloid VR1 Receptor) Agonists	Icos
*TRPV4*	Phase II	GSK-2798745	TRPV4 Antagonists	GlaxoSmithKline
*TSPO*	Clinical	[11C]CB-184	Translocator Protein (TSPO) Ligands	Tokyo Metrop Geriatr Hosp Inst Gerontol

*The information was queried from the Thomson Reuters Integrity database at https://integrity.thomson-pharma.com on July 11, 2018.*

**Table 4:** Summary of variants in genes included in the proposed NGS panel of persisting pain, that have been implicated in a pharmacogenetic context to modulate the effects of drugs administered for the treatment of pain or as disease modifying therapeutics in painful disease.

Modulated process	Gene	Variant	Affected drug	Findings	Reference
					
G protein coupled signaling	*COMT*	rs4680 (Val158Met)	Morphine	Carriers of val/val and val/met genotype required higher morphine dose compared to carriers of met/met genotype	Reyes-Gibby et al., 2007
	*DRD2*	rs6275	Heroine	Polymorphism is associated with decreased likelihood of headache disorders	Cargnin et al., 2014
	*DRD4*	rs1800955	Heroine	Polymorphism had lower pain threshold versus CC/CT controls	Ho et al., 2008
	*OPRM1*	rs1799971 (A118G)	Various opioids	Tendency toward increased pain in dose-dependent manner with the μ-opioid receptor variant 118G	Lötsch et al., 2009c
	*OPRK1*	rs1051660	Morphine	Patients with the polymorphism and cancer-related pain may require a reduced dose escalation of morphine	Chatti et al., 2017
Neurotransmitters	*BDNF*	rs6265	Various opioids	Polymorphism is associated with decreased likelihood of headache disorders	Cargnin et al., 2014
	*HTR2A*	rs12584920	Various opioids	Increased likelihood of having chronic widespread pain	Nicholl et al., 2011
Ion Channels	*TRPV1*	7 intronic SNPs	Capsaicin	TRPV1 polymorphisms had only 50% of the mRNA and protein expression levels of normally sensing subjects	Park et al., 2007
Proinflammatory Cytokines	*IL6*	rs1800795	Etanercept	Polymorphism is associated with increased response to adalimumab, etanercept or infliximab in people with painful Arthritis	Davila-Fajardo et al., 2014
Other	*ESR1*	rs2234693	Leflunomide	Polymorphism is associated with increased response to leflunomide in women with painful Arthritis	Dziedziejko et al., 2011
	*FAAH*	rs2295632	Various opioids	Polymorphism is associated with increased risk of Respiratory Insufficiency	Biesiada et al., 2014
	*TLR4*	rs4986790	Methotrexate	Polymorphism associated with increased risk of adverse drug events when treated with folic acid and methotrexate in people with Arthritis	Kooloos et al., 2010
	*TNF*	rs361525	Infliximab	Polymorphism is associated with increased response to infliximab in people with painful Arthritis	Maxwell et al., 2008

*The information was derived by literature search and by querying the Pharmacogenetics Research Network/Knowledge base at http://www.pharmgkb.org (accessed in July 2018). Only key or example references are given.*

## Conclusion

The breakthrough in mapping the whole human genome (Lander et al., 2001; Venter et al., 2001) along with genome wide association studies (GWAS) has led to rapid advances in the knowledge of the genetic bases of human diseases (Wellcome Trust Case Control and Consortium, 2007). Genetic research in pain medicine has directed to the recognition of genes in which variants influence pain behavior, post-operative drug requirements, and the temporal developments of pain toward persistence (James, 2013). While many candidate gene association studies have identified multiple genes relevant for pain phenotypes (Fillingim et al., 2008), pain related genetic studies have so far been owned by investigations of a limited number of genes. Roughly ten genes or gene complexes account for over half of the extant findings and several of these candidate gene associations have held up in replication (Mogil, 2012). The selection of variants has been limited and they have been addressed in most studies repeatedly, leading to the perception that genetic research in pain produces often unsatisfactory results (Mogil, 2009). However, this may soon change with the arise of new technologies. In this manuscript, we present a validated NGS assay for a set of 77 genes supported by empirical evidence and computational functional genomics analyses as relevant factors modulating the risk for persisting pain or its clinical picture.

## Author Contributions

JL, DK, and EK conceived and designed the experiments. DK performed the experiments. JL and DK analyzed the data and wrote the paper. CL provided methodological expertise and bioinformatical tools. DK and JL interpreted the results. EK and MK provided DNA samples.

## Conflict of Interest Statement

The authors declare that the research was conducted in the absence of any commercial or financial relationships that could be construed as a potential conflict of interest.

## Footnotes

1 http://amigo.geneontology.org/amigo/term/GO:0007611

2 http://amigo.geneontology.org/amigo/term/GO:0007399

3 http://www.jbldesign.com/jmogil/enter.html

4 http://gloria.helsinki.fi

5 http://www.ampliseq.com

6 http://www.jbldesign.com/jmogil/enter.html

7 http://www.ncbi.nlm.nih.gov/omim

8 http://www.ncbi.nlm.nih.gov/gene

9 http://www.genecards.org

10 https://www.ncbi.nlm.nih.gov/variation/tools/1000genomes

11 http://www.geneontology.org/

12 https://github.com/IME-TMP-FFM/dbtORA

13 http://CRAN.R-project.org/
