# Supplementary material for: Development of an AmpliSeqTM Panel for Next-Generation Sequencing of a Set of Genetic Predictors of Persisting Pain
Source: Front Pharmacol. 2018 Sep 19;9:1008. doi: 10.3389/fphar.2018.01008 (PMC6156278; doi:10.3389/fphar.2018.01008)
Supplement: Supplementary file 1 [file Table_1.DOCX]

Supplementary Table 1: Ampliseq primer sequences used for the amplification of DNA segments of the 77 genes suitable for NGS analysis by means of ion semiconductor sequencing.

| Gene | Primer forward | Primer reverse | Amplicon start | Amplicon stop |
| --- | --- | --- | --- | --- |
| ABHD12 | CACAAACAGCCCAGCAATAGTT | AGGTTCTGCCCTCTTTCATTGG | 25297571 | 25297827 |
|  | ACTGGCTGAGTGTGCAAGATG | TGTTTCCCAAAACACACCCTGAA | 25284132 | 25284369 |
|  | CGGCCACCTCCTCTCTAGT | TGGTTTTCCGTTCACAGAAGCA | 25371571 | 25371831 |
|  | AGCTGCTGGGACTAGAAAATTTCA | GTCCAGGTTCTTTCTGCACCTT | 25280711 | 25281084 |
|  | ACTAATGGTGCCACAGGGTTTG | GGAGGTGTGTTTGTCCAGGTTT | 25289971 | 25290279 |
|  | TTCACCTGGCTGCAGGTTATAC | TGTGGTCTCCTTGTGCTTTCTG | 25288409 | 25288783 |
|  | CTGCGCAAAGTGAGGGACCG | CGCCTGAAGCAGAACCTACG | 25371093 | 25371237 |
|  | GCCTGCTGCCATTAAGTCTCC | GGATCTTTGGTCTGAGCTCAAATGATT | 25281200 | 25281572 |
|  | CTTTCAGGACGTCTTCTCCACT | CTACATTATAATGTCACATCGTTGCAAGTG | 25275032 | 25275394 |
|  | AAAAATGAACAACATTTCAGAGGCCAAT | CTTTCTGAGAGCTTCTGCAGTGT | 25303856 | 25304225 |
|  | AGCGCTCATGCTCCAAGG | CGCGCTCCTTGGAACTAG | 25371297 | 25371602 |
|  | ATGGAACCACTGGAGTCATTCTG | CCCTCAACCTGAGATCTTGTTG | 25281029 | 25281242 |
|  | GCTCCCAAATCACTGTGACTCT | TATGTGGGACTCTGGACTGGTT | 25282723 | 25283096 |
|  | GCTCCATCAGGGTCTTTGTCAG | AGGCAGCATGTCACAGTCTTTA | 25295403 | 25295749 |
|  | CCCAGCTCCTCAGGTGTATCAT | CCAGTGCTAGAGGGATCTCCAT | 25288926 | 25289286 |
|  | CCCTAGCAGGCTTTCCCTTTTT | CTGCTCCCTTAGGTAGGGTAGT | 25287310 | 25287680 |
|  | CAGGCAGTGGTGAAAATGGATTT | CTTCACGAAATGCAGAGTTCACAAT | 25275342 | 25275716 |
|  | CTTCAGAACTCTGCCTTTCCCTT | GTCGCCAGGATGAGGACAGAAG | 25300653 | 25301016 |
|  | ACTGTACACTTAAGATATGTACCCTTCACT | AAAAATATGTGTGGCTCTTGTTTAGGG | 25319722 | 25320093 |
| ABHD16A | GCAAATGGCGAGTGGACTTTTC | CTGTGGAATGAGAAGCTTCCATTC | 31658168 | 31658534 |
|  | CAAGTCTTTTAAGGAAAAGATCATGCCT | CTTCCATCAGGCATCTTCTTCCT | 31668569 | 31668943 |
|  | CATCCACCACCCACTCTACAAA | GGAAGAGGACTGGTGTCTGTCT | 31655158 | 31655492 |
|  | GGACAGGTCAGGGCTGATTTTT | GAACCAAGGATGAGATCATCACCA | 31655695 | 31656053 |
|  | ACTCAGAGATTCTACTTCCTCTCTCTTC | ATCCCTTCCTTCTACACCTTCTCT | 31657230 | 31657604 |
|  | TGGATGTCTGAGGTCTGGAGAA | GGTATTGACCTCCGCTTGGTTT | 31660975 | 31661342 |
|  | ACATTTAGAGTGTGCCTGTGTGT | GGTGAGGAAGAGGAGTTTCTGTG | 31669688 | 31670061 |
|  | GAAGACGCAATGGCCAAGATG | GAGGAAAGTAAAGCTGTGAAGGTCA | 31659467 | 31659827 |
|  | GGACACAGAGATGGGAAGGGTTA | GGAAAGACCCTCTTATTTGTGATTCTCT | 31654594 | 31654940 |
|  | GCATGAAGAGGCACTGAAGAAGAG | CGTGGTACAGTCCAAACCCTTTA | 31670542 | 31670898 |
|  | GCCAGTGGCCTTTTACCAACTT | TGAGATGGCTCCCTTTTCTTGG | 31656390 | 31656729 |
|  | ATGGCTGACCAGAGGGAAACAG | GCTTGGAGTTATTTGCATCTAAGCTG | 31657791 | 31658017 |
|  | CCTCTACTGCCTTCCTCACACT | TGTATCCAGGCTCTGTGTACCT | 31659301 | 31659660 |
|  | ACATAAAGGTCCTCACTATTCACGGA | GCCTATAGGGTCAGTGGGATGA | 31656613 | 31656985 |
|  | TGCCATAAATGATCTACACAAACCATACA | GCCTGCCTTCCAATGCCATTAT | 31660665 | 31661038 |
|  | CCATTCAAGAACCCTTCCCACTT | GGAAGGTGAGAAGGGATCCAGT | 31655235 | 31655605 |
|  | GTTTGGAACAGCCATACCCTAAGA | TACTGAGCACCTACTGTATTCATGGT | 31668898 | 31669272 |
|  | GTTGGACATGATGTCCTCAGGAA | CCTGAGTGAAGGGCTAGGTCAA | 31655856 | 31656219 |
|  | GGATACAGCAATGTGCAAGCAA | CATCCTATGCCCTCAGTTCCTT | 31664576 | 31664949 |
|  | CCTTATTTCCCATTATGGGCACTTCT | TCTTCTCAGGAAGCACTGAAAATGT | 31670834 | 31671208 |
|  | CCCACTTTCCACAAACTATAAACAGC | GAAGTGGCAGCTTTTGTAGAGTG | 31654876 | 31655191 |
|  | GAGAAGAAAGAAAAGACACCTAGACAATCT | GCCGTTGAATTTTGCTGTCAGA | 31670224 | 31670598 |
| ABHD6 | TATATTAACTCTGGGCTGCTGTCAGA | TCAATGAGTAGGTGAAGGAGGGAAT | 58260287 | 58260639 |
|  | TCTGTGGATGGATGGCTTTTATTTCT | AGCGATCAGCTCACTCCATAAATAAC | 58270911 | 58271285 |
|  | GAGTGGCCAAGACCCATACATC | CACTTGTAGACAGAATGTACAGGAGA | 58254919 | 58255291 |
|  | CGGGTGCTAGGAGACAGTTG | CTCCTTAAAGGCGCAGCGT | 58223294 | 58223511 |
|  | GCAGCCACCTTGGACCATAATTAAA | CCCTCCATGCGTGTAAACATTTT | 58279785 | 58280051 |
|  | CTGTGGGCACTCAGTAGTGATG | GAAGACTTCATGGTAGATTTCATGGCT | 58279387 | 58279754 |
|  | TGTCTAAGTAATCAGACTCAGGTTCCA | CCAAAGCCTGTTAAACAAGCCTTATTTTAA | 58280246 | 58280570 |
|  | TGTTCTTTACCCTCACTCTGGGAA | CTTTTCTCTCTGTCTGAAATACTTCCTGAT | 58242179 | 58242553 |
|  | GTGTGCTGCATCATGGGAATTC | CCGCAGAATCAAGGGATAGTCAC | 58270655 | 58271024 |
|  | CAGTAGTCATTTTCAGTTGTCCATAGGA | TGCATATCTCTCAAGCATACATCAAAAAGA | 58256547 | 58256911 |
|  | CCCGTCCCTTATCCCTGGTATC | GTCTCCAACTATAAGGAATGTCTCAAAGAA | 58279615 | 58279850 |
|  | CTGCAAGACCAGGGAGGAAAGTG | AAATAGAAAAGAGGCCCACGAGTT | 58279995 | 58280308 |
|  | GGGTAAAGCTCAGGAGGACTCTA | CAGCACCCAACTCCCTTTTCTTA | 58252753 | 58253121 |
|  | GCCTGTCCCTTTCTCTCTGATG | ACAGTTAGCGTCTCGCACAA | 58222977 | 58223344 |
|  | CGGCTTTTCTGTCGGAGGA | CCGACTCTTTCCACTCGATGC | 58223463 | 58223762 |
|  | CCTGGCACAGAGTTGCAGAATAA | TCGATTATGAGCTTGGCTGTCTTC | 58279103 | 58279443 |
| ADCY1 | CCTTCCTGACCCATCCTTTGAA | TACGAAGAATGTGACATATTGTGAGACAC | 45743170 | 45743442 |
|  | TGGAGGAGGAGCATTGTCCA | TCAGTGGCTCTTGCTGTAACC | 45753715 | 45753973 |
|  | CATCTCCATCATCAGCAACTCCAA | GTGCACTATTTACAAAGCCTCTTCAATAAA | 45756898 | 45757135 |
|  | AAAAAGCCAAGAGACATGTGAGGA | CATGCCTGTTACTGACGTCTCT | 45724424 | 45724793 |
|  | GCAAGGACTCAGATTTCCCTATGATAAG | CGTCAGATGCTTGCTGAAGACT | 45725973 | 45726346 |
|  | AGCAAGGACAAGACCCAGTTTC | CCCAGAACCTGAAGCAAGGATTT | 45697211 | 45697568 |
|  | ACCCAAGTGATTTCAGCAAGAGT | GAAGGCCAGCTCTCCCAAAT | 45753107 | 45753481 |
|  | GAAGGCACTATCCAGAAATAGTGGA | CTCTCTGCTCCTATTCTGAAAAGTCG | 45754802 | 45755100 |
|  | CCCTGGAGTGTAGACCTGTGT | GCCTCTTGCACCATCACAGAA | 45755117 | 45755489 |
|  | GTGATTTTGACCAATGCCTTTCCA | GCAGCCTGGAGATTCCACATAA | 45755519 | 45755836 |
|  | TGCCTGATGCCGTAGAGTAGAA | GGCAAACTTGTATGCATGGCTTAT | 45757273 | 45757559 |
|  | AGGTGTCCGTAGTTGGCAAAAA | CAGATCACCCAAATCCATCTGTGAA | 45758210 | 45758546 |
|  | GGGTTTGTCTGTAAGCTGCAGT | TGTATGAACAAACAGAAGGATAAGGCA | 45758588 | 45758962 |
|  | CACTAGAGGCTGTGCTTAATTCAAATC | TGGACTTCAGTGCTGATGTAGTAGA | 45759193 | 45759524 |
|  | AGCAGGAGTTCAGAGGGCTTAT | TGGGAGGTGAGAAACAAGTTCTG | 45761325 | 45761677 |
|  | GCCTTCCACCTGTAAAAACTGCA | GGAATGGAATGTACCACTACGAGATG | 45761836 | 45762209 |
|  | TCTCACTGGGTAACTTCTCATGATAGAT | GAATGTCTTCAAAACATCCACATAACCATT | 45762436 | 45762772 |
|  | TCTTTACCCTGAAGTACAAACATGTCG | GGACAATAACTGTGGGAGACGAA | 45717622 | 45717994 |
|  | TTTGGTCACCTTCGTGTCCTATG | CGTAGGAGTGACCTTGAACACAC | 45614643 | 45614929 |
|  | TCTTAGTCTCCTACCTGTAGAATGGAATG | CCTGTAAGACCAGGGTACCTCAT | 45747787 | 45748161 |
|  | CTGTGTTTGTCAGGATGGCATTC | GGAATTCTGATCTAGCCAAATGGCA | 45701569 | 45701916 |
|  | CATTTCTGGTCTGCATTGAGGAATAAATG | CCTTGCTGGAAGAAATGCTTAGC | 45662094 | 45662468 |
|  | GTGCGACGAGGAGTTCGCT | CGGACGTTGGTTACCACG | 45614247 | 45614477 |
|  | TCGTCGGTGTGAACATGTATGG | CAGCCTCAAATACCAACATGTACCA | 45632380 | 45632647 |
|  | TTCCAATTGATGAGCCTCAGTACTG | TGGAATGATTTGTGCACTGATGGT | 45757807 | 45758175 |
|  | TTGCTAAATTAAAGCAATGCAATTCCTCTT | CCCATTTGATGTCAGAGAGGCTT | 45760853 | 45761091 |
|  | TCAGCACTCTCTCCTGATCGTA | TTTTTGGCAAGTATTTAATAATGGAAGGAAATG | 45704077 | 45704356 |
|  | TCCGCGACCCTAGAGCTT | GCGTGACCTCGAGTACCTT | 45613436 | 45613803 |
|  | CTCTCACTGCATGGATAGTGGT | CCGCTCATATTCAACAGAAGTGGA | 45754219 | 45754550 |
|  | TCTGTCTATCCCAGCCGGATTTA | CCCACCCAGATAAACCAAGTTCC | 45756030 | 45756399 |
|  | CCTGGAAAGATTCGAAGTGTCACA | CTTCCTCTCACTGCCCTACAAG | 45756439 | 45756803 |
|  | TGCTTCACAATGGACTGGGAAAA | GTGGTCGATTTTCTGATTTCTTTCTTTCTT | 45759782 | 45760156 |
|  | GTCTTTTGCCGTGACCACAAAT | GGCACTAAACATAATGGAAAACACAAGG | 45760356 | 45760635 |
|  | GCCTGTCCTTAGGGTGTGGAT | GGGAGCTCCCTGTCATAACCA | 45755437 | 45755574 |
|  | GTTAGAGCCATTCGGGTAGGATTC | CTTGCAAGCAACTGGAAGCATT | 45757071 | 45757327 |
|  | GGGTCTTATTCGAGGAACTCACA | GAAAGGTTGAAGCAACTCCATTTCC | 45758118 | 45758267 |
|  | GGAGGGAGAGTACAGGTGCTTT | CTGTGTAAGAGAGAAGTCAATCCATCAG | 45717392 | 45717764 |
|  | GTGGATGTAGACCATCAGCACA | CCTGGGAGTAGGACTGGTAGTAGA | 45742855 | 45743226 |
|  | GAACTTGTCTGCCCAAAGCAAA | GCACTCAAGCTGCTCCTGTAAT | 45743974 | 45744348 |
|  | GTCTTCACCTGCCCTTTGCTAA | GATTCAGTAGCACCGCTGCTTA | 45725514 | 45725866 |
|  | ATCCAGGTTAACGATGCTGCTT | CTGAACAAGGTGATATGCAGGTATAGG | 45719175 | 45719530 |
|  | CCTGGGCTTGGATCGGAA | CTCACCGAGTCACTGCTGT | 45753435 | 45753797 |
|  | CGGCATCAATGTAAGGACCTTCA | TGCCTCTGGCTCTTAGCAAAAA | 45753919 | 45754285 |
|  | TCGTGGATCCTTTACATTTAGCAACAA | CCGAGATCCAAGGTTTCCACATG | 45754489 | 45754860 |
|  | CTCCTCAACAGCTGTAACCCTTT | GGACTCCTGTCTCAGCTGGAA | 45755041 | 45755363 |
|  | TGGGACATCCACAGGACCTAAT | CTCCTGTCTGAAGTGACTGCTT | 45755791 | 45756074 |
|  | GCAGAAGGAAGGCTCTTCTCAT | CCTGCTGTCAGTTCACACAGAA | 45758489 | 45758639 |
|  | GGACACGCACTGGAAAGTATTC | CACGTGTTCACACATGTAATGCAG | 45758903 | 45759254 |
|  | GAATCTCCTTAAGAGCTGTTGCCTT | GCTCTGGTGGTTGTCAAATCTCT | 45761033 | 45761380 |
|  | CAAGAGATCTATTAAATCTTGTGGGCTGAA | CATGCAGTTCCAGAGTGCAAATG | 45761524 | 45761892 |
|  | CCGCTCAAACAACAATGTCCTT | TGTTCAGTTGACATAAAAATAGACCCGTAT | 45762151 | 45762504 |
|  | GTGCACTGCGTCCTCTTC | ACGAGCCCAAAGCCTATGG | 45614431 | 45614711 |
|  | TCTGTGGCCACCAGTGTCTA | CTCAGACAGACCCATCAGGACAAAG | 45688172 | 45688528 |
|  | ACACAGTTTGGCCTTGACACTA | TGTGAACGCTCAGTCAGAATCC | 45632110 | 45632434 |
|  | TGAGTTACACTTTTCCCGCTGAT | GACCGAAATATGCAGCTCTTTCTG | 45750076 | 45750386 |
|  | GGAGGGAGTTGCTTGTTCAGTG | CCATTATCTCCACACCTCAGCTT | 45649828 | 45650158 |
|  | GACTTGCATGGGAGCATGAATAC | AGCAGGAGGAGTCACATCTGTA | 45699493 | 45699864 |
|  | TGAGTCTGGTCCGAAGCAGATTA | ACCAACAAACTGCAGCATACCA | 45757502 | 45757872 |
|  | CAGGTGGAATTAGGGTTAGGGATCTA | CCTTGTCTTTAAGCCAGCAATGAC | 45756340 | 45756497 |
|  | GGTCACACATGTACATCCTGGAA | ACAGTTCCTGAACTCCCAGGAA | 45756740 | 45756954 |
|  | GAGAAGTTTCAGAGCCTGGCTAA | GCATCTTTGTGTTCTCCATATTTTCCATAA | 45703807 | 45704139 |
|  | GGGACAAGGAAGGTACTCGA | ATCTCAGCGCCATGCAGCG | 45613776 | 45614144 |
|  | CAGCAGCATCTCAGACATCAAGT | CGGCCTGATTTCCTGATTTCAAA | 45759466 | 45759832 |
|  | GAAAGAAAGAAATCAGAAAATCGACCACA | AAGCCCGACTTAGATACTAGTGATGA | 45760129 | 45760414 |
|  | CAGGGTTCCATTCCTGCTCTATC | GGGATCAGTTAGTAAAGAAGGAGGAATT | 45760574 | 45760920 |
| ADRB2 | ATTGGCCGAAAGTTCCCGTA | GACGCCTGGAAGCCTCATT | 148206102 | 148206311 |
|  | GGGCATCGTCATGTCTCTCATC | AGCAGGCTCTGGTACTTGAAAG | 148206502 | 148206828 |
|  | AGCCTGCTGACCAAGAATAAGG | GGCCCTCAGATTTGTCAATCTTC | 148206821 | 148207109 |
|  | CGCTTCCATGTCCAGAACCTTAG | CCATAGGCCTTCAAAGAAGACCT | 148207109 | 148207446 |
|  | GCAGAGTGGATATCACGTGGAA | CAAAGAGGAACTGAACTGTACAAATAACA | 148207480 | 148207852 |
|  | CCTCTTTGCATGGAATTTGTAAGTTTATGT | GGTTTTGGAGAAACAGGTGCAAT | 148207845 | 148208214 |
|  | CATCACAGCCATTGCCAAGTTC | GAGGTAAGGCCTGACACAATCC | 148206556 | 148206888 |
|  | TGCGAAGCGGCTTCTTCA | AGCACATTGCCAAACACGATG | 148206178 | 148206552 |
|  | CTTACCTCCTTCTTGCCCATTCA | GCTTTGTGCTCCTTCAAGCAGA | 148206881 | 148207206 |
|  | CACAAAGCCCTCAAGACGTTAG | AGTACCTTGATGGCCCACAAAG | 148207199 | 148207573 |
|  | AAGGTACTGTGCCTAGCGATAAC | CAGACTCAGGTCCTCTAGGACT | 148207566 | 148207908 |
|  | TGAGTCTGCTATATTTTCATGACTTTTCCA | AGTAATTACAAGTAGTTACCTGCGGTTT | 148207901 | 148208275 |
| BDNF | GGGAGTTCCAATGCCTTTTGTC | ACAATAAGGACGCAGACTTGTACAC | 27679493 | 27679864 |
|  | GAATTCCGCCTCCCAAGTTTTC | GGCTAGGCTAGGAATCCTTCCA | 27721125 | 27721321 |
|  | GCGCTACCGATACCCGTT | AGAACTTGGGTGCTGGGATG | 27722110 | 27722479 |
|  | AAAACACGTTTTCATGTTTGTTTTACAGT | TGGCAGAAGTGTTTCATGCATG | 27676383 | 27676757 |
|  | GGGATGGCCACTCAGAAATTCC | CACTGCTGTGAACAAGTGAACAC | 27678081 | 27678434 |
|  | GGATGTGGAGTGTGAGCATTTTT | TTCTGATAGCGTTGGAATTAAAACAATGTC | 27678609 | 27678905 |
|  | TCACTCTTCTCACCTGGTGGAA | TGACAGCATGAGCAGAGATCATTAAAAAT | 27680111 | 27680479 |
|  | CCTCTTCCCACAGAGATACTCTATTATAGC | CTCCTTACCAGACACATTGTTTTCATG | 27680705 | 27680998 |
|  | AGAGACAAGCATCAGCCTTGAAC | GCAAAGCCGAACTTCTCACATG | 27742698 | 27743072 |
|  | GTTTCCTAGGGCTGCCTTCTAAC | CGTAAACAGCGAGGTTAGTCGT | 27743321 | 27743674 |
|  | GGGTCCACACAAACCTCAC | ACCGAAGAGCTAAATAATGTCTGACC | 27721715 | 27722040 |
|  | CGCACGGAGCTAAAAGTGTTCT | GGATGCTTCTTTCTGGGTTTTCTTTTT | 27740910 | 27741216 |
|  | CGTCCTCTCCGGAAGACAGTAT | CCTTAAAAAGCGTCTTTTCTGAGGTTC | 27741820 | 27742149 |
|  | CCCAGATAACGTTACACCAAGTTACT | CGCCATGCAATTTCCACTATCAATAATTT | 27722713 | 27723050 |
|  | TCCTACTTCTCAGTTCTGAGGCAT | TCAAACCAATTTTCCCTTCCTAATCTGAG | 27681569 | 27681936 |
|  | GTTACTCACCTTTATGAAACCATTTTCAGG | GCAGTGGCTAGGAGAACATATTCAATTATA | 27695598 | 27695970 |
|  | GGTCAACATAAACCATCAAGCATGTG | CATCAGCAAAGAATTTCAATTTGTTCAGTC | 27676847 | 27677220 |
|  | GGACCCTCTCCATGAACAGACA | TGTGGCATTTGTGATCATGAGATTGA | 27677474 | 27677826 |
|  | TTCTTGGCAACGGCAACAAACCA | GAATTGGCTGGCGATTCATAAGG | 27679107 | 27679438 |
|  | CCCTTTTAATGGTCAATGTACATACACAA | CCATGGGTTACACAAAAGAAGGCT | 27679376 | 27679549 |
|  | GGTATGTACTCCTTCTGTTCTGCA | TATCATATGACAGCGCACGTCAA | 27722987 | 27723232 |
|  | GAGGGAGCGAGTGAGAATCG | CAAGCTCCGTAGTGCAGGAA | 27722391 | 27722548 |
|  | AATAAGTGTTCAGGTAGCAAACATCTTCT | GGAGTTGGCATTGCATTTACCG | 27677761 | 27678135 |
|  | CTCCATGCTTATACGAGTGTCATGA | GCAATATCGATCAGATGACTAGAAAGTGAA | 27678376 | 27678684 |
|  | CAAAGGCACTTGACTACTGAGCA | CCAGTTTTCTGTCTTGTTTCTGCTT | 27679806 | 27680168 |
|  | CATGTTCATTTTAATGATGTGTCTATGCCT | ACACCACTCAGCTTTTTAAAAGTAGGATAA | 27680931 | 27681254 |
|  | AGATGCTGGAAGGTAATGTGTCTTG | TCACTTTCTCTGGGAACTTTCAGTG | 27743015 | 27743388 |
|  | CCTTCCCTCCTCTACTTCGTGA | GAATCGGAACCACGATGTGACT | 27721465 | 27721771 |
|  | CAGCGGTGGGTGTCTCATTAA | GCCTCGAACGGGTATCGGTA | 27721830 | 27722133 |
|  | CGCTCCAAAATCTGACTCTCTCT | CTATTAACAGGATGGCTTTGGCAAAG | 27742076 | 27742401 |
|  | CGGGCTTCAATGTCATTAATTCAGA | GTACCTCCACGTTTCAGTGGAATTTA | 27695328 | 27695664 |
|  | CCGAGGCTTCTTCCTTAGGGAT | CTGAAGTGGACTTACAAGTCCGAA | 27720817 | 27721184 |
|  | GTATTCAGATGGTACTGTGAAAGACTAGC | GTGAAGGCCACAGCAACCTTCT | 27677151 | 27677525 |
|  | CCCTAAGCCAGTAAAGCAATGAC | CACAACTTAAAAAGTCTGCATTACATTCCT | 27678842 | 27679170 |
|  | GAGAAAGCGGGAACCCTCTAAG | AGAAGGATTCATTCTCTCTGTATCCCT | 27741125 | 27741365 |
|  | ACATTTTCAATGGGAATCGCCATAAAAA | GTCAGTGGCTCTTTTACCCAATAAGA | 27676691 | 27676909 |
|  | CCTTCTTCCCACTTTAGCAGCT | CTGTAAAACAGGATGGCTCAATGAAATTAT | 27680418 | 27680775 |
| CACNG2 | AGAGTCCTTGTTCTCCTTCTGGA | CGTCCACGGAGATCTCCATGTA | 36960440 | 36960579 |
|  | GCATTTGGGCCTTTTCTCAAGTG | ATTTTCCCAATCCTGAGTGTGATTCT | 36962146 | 36962520 |
|  | TTGGGAGGTTGTGAATGAACACA | GTGGCTTCCTCAGACAAGGAAG | 36958588 | 36958865 |
|  | GAAGCCACCTTTCCATCACAGA | GTGGATTTACTTGCAAGAAAATCAGATAGT | 36958858 | 36959128 |
|  | CCTTTTTGCAGTGTCATTGTTGAAC | CTTAACCCACTGTGATGACTTCCT | 36959220 | 36959580 |
|  | GTTAAGGAGCATTAAGGACTGGGAA | GGGATGTCCTTTGTATTTTTCAGGGT | 36959575 | 36959943 |
|  | CAGGGTGTTGAAGCCCTTGAT | CCCGCAGGTCTGAGTAACAT | 36960581 | 36960940 |
|  | GGAGAGAGGCTTAGCTTTTTCCT | GCAGATGTCCAAACGGACAAAG | 36983378 | 36983752 |
|  | CTGGTTTCATTCTCACTGACACTTTTG | AGCCTCTTCCCGTGTGATCTTTATA | 37098470 | 37098844 |
|  | GGGATGGAACTGCTAAAATTTTCCTC | ACACACACACCATATACCTCCTCA | 36957931 | 36958254 |
|  | TGTGTCTGAGAGGCTGACTGAT | CCTCCCAACAGAGCATACACAT | 36958260 | 36958595 |
|  | TTTTTGCTTTAATGTTATCTTGGTTCCCTT | AATGTGGGAAAATATAAACGAGGGAAGAA | 36959971 | 36960158 |
|  | GGATTGTTTTAAATAGGATGCCAGGAGTT | GGTTCTGGGTGTGCTTGAAGAA | 36956846 | 36957213 |
|  | CGCCATGGAAAGAAGCACTGAT | GGAAAGTTATTTTCCCAGGTCAGCAA | 36957223 | 36957582 |
|  | CTTTCCTTTCCCAGGCAAAAGAC | TCCATCCCAGTGTAAGGACCAA | 36957577 | 36957938 |
|  | GAGGAGGGAGAGCTGTTTCATG | CCCAGGTTCCCAGTCCTTAATG | 36959386 | 36959605 |
|  | GGAAATGCCGGTGTCAAGAAAAATG | GGGTCTGTGAAAAAGAGGATCCT | 36957678 | 36958018 |
|  | CTTTGGAGGGCATGAGTCAATTTC | CAAAACCGTTGTGAGAGGTGAGA | 36958642 | 36958958 |
|  | CGGTTTTGCTTTGTTTTTGTAAATGGATTT | CCCAGCGGGTTCAACAATGA | 36958951 | 36959252 |
|  | CTGTTATCCCTGTCGGAGTTGTAG | CATGTTTATCGACCGGCACAAA | 36960486 | 36960773 |
|  | CGGCATTGGCAGATATGTACACTATG | CCAAGCAGTGAAAGCAGGTAGA | 36960886 | 36961260 |
|  | AGGACTGGATCAGTGGCTGTTA | TGTCTCTCCCTCCCTAGGATTG | 36962377 | 36962694 |
|  | GCGAGGGAGTAGAGTATGGGTTTT | TGGGAACCGACTATTGGCTCTA | 37098212 | 37098539 |
|  | GGGTTAAAGGGAACAGAAAAGTAACTCTC | GAAAATGCATGATTTCCCATGTACCA | 36959855 | 36960069 |
|  | TCTTCTCCCAAGGAAGCCTCTTTA | ATTGACCCAGAGAAGCACTAAAGG | 36958052 | 36958406 |
|  | GGGTCAATCCACATGCTTACATG | CTCCAAAGCCATGGACGACAACA | 36958399 | 36958649 |
|  | TGTTTTCTTCCCTCGTTTATATTTTCCCA | GATAACAGCTTCCTCCAGGTTCA | 36960126 | 36960493 |
|  | AAAAAGGACTCACCAAGAAAATAAATGGAG | ATCAAGGATGAGAGCGAAGGGAGAA | 36957012 | 36957366 |
|  | TCCTTGATCCCTTTAGGTCTGGAAT | GCATTTCCTGAGGTTGAGCTCT | 36957359 | 36957685 |
| CDK5 | ACTGTGGGAAAGGAGCCAATTT | CCCATCTCCAGAACCTTCTGAAG | 150750900 | 150751193 |
|  | AGGGAGGGTCAGACTAGAGGTA | CTCAAGCCGGAGGGTAAAGGGA | 150751392 | 150751693 |
|  | GGCTAAGATGTGACATGTGAAGGA | CCCAGGACCCAAAACATTATTTTCC | 150752201 | 150752565 |
|  | TTCAAAAACCAAAGTCAGCTTCTTGTC | GGATGACGATGATGAGGTAGGACT | 150753835 | 150754174 |
|  | GGACATGGAGAAGGGCCTTTAA | ACTCTGAACCAGGTGCTGAAAG | 150752712 | 150753063 |
|  | CTGTAGGCATTGCTGACGGTAA | GAGACCTAGTCGTTTTAGGACTACAAG | 150754803 | 150755083 |
|  | TGAGATATGGAGAACCACAAGGAGA | GAATTTCTCACTCCCTTTGTGGACTTTA | 150750587 | 150750960 |
|  | TGAGATACGCTGGACAGGGTTA | GGGTCACTCGTTTCACATTCCTAG | 150751147 | 150751457 |
|  | CCCTCACCCATTGTGCTCAAAA | CCCATTTGAGTTGACAAATAGGGTCT | 150752039 | 150752328 |
|  | CGAAAGGGACCTCCCACTTAGA | AGGGTACTTCTTGGGAAGAAGGT | 150752478 | 150752842 |
|  | GCACACCTGACGATGTTCTTGT | CCTGAATCCCTTCCCTTGCATA | 150753989 | 150754362 |
|  | GGCCCTCGGTTTTAAGACTCTG | GGAGCACATAAAAGGAATAACAACAGC | 150754961 | 150755300 |
|  | CTGGAGGACAGAAACAGGGTTTT | TCTTTTGCCCTAGGCTTCATGAC | 150753522 | 150753896 |
| CHRNB2 | CCTTCTGACCAGGACACTTCTT | GTGCAGTTCTGCTGGTCAAATG | 154543439 | 154543808 |
|  | GCTAGCCATCCTTGTCTTCTACCT | CTTCTCCAGGAAGACGACCTT | 154544049 | 154544325 |
|  | CCTGACACAATGGTAGCTCTGA | GAAGTCCAGAGGGTGCAGAATT | 154548832 | 154549100 |
|  | AACAGGTTGAGACTATCTCCATCAGA | CTGCTGTGTCTTGTTTGCTTTTAAATTTT | 154551373 | 154551580 |
|  | CCTGCACGTGCTTCGTCA | GCCGCTGCTTTCCAGATACC | 154544456 | 154544712 |
|  | TTCCTCATCTCCATGCTCTTTCAC | CCATCTCTGTCTTCCACTTAGCA | 154548414 | 154548695 |
|  | CTCACGGACACCTTTTCTTGGT | ACTTAACTTGGACAACAGCCATGT | 154550486 | 154550792 |
|  | GTGCTGCATCTACAAAATGAAGATGAC | CCCGATTCTCTGCAGAGATAGC | 154550917 | 154551267 |
|  | CTCCGCCTGCTCATACCA | CCCGTCTTTCCCTTACCTGA | 154540310 | 154540600 |
|  | CTCTCTGCCCAGCTACTGAAAT | GCTGGTGAGTGCCAAGATTCAA | 154542102 | 154542467 |
|  | CTGACCCACACGGTGAGACAAG | GAGGCTATGCTTTCTGAGGTGT | 154549313 | 154549665 |
|  | GCCCTGCTAGTAACTCATGTGAG | CTCTGGTGTTCCTGTAATGTTCCTC | 154549906 | 154550249 |
|  | GCCTCCACCTACGTAACAGAAA | GGACCTGTTGGGATGAATACTAAAAGG | 154551807 | 154552142 |
|  | AAGAGCGCATGCAAGATTGAAG | TGAGATGCACAACGTCATCTTCTC | 154543753 | 154544112 |
|  | GGGAAGAGGCTGGCTTTTAAAAG | TCTGTCTGTCTAGGTAGTGACTACATATCT | 154542614 | 154542984 |
|  | TCCTCTTCTCTTGATGTCAGGGAA | CAGACAAAAATAGTTCTCAGATCACCTGT | 154551195 | 154551439 |
|  | GGGTTAATCTAAGCATTTTGCAGTATCC | GGAAGCACACAAGCATTAACACATT | 154541793 | 154542160 |
|  | GTGTGCGTGCTCAACGTG | GTGGTCTGCGATGAAGCG | 154544245 | 154544610 |
|  | CCTGACCTACTTGTCTTACTCTTGC | CTGCGAGGAGAAACCAGCTTCT | 154540016 | 154540358 |
|  | GCTGCTCTCCATCTCTTGTACC | GCTAAACCCTACTTGGCAGGAATC | 154548616 | 154548970 |
|  | AGACCTGCTCCAGATCCTCTTT | ATCTCAGGGCAGGGAGGAAGAGT | 154549046 | 154549371 |
|  | AAGTCTCCCTTATCAGGAAGAGCT | GGGAAAGTTTCCATAGTGCAGAGA | 154550190 | 154550542 |
|  | TGCTGTGGCTGTACTTGCTTTTA | CCTTTCTTCCTTGCTGAAGACCT | 154550735 | 154550977 |
|  | CATTCACCGACCTTGACTAGACA | TCCCTTTTTCCTGTATTCGCCAA | 154552082 | 154552429 |
|  | GCTTTAACAAGATCATTCTGGGATCACT | CATCCTCCACCCAACACTACTG | 154548104 | 154548478 |
|  | GGGAGATAGAAATACTAGAACAGCTGGTA | TACTCACTAGCTGTCACTTGGTCTT | 154551512 | 154551864 |
|  | TTGAAGCAGAACTTGGGAAGGACA | GCTCTAGCAAGATGAAGGGACTT | 154549602 | 154549962 |
| CNR1 | ACATTGACACGTATCCACTGCTT | TACAGTGAAGAAAGCCTGTTGTTTAAGT | 88853027 | 88853380 |
|  | GTGCTCTTGATGCAGCTTTCTG | TCTTTGGGAAGATGAACAAGCTCATT | 88853636 | 88853894 |
|  | GCCCGTTTTTCAAACATGCACA | GGGATATTTCGTTCTAGCGGACAA | 88875237 | 88875611 |
|  | GACAGGACACAGAGTCAATGAAAATAATTT | GGCAGACGAGAGTTGCATACAT | 88857231 | 88857605 |
|  | AAATCCTTAGTATTTTGCCATCAGACTGT | TGTATTGTTAGATGTTTGTTGAGCTGGTAA | 88849530 | 88849891 |
|  | GTAGGCCACTGCTCAAACATCT | ATAGGCCCAACCACCAGATGAGAAT | 88850118 | 88850454 |
|  | TGGGAATTTGGCCCTTTCTGAA | AGCACTAAACTTGGTGCTTATTGATATTCT | 88850712 | 88851074 |
|  | GCTGTGAAAAGAGCATTGGTACTG | AGAGCTGGCATCTATCTGGTGAT | 88851227 | 88851597 |
|  | GACATTACTGTAACAGTTACAGGACAGAA | ACACATTATCTTCTGGACTATAGCTGTTCT | 88852447 | 88852816 |
|  | CGATCCAGAACATCAGGTAGGTT | TTTGTCTACAGCTTCATTGACTTCCA | 88854153 | 88854486 |
|  | GGACCATGAAACACTCTATGTCCATG | CCCACAGAAATTCCCTTTAACTTCCTTTA | 88854663 | 88854862 |
|  | TAACCACGAGCAAAGGAGAGATTTG | ATTTGTAGTGTTATTGCCAGGAAAGGA | 88851825 | 88852199 |
|  | CCCTCCTATTTCATTGAGACTTTGAAGG | CAAACAATGCAGCCAGTGTTCA | 88853314 | 88853687 |
|  | GCGGGAGGCGGAAAAAGTATT | GGACGGACTGACTTGCTGA | 88875474 | 88875840 |
|  | CAGATTACTAACGAAGATATTGCAGTGGT | TGATTTGGGCTGAATGTATGTAAATAGGTT | 88849823 | 88850180 |
|  | AGGACAGTAACTATAGCGCTACTCA | CTTGCCCTGACCATGCATTTTAC | 88850394 | 88850767 |
|  | GACAATGTTTTCGTCACTTGGGTT | GCATCATCTTGAACATTAATCCACATGTTT | 88850920 | 88851294 |
|  | GCTCAATATTCGGTGCTCAGAAAC | CTGTGTGTGAGTGTGAATATAAATGTGTG | 88852139 | 88852513 |
|  | GTCAATATGCTTTCTCTCTTCCATTCTGAT | TCAGCAAAACTGAAAGGTAGACTTTTATGA | 88852746 | 88853090 |
|  | CCGCAGTCATCTTCTCTTGGAA | CTTTGTTTTTATTCTTCCTGTTTCTCACCA | 88854801 | 88855115 |
|  | GCAGAGCATACTGCAGAATGCA | TCTGTTTGCTCAGACATTTTCCCA | 88853836 | 88854210 |
|  | GGGCATGAATATCTAATTTGCTTTCCT | CACCTCCAGAATTTTAAACATTGCATACT | 88873812 | 88874179 |
|  | CNR1AAACACGTTGCGGCTATCTTTG | GAGAATGAGGAGAACATCCAGTGT | 88854427 | 88854723 |
|  | CATCAGGCTGCTTGGGTATCTC | CCAGCTCTGTCTTGGTAGGTTT | 88851542 | 88851882 |
| COMT | ACTGATGAATGCTTGTATGGGTGT | CACACAGGGCTCTACTGGAATG | 19951553 | 19951926 |
|  | GCCAGGAAGGGTGGAAAAGATAG | CACAGCTGAGTAGCCACAGTAG | 19950806 | 19951171 |
|  | GCGATGGTGGCACTCCAA | GCTCGCAGTAGGTGTCAATGG | 19949964 | 19950302 |
|  | GCTGCTTTGAGTGCACACACTA | CCAGTGTTAGTAAAGAAGTCATGATTGAGT | 19956153 | 19956472 |
|  | CTTAGTACATCCTTCTCAACTGCCATT | GGTCAGAAAGGTGTGAATGCTG | 19956558 | 19956828 |
|  | GAATCAACCCTGTGCTAGCTGA | CAGCAAAGGAACTTTTTGCTTTCTG | 19957119 | 19957429 |
|  | CATTAGAAGCAATGTCTGTCCTCACT | CACTTTCCTCACTCCACTGAGT | 19938947 | 19939308 |
|  | CCTTCAAGAGAAAGTAGGGAAGTATGG | GGCTTCTTCTGAACGCTACCTA | 19938224 | 19938598 |
|  | CCTTATCGGCTGGAACGAGTTC | GCTTCTCCTGTAAGGGCTTTGATG | 19950151 | 19950415 |
|  | CTGACATGCTAACCTCTCTGAACT | GAATGTTTGGTGCCCAAGTCAAG | 19956393 | 19956618 |
|  | CCGGACTCCTGAGCAAGA | TAGCCAGCGCTCTCACCTC | 19929065 | 19929436 |
|  | TGGAAGCATCTCCTTCCCTACT | CCCAGGAGTAGAATCTTGGCTAG | 19956897 | 19957187 |
|  | CCAGCCCACTCCTATGGATAGA | CTGCTGTTCGTGACTCAGAAAC | 19957211 | 19957545 |
|  | TCAGGTATCTGGACCTTGGTCA | CTATTTACACAGGCAGAGTGGACAT | 19948644 | 19948920 |
|  | CCTGCACAGGCAAGATCGT | TGAACGTGGTGTGAACACCT | 19951080 | 19951385 |
|  | GGCACCCAAGTCTTGTACAGTC | CCTCCCTGTATTCCAGGAACGAT | 19955875 | 19956200 |
|  | AGGCACTAGGAAGAAGAACTTCCA | CGCTAAGAGTCCCAGGCTTTTT | 19938543 | 19938917 |
| CSF1 | TTCTCACCCTTCTTTCCTCCTGA | GGTCTCTTAGCGGAGGACAAAG | 110472804 | 110473085 |
|  | CTTAGGGAAGGGCAGTGAACTT | GATGCCACCAAGTGCTCAAAAA | 110473181 | 110473388 |
|  | GGACGCTGAGGAGTGAAAGAAC | GCTGCACACCTTTACGGGAAAT | 110471848 | 110472001 |
|  | CTGGATTTGAGGGATGCTGTATCC | CCCTATTTTCCTGCCTGCAACT | 110456733 | 110457107 |
|  | ACCTTCCCTATATACATCCATCGTGATTAA | GGGTGTTATCTCTGAAGCGCAT | 110459625 | 110459999 |
|  | GGCCAAGGGAATTTGACAAATAAGATG | ACAGAACAAGCACATTCATGCAC | 110464388 | 110464762 |
|  | CTGTGGTTCTTTCAGATGTGGTGA | TAGTGCCCATTGCAGAGTCAAG | 110465773 | 110466147 |
|  | GGCACTAATTGGGTCCCAGAA | GGCAGATGGATGGTCTGTCTT | 110466141 | 110466419 |
|  | AGGCTCTCCCAGGATCTCATC | CGCCTCCACCTGTAGAACAAG | 110466443 | 110466808 |
|  | GAAGTTGGGAGGACTCTTGCAA | GTCTAACTCTACGGAGAAATCAGGAAAA | 110467278 | 110467643 |
|  | CATGCCATCTTCCAGTCAGACT | TCTCTCTGAGCTGGTCAGACAA | 110468479 | 110468789 |
|  | AGAGAGAGTACAGTGGGACTGTT | ACAACAGCAGGCACTCATCAAT | 110468783 | 110469151 |
|  | CCTCCCAATCCATGTCCTCAAC | GCCTCCAGGGCTCACAATAAAT | 110471400 | 110471710 |
|  | TACACCCTCGGCTCACCTAA | GTCAAAGGCCAGGTCAGGAGAA | 110472063 | 110472434 |
|  | CCGGTCTTTAGGCTGTGTTGTT | AGAAGTGGATGGGAGCTGGATA | 110472484 | 110472807 |
|  | CCCTTCTGTTCTCTGAGAAGTCAA | CAATACGAGCAGATATGCAGATGAATG | 110473448 | 110473822 |
|  | CCCATGCTGCCACTATTTATTGTG | CTCTCTCTGGAGACAGGTCCAT | 110471675 | 110471942 |
|  | GGAGGACTCTGAGGGAACTGA | GAAGATGCTGAGAGGAAGTTGCT | 110465912 | 110466286 |
|  | CCCTCTACCATCACCTCTAACCA | AGAGGCTGTGACTAGAAGGCTAA | 110472865 | 110473236 |
|  | CCGGCAGATGTAACTGGTAC | CGAGGAGTGGCTTCTGGAAAG | 110466318 | 110466536 |
|  | GAAGGAAAGGGTCGGTCCG | AGAGGACCCAGGCAAACTTTC | 110453148 | 110453512 |
|  | CTGAAGGCTGAGTTGAGCTGTA | TGGTACTGTCAGAACTACATTAGCAAAG | 110458197 | 110458571 |
|  | CCATAAGCTCCAGGTTCCTGTT | CTGGTAAAGGTTGGTGGGATGA | 110459887 | 110460239 |
|  | CCTGCATGGGCTGTTCTCTTATC | GTCACTGCTAGGGATGGCTTT | 110465504 | 110465846 |
|  | GTTTTAACTCCGTTCCTTTGACTGAC | CCAGTCCCTTCCCATTGACCTTATA | 110466652 | 110467026 |
|  | AGTTAGACAGTTCTGGGTCTTTTCAATC | ATAGCTGAGGACCTGGAAAGGA | 110467636 | 110468008 |
|  | AAGTCCCTCAAACGTTCCTTATATGATATG | GGTGAACATAAACTGCACTTTGGTT | 110468647 | 110469021 |
|  | GAAGTTGTGTGAAATCCTAATTCTGTCATC | GGCTTACAATCTTAGGGATGTGGAA | 110469069 | 110469443 |
|  | AGAGAGGAGCCTGAAGTTCGT | AGTGCAATGGCTTCCTTCATGA | 110471937 | 110472266 |
|  | ATTGCACTGTGAACACTGTACCT | TCAAAGTGCAAGATGCAGAAACC | 110472259 | 110472534 |
|  | CACTTTGACATTCCCAAGAGGGAA | GGGTGAGAGTTGGAGGTCATCA | 110472527 | 110472861 |
|  | AGCCTCTATATTTGATGCTAGAAAACACA | CGTGAGCCAATGTCCTCTCTTG | 110473230 | 110473490 |
| DLG4 | CCCGAACCATGGAATATCCTAAATT | GGGAGGAACGAAACTCCGATG | 7120192 | 7120530 |
|  | CCCGGAGAGATTCGGAGATG | GTACCTGGCACCAAGAGGGAGAT | 7123287 | 7123609 |
|  | CTCGAATCGGCTGTACTCTGAG | TGTCGTCCTCTCTCTAGGTCAAC | 7099637 | 7099911 |
|  | AAAGCTCAGACAAAGCCACAAATG | GGGCCTAAAGGTAGTAACTCATTTTGT | 7106384 | 7106752 |
|  | ATCACTAGCATCGATGACATGCA | TTGTTATGAGTTATAGTCAGCGTTTGGT | 7097715 | 7097985 |
|  | TCACCCACATCTTCCCGAACTA | GGAAGGACAACTCCTTATTGAGGAG | 7097130 | 7097481 |
|  | CCAGGGTTCTGAAGGGAGAGTT | CGTGTTTCTTGACATCCATGTCACT | 7095114 | 7095461 |
|  | CCCTTCTCGGAGAAACCCTGTA | CTCCCACCAGGTCGAGAAGACT | 7096669 | 7097043 |
|  | AAAAAGCCACATTTAGACCTTCCA | CCCACACACATTCCAGAAGTCAG | 7093408 | 7093695 |
|  | CCCTTGATGAGCTTGATCTCCA | GTGGCACTGACAACCCACACAT | 7106751 | 7107104 |
|  | GGATAAAGGAGATGAAGATGCCTTCA | CTTCCACCTAACTTTGGCTCTTCT | 7100135 | 7100471 |
|  | CTCTGGGACATCTCTCTCTTCTCTC | CTAGGGCAGGAAGTCCCAAACA | 7122158 | 7122532 |
|  | GGGTCTTCCTACTGTGAAACTGT | GTCCGACCTAGAGTCTAGTCCT | 7122714 | 7123066 |
|  | ACACAAAAGCAGTGAGACAGACATT | CGTGTGTGTGATGTGGATTGAG | 7107402 | 7107738 |
|  | CACACTCATGGAGGACACCATA | GGGAGATGAAGTGACAGTGTGTT | 7121720 | 7122034 |
|  | GGCTGGATCCAGTTAGAAAGGAAATAAATA | ATGGCTTGGAGAGTTGGAAAGAG | 7093887 | 7094246 |
|  | CTGAAGCCCAGACCTGAGTTAC | TTGCAGAAGGAAGGAGGGAGTAA | 7107113 | 7107486 |
|  | CTAGAGCAGGCAGGGTGGAGAA | GCTGAAATGGCCCTTAGAACTCTTC | 7099434 | 7099761 |
|  | CTGGTACCTTCTGGTTTATACTGAGC | GAAGGCATCTTCATCTCCTTTATCCTG | 7099785 | 7100159 |
|  | CTGGACACTGTGCTCCTCAATA | GTTTGATTACGACAAGACCAAGGACT | 7097445 | 7097808 |
|  | GGGCACCTCTTTCTAAGCCATC | TTTGCTATGTGCTCCAAATAGAAGAATAC | 7094484 | 7094786 |
|  | GGGTGGGAACAAAATGAGTTACTACC | TTTTCATCACCAAGATCATTCCTGGT | 7106718 | 7107065 |
|  | CCACCAGGGTCTCCTACCTT | GCTGTTGGGAGAGTGGAGAGA | 7111377 | 7111739 |
|  | CGTCACTGTCTCGTAGCTCAGA | TCCATGTATCCCACAGGGTTGA | 7096996 | 7097335 |
|  | CTGAAGAGGGACAGCTACAGGGAT | TCTAGCCCATTTTCTAGCCCAGAT | 7106127 | 7106499 |
|  | CCATTTTCCAGGCCACTAACCT | CCACCATTTTTCTTTGCCGGTTT | 7093100 | 7093461 |
|  | CCAGACCCTGCCTTAGGAGA | GGTCTGGACTGAATTGCCCAA | 7093600 | 7093974 |
|  | TGACCTGGGAGCTAGTGAGATG | CAACTCCAATCCTGGAGGATTAGG | 7121948 | 7122217 |
|  | CCCAATGAGAATTGCAGAAAAAGGG | CTGAGATGTAGGCCATTCACTAACTC | 7122478 | 7122773 |
|  | CTCTTCTGTCCCATCCTATCCCAT | CCTCTTGTCACACACAGACCTT | 7123010 | 7123384 |
|  | GGAAGAAAGGGAGAGTGGGTACA | GCCTATGGTGTCCTCCATGAGT | 7121373 | 7121744 |
|  | GAGAGAGAGAGTTAGAGAAAGCTGGA | GGGATTTGATTGAAGTAGGCAGGAT | 7096177 | 7096516 |
| DRD1 | CTGACTCAAGAATGATAATGAGGCACT | CCAACTTAAGAAGAGCCCAGCATT | 174867436 | 174867807 |
|  | CACATGAACATTTAGAATCTCACAGAGTCT | AATGAGATGGGTTGAGTTTGCTGTA | 174868048 | 174868415 |
|  | AGAGTCTCACCGTACCTTAGTTTCTTAA | CTCCAAGGAGTGCAATCTGGTT | 174868666 | 174868960 |
|  | CAAAATGCAGTTCAAGATGAAGAAAGGTA | CCTCTGTAATAAGCTTTTACATCCCTGT | 174869218 | 174869510 |
|  | CACACAGAGGTTGAGGATGGATG | AAGATGAGGACTCTGAACACCTCT | 174869755 | 174870105 |
|  | CTGGAGCTTGGAATGTGGACTT | CTCTGGGCTCTCGAAAGGAAG | 174870732 | 174871053 |
|  | CTTGAGTGGCAATCCAAGTCAATC | CCATCTGCTTAAGTCTTGGAGAAAGAC | 174870266 | 174870638 |
|  | GCAGCATATACTTTTCATTACCACCTAAGA | CCATTTAACTAGCACTTTATAAGCCAATGA | 174867744 | 174868118 |
|  | CTTTGCTGGGAACAGTGTTAGC | CCAAAAGCTAGAGGAGATTGCTCT | 174868358 | 174868728 |
|  | CTCAGAGGAGCCCACAGCATGT | CGGTGATCATGGGTGTGTTTGT | 174868906 | 174869279 |
|  | TGTAGATCCTGGTGTAGGTGACAA | GTAACATCTGGGTGGCCTTTGA | 174869448 | 174869816 |
|  | CAGTGAGGATACGAACAGAGAAGTC | GTCAGAAGACAGATGTAGAAATCAAGAGTG | 174870024 | 174870330 |
|  | GCAGCTCTCCAAACGCCTTAAA | AATCTCCAGCTCTTCAAGGAAGTG | 174870570 | 174870929 |
|  | ACCTGGGCAGCTTCTTTTCTT | GAGACTGGCGAGGTAACCA | 174871010 | 174871379 |
| DRD2 | TTTAGGGAGTGTGTGCTCTCCTA | GAGTACCAGGCTACAGGACCTA | 113288558 | 113288930 |
|  | GTCTGGATCTCAAAGATCTTGGCA | GGTGTTTGCAGGAGTCTTCAGAG | 113283382 | 113283713 |
|  | CCCACCTGAGCATAAGATGAGC | GAAGGACATGAATGGGCTCTTGT | 113286064 | 113286414 |
|  | CCTGGAAAGTAGAGGTCCACAT | TATCCTTGGCACCAAAGATGCA | 113280855 | 113281170 |
|  | CGGTGCTTACCTTCAAGCC | TTCAGTGCCGAACCGGCAG | 113345788 | 113346123 |
|  | GTCAGCAGTGGAGGATCTTCAG | CTACAGTGCTTCTGTGGCTACA | 113281448 | 113281766 |
|  | TGGAAGGTGACTCGTCAAAGTTT | GACATCAGAGGTTCTTTGAGGGA | 113280330 | 113280614 |
|  | GAGGCTGACGATCAGGTAGTTG | ATGCCAGGAGTTGGTCTTCTTC | 113295146 | 113295485 |
|  | GGACTCAGTCCAGGGTAGTGAT | GGTGTCTCTGGTGTGTGCATATT | 113287446 | 113287801 |
|  | CAGAACCGAGGTGTCTCACTTC | CCCAGTCTGAGTTCCATGGACT | 113284908 | 113285278 |
|  | GGGAGAGGATTCCCTTCTAAGTG | CGCCCTATGGCTTGAAGGT | 113345542 | 113345814 |
|  | GAAAGTGGTTTTGCGTCAGAGT | CCCAAGCCAAAAACCTTAGCTC | 113280557 | 113280912 |
|  | GCAGGGTGTGAACTGTCCATC | TTGCGAACCGTGAGCAGGAA | 113281016 | 113281383 |
|  | CCACTCTAAAACTCTGTTTGCCCAT | GAAGAATGGGCATGCCAAAGAC | 113283075 | 113283438 |
|  | GGATCCTGACATGGGACAGAAG | TAGACTCTGTAACATCACTATCCATGCA | 113280054 | 113280391 |
|  | TAAGGAAACAATCTACCCATTTCGTAAGG | CCACTACAACTACTATGCCACACT | 113294912 | 113295278 |
| DRD3 | AGAAAAAGAGAGGAAAGAAAATGTTCCAGT | GAACAACGGTACACAGGGAGTA | 113878468 | 113878842 |
|  | CGGTGTCAGATTTGTGTCTCCA | GCTCCTTACTGAGTTCTGATACTTGC | 113858229 | 113858602 |
|  | CAGGCTCACTACTAAGTAGTTGGTG | CAACAGTGCCTACTATATAGAAAATGCTCA | 113890627 | 113891001 |
|  | ACCCATTTTTGCCTCTAAGGTGA | GAGGAAGGTGAGAGTTATTCAAGAGAAATT | 113897648 | 113898021 |
|  | CAGTTAAGTGGCTTGTTCAGCTT | AAGCTCAGCTTAGAAGTTCGAAAACT | 113849752 | 113850094 |
|  | AGTCATTCAGCTGTGCTGTGAAT | GGATCTGATCCCAGGAGTTGAAC | 113866204 | 113866574 |
|  | GGGACAGGATGGTGACTGTTTT | GCCTACTATGCCCTCTCCTACT | 113890389 | 113890752 |
|  | CCACTTAGAAGAAAAAGAAAACCATTTCCT | TAATAGGGAAGCTGGAAAAGCAGCA | 113897335 | 113897706 |
|  | CCCAGCTTCAAAGATGTCGATAATC | TCAGGAACATTCAGAAGATAAACAATACCC | 113850033 | 113850314 |
|  | GGAGGACACTGCACAGTCTTTC | GACAACCGTGAGTCATTCTCATGTTA | 113847479 | 113847841 |
|  | CTTCCATGATGTCTAACATGCCATTAATTC | GGTGGGACTTTAAGGGCTGATTA | 113917981 | 113918355 |
| DRD4 | CGCCCACCAACTCCTTCATC | GGGTCCCTGGATAGAAGGAC | 637504 | 637874 |
|  | CCCTTCTTCGTGGTGCACAT | GGAGCCTTCACAGCTGAGTTT | 640424 | 640724 |
|  | GTCTCTCCCTGCGCAAAATTC | CTCACGCACACGAGCGAGTT | 637117 | 637477 |
|  | TTAATTAAACAAATTCCTTCCCAAACTCAGC | CCCATTTGTTTCTTCCCATCGGA | 640682 | 641001 |
|  | CGAGACCCTAACTCAAAACAAAGGG | TGATGGCGCACAGGTTGAA | 639241 | 639535 |
| EGR1 | GCCGTTCCAGACCCTTCAA | GGGACATCAGCTGCATCTCG | 137801125 | 137801487 |
|  | CCCACCATGGACAACTACCCTA | CGGCTCTCATTCTAAGATCCAGGA | 137801529 | 137801855 |
|  | TCTCTCTCCTGCCAGAGTCTTTT | GCTCAGGGAAAATGTCAGTGTTC | 137802431 | 137802796 |
|  | TGACTTCAGCTGCCTGAAACAG | GGTTCAAACTTATTGTCACAGCATCATC | 137803978 | 137804352 |
|  | GTCTTGGTGCCTTTTGTGTGAT | TCACTTTGTTTAAGCAAACACAAGTACA | 137804533 | 137804901 |
|  | ATTAAGGCCTTTGCCACTCAGT | TGGTATGCCTCTTGCGTTCATC | 137803012 | 137803384 |
|  | AGACAAAAGTGTTGTGGCCTCTT | CCCTTTCCCTTTAGCAAATTTCAATTGT | 137803416 | 137803780 |
|  | CCTGCACGCTTCTCAGTGT | CGTTGCTCAGCAGCATCAT | 137801318 | 137801580 |
|  | GGGATTCTCCGTATTTGCGTCA | CAGCACCTTCTCGTTGTTCAGA | 137802235 | 137802486 |
|  | ACAGCAGTCCCATTTACTCAGC | GGACTGGTAGCTGGTATTGAGG | 137802731 | 137803077 |
|  | GCCAAGCAAACCAATGGTGATC | CACAATTGCACATGTCAAGCCAT | 137804292 | 137804588 |
|  | ATGAACATGCAGTTCATTATTTTGTGGTT | AAAACCTCACAGCCCTCAATAGG | 137804834 | 137805060 |
|  | TCGGACATGACAGCAACCTTTT | AAATCAAGTTCTTTGGATAGAGGTGAAGAA | 137803723 | 137804040 |
|  | TGCGACATCTGTGGAAGAAAGT | GGACGGGTAAGAGGTAGCAAC | 137803330 | 137803494 |
| ESR1 | GACCTATGGAGAGCAGCAAGTT | GGCTGTGCCTCAGAACTGTATC | 152420833 | 152421207 |
|  | CTCTATTCCGAGTATGATCCTACCAGA | CAACAAGTTCTTGAAAAGCTATTGACTCT | 152265526 | 152265697 |
|  | CGTCCTCCAGCACCTTTGTAAT | GTTCCCTTGGATCTGATGCAGTAG | 152128743 | 152129110 |
|  | GAACCAGTGGATTTTTATGAATGTGAACC | GACATTATGCCTTTGGAGTGGGTA | 152382022 | 152382354 |
|  | TCTTCAGTGCCTATTGATAAGTGAGACT | AATGAAAAATCGTTCTCCAAACTGATGAC | 152022866 | 152023240 |
|  | GCTCGGGTTGGCTCTAAAGTAG | AAAGTGGTGCATGATGAGGGTAAA | 152419819 | 152420168 |
|  | GGCTTTCTAGATGAGTGGCCATT | AGGGTGCTATAATAAACCCTTGACCTA | 152420218 | 152420586 |
|  | GGCCTGGTCAGATTACGTATGC | GGCTGGAATGAGCCTTTCTTTTT | 152421460 | 152421642 |
|  | TATTTGGAGGAAAATGGTTAATTCTGGGT | GAAATCAAATCAAAGACTGCACTTCCTTAG | 152422931 | 152423259 |
|  | GTGGGTACTGGGAGTGATCACTA | GCCATAATAGAGGCAGCAATTACTTAGAAA | 152423494 | 152423857 |
|  | CCATTCCACGCACAAACACATC | TTGCTTTCTCAAAATGCTAAAGCTACA | 152126795 | 152127007 |
|  | ATAAGTGACATTATGCCAGTTTCTGTTCT | GTGAACTGCTAGCAAGAAGTGGA | 152424093 | 152424457 |
|  | CCCACTCAACAGCGTGTCTC | CTCCCTAGGCTCTCCCTTCTC | 152129341 | 152129587 |
|  | GTTTGCACTTCAAGAAGGACAGAAA | AAACACAAAATTCCCATGGTGTTATTCC | 152201629 | 152202003 |
|  | TCTCTTGGTATGTCTTGTTTGGAAAAGT | CATGTTAACCCAGTCAAATTAAATCCAGA | 152421816 | 152422145 |
|  | TGAATGACAGACAATCTTATGTAGCAAAGA | TACAAAGAGATTTCCAAAGTTGCTGGA | 152422336 | 152422708 |
|  | ATGACCCTCCACACCAAAGCAT | GCGGGTGCAGTAGCATCAG | 152129054 | 152129387 |
|  | CCATGAACACTCTGGGTCTCCTA | ACAAGTGCACTCTCCATATGCAG | 152415446 | 152415814 |
|  | AAATGAAAGCTGGTTAGCTTTGAAAATTTT | CAGTAAGCCCATCATCGAAGCT | 152265217 | 152265585 |
|  | AAAGTATGTTCGTATTGCATTTACTCCATC | TCTGAAGCTTCACCGAGAGATGA | 152332657 | 152333024 |
|  | CAGGATAAAGTGGATCTGCTGCAT | GCTGATTCTATGTCTTCCTTCCTCAGT | 152163645 | 152164013 |
|  | ATCCATACACTTTTGACTGGCATTCT | CTCAGTGTGTGAACTAGGCAGA | 152126478 | 152126849 |
|  | TCTGCCCTATCTCGGTTACAGT | CCTTGCCCTGACATTGGCTTAA | 152128354 | 152128728 |
|  | GCTCCCACACGGTTCAGATAAT | CAGAAGATGTGCCACTAAGAACTGA | 152420112 | 152420276 |
|  | AATTTAAAGTGGCTCCTTTAATTGGTGAC | GGAACTTATCCCTCATATAGGGAGACT | 152420520 | 152420892 |
|  | GCTACTAGAGAACAAGAGGGAAAGTAG | AAACCAAACCCTGATTTTGGATTATCTCTA | 152421148 | 152421522 |
|  | GCAGAGAACATCAGATGATTGAAATGTTC | GGCAGAAGTGGACTCTACTGAA | 152422642 | 152422992 |
|  | GAAGACCCTATCAATGTAGGTTGCA | GCAGATCTGCCTGTGAATATTAGACAT | 152423194 | 152423554 |
|  | TAAAGTGTGGCCTCGTTTTTAGTCA | GAGTATGTAGTGCACAAAAAGCATTGTTTA | 152423792 | 152424162 |
|  | TCAATGAGATTTTCCAATCCTAGTCAAATG | AGTTACAATATGCTTGTAAACAGTAGCACA | 152011556 | 152011877 |
|  | CAGCTCATTTCCTTCAATTTCCTTTGA | GGTGATCACTTAACTGGACAATCAGAAAT | 152421582 | 152421883 |
|  | ACATTGCTTGTTTATCAGACAATTGAATGT | CAAAATTAGCTGCCCTGAATAATTTTCCTT | 152422076 | 152422406 |
| FAAH | GGGACACTGGTATACCTGTTTTGG | GGTTACTGCAGTCATAGCTGGA | 46870937 | 46871276 |
|  | GGGCTGAGTAGTTTCTCTGATCTC | TCTTGATCAGCCCTCTATATACTTGTCC | 46874044 | 46874418 |
|  | ATCAGCAGAAACAAACGGCATGT | ATGCCTGCCCAAACCAAGAGAG | 46875908 | 46876278 |
|  | GCTAACCCTATCCTGATGCCTGTA | GTGGAGGGATCAGGACGAAGAGA | 46879085 | 46879457 |
|  | GCCTGAAGGGCTGTGTCTATG | GGTTCAGCGGTCCACATTCAT | 46871718 | 46872081 |
|  | GGGTTTGAACTTCCCTCTCAGA | GATGGGCATCTAGGCAAGTAAGG | 46877137 | 46877511 |
|  | GTCGCCCACGAGAAACAGTC | CAGCTCGTACTGCACCATGA | 46859772 | 46860038 |
|  | ACAGAGCTGCATTCTCTAGGGAT | GACTAACTCCAGGCCAGGTTTT | 46867621 | 46867995 |
|  | GAACCCATGGAAGTCCTCCAAA | GGCCAGTGGTACTGTAATGCTT | 46871296 | 46871598 |
|  | CCTTTCTCTGGCTGTAGACACA | TCCTTCAGGTCTTGCTTAAGAAAGG | 46878635 | 46879009 |
|  | GCAGCCCATGGGTATGACATAG | GGATCTGAGTCAGGTCTCAAAGG | 46879467 | 46879841 |
|  | ACACCAATGTTCCACAGTCCAT | TGCCTCCGATATCAGTGCCTAA | 46871150 | 46871402 |
|  | CCCTCTCCCTGGGTATACTTTAAAAG | CTCTGGACCTGATACCTCCTCTA | 46870575 | 46870926 |
|  | CCACAGTCCCTGAGTTAAAGAGC | GAATAGTGCTGATGACAGGAAAATCAATC | 46877739 | 46878113 |
|  | GTAGGCAGCAGCAGGCTGAA | AAAGTGCGGAATATCTTAGGGCAA | 46859995 | 46860369 |
|  | GCGTGGGTCCTAGTTTCCAAAG | GTGAGGGAGGTAAGCAGACAAG | 46871601 | 46871900 |
|  | CTCCCTTGCCCTTCAGAGAA | AAAAAGCCTGAGGATTTCCAAATGC | 46872018 | 46872232 |
|  | CACAGTGAGTTTTCATGGGTCAGTAA | AGGCCTTTGTTGGATTCAGCTA | 46874610 | 46874984 |
|  | GGCCTGAAGAAGGTTGACGT | CAGAACTGGACCTTGCCAAGA | 46876417 | 46876713 |
|  | CGAGGCCCAGATGGAACATTAC | TGAACCGCAGACACAACTCTTC | 46878829 | 46879200 |
|  | CCCTGAAAAGCAGTCATCCTGATG | CAGACGAGGAGCTGTTTCTTGA | 46879226 | 46879529 |
| FKBP51 | TTGCCCGTGCTCCGCTAAC | ATCCGGACAGGCGGATCGA | 35656621 | 35656840 |
|  | GTGTTGGAGGCAAGAGACAAAC | TGGTCAACATCTGGTTTGTCTTCAG | 35558769 | 35559139 |
|  | AGCAAAATGAAAAATCCTGCAACCTT | GGGTTGCTTCACTTTGAGGATTT | 35547706 | 35548080 |
|  | CTGAAGGGAATTTAGTTTTCTCATCTCTCA | TAAGGCTGCAAGACTGCAGATC | 35544553 | 35544885 |
|  | CTAGGGATTCTCAGACCATTCTTTGAG | GATGTCTTGGGATGGACTGTGT | 35604651 | 35605025 |
|  | TGCGCACAGCCGATATATTCAT | GGTTGCCCTTGCCTTCACATAT | 35586762 | 35587063 |
|  | CCACTCACATGCATGTGGAAAG | GTCCTCAAAGACCAGCAAGAGAA | 35550263 | 35550594 |
|  | AGAGTAAGACCCTCTCTCTCTAAAAACAAT | GCCTTCTACCATTCTGATTATTCAGAACTT | 35550846 | 35551155 |
|  | CTGAGCTGTTTTACTACACTGAATCGA | CCAAGTTTCTTTCCAGCCTGAATT | 35552867 | 35553241 |
|  | CCAAAAACACTTGTTGAGTGCCAA | AAATGGTGAGAAAACAATCATGTGGAAAAA | 35554479 | 35554661 |
|  | CCACACCTGGCCTATTCTTTAGTC | TAGTTGCAACAGAGACTGGTTCAAA | 35548606 | 35548784 |
|  | ACATGCTCATATGAGAAACGCTG | GGACACATTGTCTCAGCCCT | 35549077 | 35549425 |
|  | CCTCACCCAAGGAGTCTGAGTA | AATGACCTGTATCACACACTTCTGAG | 35549701 | 35550011 |
|  | TCAACTTCCAATTAGCGAAAAGTCTGT | GCCGTTTATGACTCTAGGATTTGATGTTTT | 35551276 | 35551627 |
|  | TGCTCGCTCACCAGATCAAC | CTCTGTTTGCAACTAAGAGTTACATCC | 35695971 | 35696345 |
|  | AAAACACTGACCAGGTGTGAGTTAA | GTTTATACCTAAATGGGTAAAGCCTCTCT | 35551849 | 35552142 |
|  | AAGTTTTACAGCTAACGCTAGGACTAC | TGTCTGTACAGTTACTTAACTTTGTTGCT | 35552375 | 35552712 |
|  | CCCATTTTTCCCAATTCTGAGCTTATTC | GTGTTTTCAGAATTTGAGCTGAGACAAT | 35553326 | 35553625 |
|  | AACTCCCATTCTCCCTCTAATTCTCA | TGTCTTGATGAAGTCACTAGAGCATATGA | 35553872 | 35554246 |
|  | TGTCTGTGGACTTCTACACAAATTGTTTAA | CACCTACGAAATATGAAGAGCAAAAGC | 35541504 | 35541865 |
|  | GGATTTAAAAAGGTGAGATGTTCCAGGTT | AGTTGTGAAAGAGTTGAAGACAGCT | 35542121 | 35542370 |
|  | AAAAAGCTGCATGGATCCTAAATGAC | GAGTGGCTTTCTACCACACACA | 35542609 | 35542965 |
|  | CCCAACTGTAGGAAAGGAGAAATTCAT | GGACAGAACAGTGTTTAATGTAAAGTTTGT | 35543187 | 35543544 |
|  | GTTGTGCTCCTTGGCCTTTTTC | AGAGAACTTTGGTAAGCTGTTTACTGAATT | 35544831 | 35545084 |
|  | CAGGGAGGACAAAAACCTAGATCTT | GAACTGCTTTAGTACCTCAGAAGCA | 35551090 | 35551338 |
|  | GATCTGATTCTCTATAGCAAGCTTAGCT | GCCCATACTTTCTAGCTGAGTATGTAGTAT | 35587800 | 35588172 |
|  | AACACTTGTTCTTGAGAGTTTAGAAACTCA | ACTCTTATGGATCACCAGAAAGCATG | 35553184 | 35553390 |
|  | GTTTCCGTTGTTGGATTAACTTCACT | GGGAAATGGTATTTGCTACATTGTCG | 35564912 | 35565285 |
|  | AGACTGGCACTAAAATCAAAGTCTGTC | CTTGTAGCTCCCTTCTTGCTGT | 35549948 | 35550319 |
|  | AGGTTCATAATAGTATCACTCTTGCAGTTG | GATTTGAGGCTTTTGGCCTTTGA | 35552643 | 35552927 |
|  | AGTTTGTTTTGAAATTTGTGCATGTCCT | TCACTCACTCCTAAAGCATCTTGC | 35554179 | 35554537 |
|  | CTGGGCTATGTTCTTTGGTGCTA | AATCCTTTTGAAAGACTAAAGAATAGGCC | 35548268 | 35548642 |
|  | CGCCTGACCTCTCTGATGTCTA | AAGAAGACACTTCACGTATTCTTGTATCAA | 35541257 | 35541574 |
|  | CTAGTGTTTGGCACTTAGTAACCATCA | CATTTTCAGTATCTAAGCTTATTGGCCCTA | 35610374 | 35610748 |
|  | GCTGTGTACATGTCCTGTTTTCC | CCTATGTCCCTCTTTTCTCCTAGGATC | 35696251 | 35696420 |
|  | GGGTCTCTGTCCTGATAACTGG | TCCCTTTACTCAGCCTGTCACT | 35688035 | 35688395 |
|  | CCAGGGCTGAGACAATGTGTC | GCATGTCCTCTTAGACCTGCTCTAT | 35549404 | 35549758 |
|  | GTTTCAGGTCGGCATTTTCTTTCT | TGCTCCCAATGATAGCAAAAGGTT | 35550537 | 35550910 |
|  | GAAAGACAGGGTGGACTCTTCTTAG | CTCATAGCTGACAGTGTTCTGCA | 35551562 | 35551907 |
|  | TCCCACTTAAAAGCTGAGTACAATTCAA | CCTGGAGGCACCTTTATAATTATAGGTAAA | 35552075 | 35552442 |
|  | ATTGCAATACACTTCCACAAAACTGATTT | GCCTTCTCCATGTCAAGCAGAAA | 35553558 | 35553931 |
|  | GGCAAGTGCTAGTTCCAAACAC | CCCAGCGTTTCTCATATGAGC | 35548727 | 35549101 |
|  | AGCTTTCCCAACAGTTTAGCAAGT | ATTTGGCAACCCAAGGAACCAT | 35541804 | 35542178 |
|  | GAGATTTAGGCTACTGGCTGTGTA | GCTTACAATATTTTTGACCTGGAGTGGAT | 35542311 | 35542674 |
|  | GGGTGTTTTAGGAATGACAGAATTACTCC | CTTTCATAGCACTGCAGAAACCTTTAAAAA | 35542902 | 35543254 |
|  | GTTTTGCCATTTGCTTCCAGAATCA | AGGAGCCAGGAATTATTTCACTTCTTTATT | 35543479 | 35543783 |
|  | CTAGAGCAGAATGTGTAAAAGAGGAATCT | CAACTGATGCTTTTCTCTCTGTTCATCTA | 35554592 | 35554948 |
| FOS | GCAGGTCAGAAATGGTTTCACAG | TCTAGTTGGTCTGTCTCCTGTATACAG | 75747128 | 75747502 |
|  | GTGGCAGGATCGTTTCTCTTCA | GCTAGAGTTCCTCACCTGTTCCA | 75746472 | 75746846 |
|  | GCCCGTGACGTTTACACTCATT | GGTGAGTGGTAGTAAGAGAGGCTATC | 75745462 | 75745783 |
|  | CTGGTGCATTACAGAGAGGAGAAA | GGCTCAACATGCTACTAACTACCA | 75748178 | 75748405 |
|  | TGGAACCTGTCAAGAGCATCAG | CGAAGGAAGACGTGTAAGCAGT | 75747742 | 75748008 |
|  | TCCATGTACTGTAGTTTTTCTTCAACATCA | CTGGTCTCCAATAATACCTTTCAGAAACTT | 75748646 | 75748998 |
|  | CGTTGTGAAGACCATGACAGGA | CAGTGGCTTCATCCTCTGTACTG | 75746768 | 75746927 |
|  | TAACCGCCACGATGATGTTCTC | GGGAACCAATTCTTACTATGGCAAG | 75745675 | 75746025 |
|  | CCCTTACACAGGATGTCCATATTAGG | CGCAGCCACTGCTTTTATAACA | 75745201 | 75745516 |
|  | ACCTATCTGGGTCCTTCTATGCA | CCCTAGGTCTACAGGAACCCTCTA | 75747862 | 75748236 |
|  | GAGTGTGTATTGTTCCCAGTGACA | CCTCAACAATGCATGATCAGTAACATT | 75748347 | 75748713 |
|  | GCCCTTGAGTAAGACTGTGTCT | TGGGAACAGGAAGTCATCAAAGG | 75747435 | 75747809 |
| FYN | ACCAGTGAATTTAGCAAATGTTCTTTTAGC | CATGTTATTAGTGCTGGCTAGATGTAGAT | 112035428 | 112035767 |
|  | ACGCAGTCTTTATTTTCACCTTCCA | GGTGTAGTAGCACTGTTTGAGGA | 112021218 | 112021592 |
|  | CCAAACCAAGCTGAAGAATGACATT | GCAGGGTAACTTGATGTGCCAT | 112015447 | 112015821 |
|  | GACCCAGGCAGAAACTAGAAAAGTA | TAATCTTATGCAAATAGCTGGTGGTCAA | 112017373 | 112017746 |
|  | TGAAAACAGGACTCCACTCACAAG | AAATACATCCAGGGAAAGGTTAAGGAC | 112025062 | 112025436 |
|  | AGGTTTTCACCAGGTTGGTACTG | TTAGCCACATGCAGACGCAAAGAAG | 111982946 | 111983253 |
|  | GCTTACCTGTTCCTCCTCTCGTA | CTGTAGAGCAACCCAGCAACTAG | 112041002 | 112041330 |
|  | ACAGATGTGCCACAGAACTACG | CCTGTACTGCTAACGAATGACTTTCTAT | 111981474 | 111981848 |
|  | CCCAGAAATTTTAGTTGCATGAGAAAGT | CCCAAGCTGAATTTACAAGTTCTGT | 111981904 | 111982177 |
|  | CGCTCGCACAACAACCTC | ACAGTAACGCTCAACCCACTC | 112194458 | 112194827 |
|  | TGTCAGAAAACATCAATTCAATGTCACATT | GAGGAAACTGGTAGGTGGAATTTAGTAC | 112101610 | 112101984 |
|  | ACCCACATTGTATATGTACAGGGACATATA | CAAATCCGAACCTCCTCTGTGAA | 111982400 | 111982772 |
|  | TCTTCAAACTCAAGCTCAGCAGAA | CCCTCTAAACCTGCTCTCAGGAA | 111995576 | 111995944 |
|  | CCTACCATTAAGGGCTGGCAAA | TCAGAGTCAAGAAAATGAGGCCTAAAAA | 112023944 | 112024318 |
|  | CTGTGCCCGTCTAGTTATCCAG | GCAAGTCACCTTCCATCTTTCCT | 112020592 | 112020958 |
|  | GGCAGAAAGCCTGAAAAACTCA | GGAGTTCAGAGTAAAGGAATAGGTTGGA | 112028990 | 112029364 |
|  | ACTAGAATGTTTGCTGATCGCAGAT | CCCATGTGGATGGCATTATTCC | 112015658 | 112016030 |
|  | AGTAGTGCATAGTTATTCCACTGGAGAA | TCTAAAACCCATAGCATTGTAGTCATGG | 111981782 | 111981970 |
|  | GAGAACAAATCGCAGTAAGTTGAAGC | CAATCAGGACAGGTGTTTGTTTTTGTT | 111982116 | 111982487 |
|  | GCCTTTTACATAACATCGATACAATGCATT | GCTTCCTGGAAGACTACTTTACCG | 111982680 | 111983003 |
|  | TTCTTTCTATTTCACAAACCAGTTCCTTCT | GCAACTGTGACTTAGCAACAATTGT | 112167622 | 112167996 |
|  | CTTCCACACCAGTGGCCTATAT | CCTACTATATTGTAGACCTGTGCCAGAA | 112080100 | 112080467 |
|  | CAGGTGAGGCTGAGAGTCATTG | GGTGTGAACTCTTCGTCTCATACG | 112040708 | 112041059 |
| GABRA5 | CTCCAGCCCAGAGACGACATG | CCGTCGGTGAGGATGCATC | 27112223 | 27112505 |
|  | AAACAACTTTCTGGAAAAACAGGATACG | GCCTGCATAAAAGTTTGCCTATTCTATTTC | 27193585 | 27193958 |
|  | TTCCACAGATGCAGTGCTTAAGT | TCACCCTAATTCGTTGTTTTCCACT | 27113719 | 27114093 |
|  | CCCAGATATACACTGCGTTAGGT | GGCCCTGTTCTGCTGAAGTTT | 27111734 | 27112103 |
|  | GCACCCAGGCCTTTAGACATG | TGTGCTGGTGCTGATGTTCTC | 27182127 | 27182474 |
|  | CTCGTGCCTTCCTTTCCACTAG | TCAGACCTATGGTCAGAACAGCT | 27128462 | 27128813 |
|  | TGAATGCTTGTCCAAACCGACA | AACAAAGAGGCAGCACTAGAAGTT | 27184959 | 27185332 |
|  | CAGATCCTCGTCTCAGGAGACA | GAACAGCCATCATACCAAAATCCTG | 27125878 | 27126249 |
|  | TCTTTTGAGAGGTATACCTTTAGAACTCCA | CTTTTATCACCGGCTCCCTATTCAAATA | 27192992 | 27193360 |
|  | CTACCTGAACAATAGCCAAGAAAGGA | AGGAAATGAGTCCACCAAAACATGAA | 27194078 | 27194408 |
|  | TGTTCTGAAATTAGGAAAGTACTGCATGA | TGAGAATGTCATCAAAACACTGTTTGTTTT | 27193889 | 27194144 |
|  | GGAGAAGGAGGATGCATCCTC | CAGGGTAAAGTTTGCGCTCTG | 27112477 | 27112718 |
|  | CACCTACAACACAAACTTACGTAATTTGG | CACGCTTCTTAAACAGACTGCAT | 27159784 | 27160158 |
|  | CGCCGAAGATGCTGTTGAG | ATGCTGCCAGCGCGAACT | 27112054 | 27112419 |
|  | AGACTGAACCAGTACCACCTGAT | GAAGCTGTCTACCTTTCCTGCTTAG | 27182412 | 27182773 |
|  | GGGAGCAGCTCGATTTCATCTG | GCTTTGTCGGAAAAACACGTCT | 27128142 | 27128516 |
|  | TCTATCACTTTGGGCATGTTGTACTG | ATGTTTGGATAGAATCAGGCACACTT | 27188271 | 27188645 |
|  | CTTGTTCGGCACTTTCAACTTAGTTT | CAACGTATGATACTGGGCATCTGA | 27193296 | 27193647 |
|  | CGGGCTCCTTTCTTTGCAAATT | CGAGGACTTGCTCAATGGATGT | 27114213 | 27114577 |
| GALR2 | GCAGAGTCGCACTAGGAGTT | CCGTCCAGGGTGTAGATGGT | 74070860 | 74071233 |
|  | TTTACGCGCTGGTCTCCAA | ACAATGACTCCAACTCTGTGACATC | 74073220 | 74073559 |
|  | CTCCTCTGTGTGCGGTGTAA | ACAGGAAGCAGGTAGCTGAAGA | 74072582 | 74072947 |
|  | TGATGTGGCCTGAAAGCACTTAG | GCTTCCTCCCAGAATCTCAAGA | 74073500 | 74073669 |
|  | GAGCTCGGAAGCAGGTACAA | GCCCGAGACGTTCATGGT | 74070661 | 74070979 |
|  | CTACCAACCTGTTCATCCTTAACCT | CACTGCCTTCTTAGTGTCCTGT | 74071140 | 74071451 |
|  | GCCATGGACATCTGCACCTT | AGATCGTGCGGAAGCCTTT | 74072904 | 74073267 |
| GCH1 | CTGCGCCAAAAGTGAGGCAACT | GATAACGAGCTGAACCTCCCTAAC | 55368931 | 55369195 |
|  | GCTTTAGGCTCAGGGATGGAAA | AAAGCAGTCTGGTAAAAGCTGGT | 55312343 | 55312715 |
|  | CTGTGTTTCAGACCCTCCAAGT | GTCTTAACAGTTCCAGATGTTTTCAAGG | 55326245 | 55326603 |
|  | GATGTCTTTGCTGAAACAGAATACAGC | CGTTGTTCTAACATTTCCTAGAAGTGTTCT | 55308643 | 55309016 |
|  | CGAGGTCTGCGGCTAAACTC | GCTCATTCCGCAATAAGTGGAG | 55369428 | 55369790 |
|  | TGAGTTGAGTGCTCAGAATTCTACCTA | CACAGAAACACAAACACAGTTATTCCAT | 55309121 | 55309467 |
|  | CGTCAGTTCATTCTGTGCTCGTT | GTTTACGAGTGTATTCTCGTGTCAACATA | 55309720 | 55310076 |
|  | ACTTTCGGCACTACACCACTTTTATT | TCTGTCCGGTCTTGTTTGTACATT | 55310312 | 55310678 |
|  | AAATACCTGAGATATCAGCAATTGGCA | TTTTAGGGAGGATTAACGTTCGTTTATGT | 55331934 | 55332263 |
|  | CGAGCTCAGGATGGACGAGTA | GCGTACCTTCCTCAGGTGACT | 55369139 | 55369501 |
|  | TGACAAGGAATAAAGTTCACATCTGTAACA | CCAGCAGCTGTCTACTCCTTTAAA | 55310614 | 55310910 |
|  | CTAATAAGACAACAGCCTGTGGATACATT | GCCCAGTATAAACATTAGCCTGCTTAATAT | 55308947 | 55309188 |
|  | GAATTTGAAGAGCACTATGTCAACCAAA | CAGGTCTGCAGAACCATAGCTT | 55309401 | 55309775 |
|  | ATTCTTATCAAGGCACAGAGAGTTAAATGT | GGAACTGTGCCCAGTATACTGTTTTT | 55310007 | 55310374 |
|  | AAGGCATGGTGATGCACTCTTATAA | CATCTCCCTGTGGTACTTGTATCTTTC | 55313636 | 55314010 |
| GDNF | GGGAGGGAACGGTTCTTACA | CCCAGGCTTAACGTGCATTC | 37834729 | 37834981 |
|  | ATATTTTGTCGTACGTTGTCTCAGCT | AATGATCATTTTGTCTCATGTGCCATTTT | 37815911 | 37816285 |
|  | TTCTCTCTCTCCTGTCCAGTTGT | CAGAAATAGAAGGCTGGTGAGTGA | 37815523 | 37815897 |
|  | GGTGGAGCAACTTGCTTCCATTA | CCCTCTCACACTGTCTTGGGAT | 37812983 | 37813288 |
|  | GGGAAGGCCTTTCCTCCATTTC | GTTGGAGCAGGAGGAAGCATAG | 37813286 | 37813659 |
|  | GGGTACCGCACTGTCACTTA | CGTGCTGCCTTTAAATGAAGGT | 37813692 | 37813991 |
|  | GCTCCTAAGTATGAGGCTCCATGT | AGTTCCTTTTAGTAGGTAGTCCTGGAT | 37813991 | 37814337 |
|  | AACTGAACCTTTTCTACATATGGTTATGCA | AGTATATGTGCTCGCAAAATGCAAAG | 37814334 | 37814708 |
|  | ACTTTGGCACCTTTGAAAAGAAACTTTAAT | ACCAAAAAGGCTGTGCTCATGA | 37814706 | 37815080 |
|  | CCGTGATGGAAATCAATCACCAAC | ACTAGAAGCTCAGGGCTGATGT | 37815110 | 37815484 |
|  | CTAAAGTGTGGAGAGTCTAAGTCTTGG | AGATGGAAACTGAAGTTTAAGAATGCCT | 37823918 | 37824292 |
|  | AGTTATGAACATTAAATCACGCATGACAAG | GGTGTGAGGATTGAGAAGCCAA | 37835665 | 37836035 |
|  | GGGCAAGAGTTCGCAATCC | CCGGTTGACGTGGTGTCTC | 37839726 | 37840098 |
|  | CCACGTGGAAAAGGCAACATG | CCCAGGATTGCGAACTCTTG | 37839380 | 37839748 |
|  | GGGTGATTCCTAAGAAATACTTGTGGATG | CATCACCTACACACCACAAGCA | 37812706 | 37813032 |
|  | GCACTGCCAAGGTTCTCTGAAT | CAATTAAATGAAATACTGGCCTCCATCA | 37813147 | 37813453 |
|  | CTAGTAGCCACTAAGGTAGGTAGTAATAGA | CAGAAACTTTAAAAATTAGGCAGGCATGAT | 37813476 | 37813846 |
|  | GCAGCACGCTCCTAAGTATGA | CGACAATGCTGTCAGGTAGCT | 37813984 | 37814286 |
|  | ATTGTCGACATTACTTTTAGGTCAGCA | CAAGGGTCCATGCTTTTCAAAGT | 37814280 | 37814651 |
|  | GACCCTTGTTCCCAATTCAAATTTCC | GGTTTTCGCCAGCATTCTTGTA | 37814644 | 37815001 |
|  | CGAAAACCACAACTCGTTTCCT | GTGTCCGAAGACACCAGGGAAA | 37814994 | 37815345 |
|  | GGACACATGAAAACAAATCACAGGA | GGACCAAGGTTCCCAGGAAATG | 37815340 | 37815699 |
|  | CTTGGTCCCTTTCTTTGCACTG | GGGTCTGGGCTATGAAACCAAG | 37815692 | 37815999 |
|  | CCAGACCCAAGTCAGTGACATT | GCTTAATCGGCTGATAGTTTTGCTG | 37815992 | 37816365 |
|  | ACCAGCGCGTGTCACTTT | CCGGACGGGACTTTAAGATGA | 37834541 | 37834915 |
|  | CTTCTTGTCCCGGTAGCTCCTATA | TCATAACTTTAAGAGGTGGGAGGAGTATT | 37835341 | 37835672 |
| GFRA1 | GCATTATGCCTCTTCATTATCATCATCCTA | TTTGAAATCTTTCAAAGTTTCAGCGTCTT | 118028857 | 118029231 |
|  | CTCCTTCAGGCACTGATCACTG | GCTCTGGAGCAAACCCTTGAAG | 118030551 | 118030888 |
|  | GTTCAGCTCCATCCAGTGAAAGA | CTCGGGTTGAACCCAACAGACA | 118031662 | 118032036 |
|  | CGGATGCTCGTTAGGGATGTTA | GCCCTAAGAAGAAAGAGCCCTT | 118032897 | 118033269 |
|  | ACAGGACGATGGACAAATGTGT | TGAGAAGCTGTGTGAGATTTAGGC | 117816714 | 117817061 |
|  | GCTTCTCAGCAGGAGTTGGATT | CTCGGATGTGGAGAGGAGAATG | 117817054 | 117817392 |
|  | CATCCGAGAAATGCCTTCAGCTA | GGATGTCCTGAGTATAGGGAACCA | 117817385 | 117817712 |
|  | GGACATCCACTGGATTTTCTAATTCTTCT | AGCTCTTCCATGATAGAGAAGGCA | 117817705 | 117818045 |
|  | GAAGAGCTGGATTTGCCATTGT | ACAGGATGCTGACAAGAAAGGT | 117818038 | 117818389 |
|  | CATCCTGTGCTTGAGGCAGATTA | GCATGACCCATAGTAGGTACTCAGA | 117818382 | 117818749 |
|  | GGTCATGCTTTCTGCTGAAGAAG | GAGAACCACAGCCTGCTAATAGT | 117818742 | 117819097 |
|  | GCTGGAGGAACTTCGTATTTGGG | GGGCTGGACCTCTGAAAAAGAT | 117819327 | 117819666 |
|  | CCCACAAAGAATTCTAGACAACAGAC | GGCAAAATCAAGGTGCAACAGT | 117819666 | 117820037 |
|  | TTTTGCCTTATCTCGCCAACTCT | TCAGAGGAGCTGTCTTTTGGAAAAT | 117820031 | 117820376 |
|  | TCCTCTGAGGCCACAATTTTGT | GAAGTTGTGTCTTCGAGTTCCTCA | 117820369 | 117820724 |
|  | CAACTTCTAAGACATTCTTACTACAGCACA | CCATGCCCTACTTATTTCAGAGAGA | 117820718 | 117821091 |
|  | GGGCATGGCTAGATGTAATCAGG | GGGACAACACGTTAGAGAGATTCTT | 117821084 | 117821438 |
|  | GTTGTCCCTGAACAAGATGCTTC | CGCATATCTCTTACAGAGTGTATTTTGTTT | 117821431 | 117821804 |
|  | GATATGCGCTCATAGATAACTGGCA | GGAAACCAGGGTGCACATTTCT | 117821797 | 117822139 |
|  | TGGTTTCCTCCTCCATCTCTTCTAC | GTGACAGACTGCAGACCATGTT | 117822132 | 117822489 |
|  | TCTGTCACGGAGGACATGAAAC | CTGTGGACAGGCAGGAGGAAACA | 117822482 | 117822853 |
|  | GTCCACAGTCACATGATGCAGA | CACTCTCCTAATCCTAAGCCTTACCT | 117822846 | 117823183 |
|  | GGAGAGTGTCGTTCAGGGATTG | TTCTGGCCTTTCCTAGCTAGAGA | 117823176 | 117823534 |
|  | GGCCAGAAGTAAAACTGTTAAAATCATCAT | AGGAGCTCAGTTGAGAAACAGTTC | 117823527 | 117823818 |
|  | AACTTTGTGAACTGGAATTCAAAGTCTTC | CAATTATATATGGAGCTCACAGCTGTGT | 117824897 | 117825271 |
|  | GTGACTAAGCAGAAAGGGAGAAAGACAGAC | TTGGTTTCTTTTTAGGCCATGGAAAAG | 117853043 | 117853417 |
|  | GACCCTCTCACCTGAGAGGTAT | GGGAGAAGCCCAACTGTTTGAA | 117884434 | 117884789 |
|  | CTTCTCCCTCTCTTCATAGGAGCA | TAAATGGCAGGATCTCAATTAAAACAGAGT | 117884782 | 117885155 |
|  | GAGCAGCATTCGTCATCTCTCT | TGTGCCTCTCTGGCTGTGTATA | 117849141 | 117849515 |
|  | TACAGGCATGTCCTCAAGGATTTAAC | GCGTGTGCAGAAAAAGGCATAT | 117856013 | 117856387 |
|  | CCTAGGAAAGACTCAGAGGGTAAGA | GCTCTCCGCTCTCATCTCAAAG | 118031452 | 118031826 |
|  | GCAATGGGTTCCCAAACAGAAC | GCCGCTTCCAATAACCACTAAC | 118032628 | 118032936 |
|  | GGGTTTTCCGGTGAATGGGATAAAA | TACTGAGCTTCCCACTGGTAGAA | 117816392 | 117816762 |
|  | GCTCAGTAAACACACAATATAACATGGAGA | GTTTTCCCATCTTGCGTGCATT | 117816755 | 117817129 |
|  | GGGAAAACTTTAAAGCAAACTCTGCA | TGGAGGGCTATATGATCCAGCA | 117817122 | 117817496 |
|  | GCCCTCCAATCATTGTGTGTGT | CAGAGCTGCTTTAGCTGAGGAA | 117817489 | 117817859 |
|  | GGTACCTGACAGCTGTGACTTC | GTACTCTGACGGAATAAACCAGCA | 117817890 | 117818261 |
|  | GTACTCACTCAGTAAACGTGCTGA | AGCTGTATTGACCCTGAATGTGTC | 117818258 | 117818632 |
|  | TACAGCTCAATGCCAGGAATACG | GCAGGTGCCAATATCAGCATCT | 117818626 | 117818999 |
|  | GCTGGTCAAATGAGCTTGGAGA | TCTCTCAGGAGCCAGATGATCTG | 117819029 | 117819387 |
|  | CTGAGAGAGGCTTCTCTGTCTTAAAATAT | GTGTATATCTGACACCTCTTGGCTT | 117819380 | 117819754 |
|  | GCTTCTAAGGGTATCCAACCAAATCC | AGACTATGTGACACCCATCTTTTCAAG | 117819791 | 117820165 |
|  | TTATCAAAGAACAGAGGCAAGAGCAA | GCACTCCATCTGCTATACTAGGAGA | 117820174 | 117820548 |
|  | GTCTGATTTAGAGACAAGAAGGCAAGA | AGAGAAGGAATTGAGAATGCCAATTACA | 117820557 | 117820930 |
|  | CCTTCTCTATATTGTGTGCTTCTTCCTTC | CTTCTTGGCGTGCTTAAAAGCA | 117820923 | 117821287 |
|  | CCAAGAAGAAGTGAGACAGGTAAATGATTT | ATGACCAAAGCGGAAATGGGAGT | 117821280 | 117821654 |
|  | TTGGTCATCTGATGCTAATTAGAGCTG | TTGTGTCCAAATGTTAATCAACACAAATG | 117821647 | 117821997 |
|  | ACTTTTAACACCAATAGAGCTGAAAGACTA | TGATAAGGCATCTGGCTTTGCT | 117822002 | 117822376 |
|  | CCTTATCACCAGGGTCCTGTTCT | GCAGCATCTCTTGACACACTTG | 117822369 | 117822743 |
|  | GATGCTGCAGAACATTTACAAGCA | CGCTCTAATACTCTTTACACATATGAGGTT | 117822736 | 117823110 |
|  | TTAGAGCGACATTTCAGCTATATTGGAC | GGCTTTGTGGAAACAGCTGGTA | 117823103 | 117823387 |
|  | ACAAAGCCCACTCTTGGTAAGAATC | GGGCTTCTGTGAAAAACCTGATG | 117823380 | 117823737 |
|  | AGGATCACAAGAAGCTTTCTTAAAAGGAAA | GTATACTGTGGTAGAATGAAGACATGCA | 117823741 | 117824115 |
|  | GCCCACTTACCTGCAGATGTAATTC | CCATGCCTGTCTTTCTCTGCATT | 117884722 | 117885096 |
|  | GGGAGGCTGATGGCACTTTAAG | TTGAAATGCAAGCAGCCTCATTC | 117970977 | 117971351 |
|  | GGGAGAAGTGAGTGGAGGATGA | CGCCTGGATTGCGTGAAA | 118030233 | 118030592 |
| GPR132 | GGCGAAGCAGACTAGGAAGATG | GGTGTTCCAGACGGAAGACAAG | 105517706 | 105517946 |
|  | TTCTCCGCCAGACAAGCAGG | TAGGGCAGCCTACCACCTCC | 105516496 | 105516835 |
|  | GAGTGGCTTCAGGAACGAGAAA | CCTTGCAGACCACTACACCTTC | 105517192 | 105517418 |
|  | CCTGGTGACGTCTGTCTTCATG | CACCTGGTTCTCCTCGTCAAAG | 105517463 | 105517699 |
|  | ATGCAGCACAGGAAGAGGATGCT | GCAACAACGTGTCCTTCGAAGA | 105518067 | 105518376 |
|  | TGAATTATGAGACAACATGCTTCATGGA | GGAGCCTTGCTTGTGTTTCTCA | 105523422 | 105523796 |
|  | CCAGATTAAGAACCCTCAACAAAGC | GCCATGCTGTCATCACCAGTAA | 105531559 | 105531890 |
|  | GCCCTGCCTTGTGCTTTTATTTC | GGCAGCTATGCAGTTGCTCTT | 105515889 | 105516186 |
|  | TAGCTGCCTGACAAAACGAGAG | GCCTAGACCAGGTCACACTGAT | 105516179 | 105516438 |
|  | GCCGTTCATTCCTTCAATCCCAA | GCCCAATGCTACTGAAAAACGG | 105521396 | 105521753 |
|  | CATTGGGCACATTCACATTCTTCTG | ACCCTGGAGGACACACTGAATA | 105521746 | 105522085 |
|  | TCCAGGGTCTCGCCAAAAATATAAAT | GTCTGACCCAGGAGAACGGAAA | 105522078 | 105522313 |
|  | CTCAGCAGGACTCCTCAATCAG | GGTGGAAAGAGTGGTCCATGAA | 105517330 | 105517500 |
|  | ACTCCTCTCAAGGCAGGAGAAAT | CCCTTGAGAGTCATCAGAAAAATACA | 105515637 | 105515954 |
|  | AGCATGGTGCTCACTGACATT | CCCTCTGGAAGACAAACTACCAA | 105516874 | 105517236 |
|  | CCCTTATGGATTCTGGACACTTCT | TCAAGCAGAGCATGGGCTTAAG | 105517500 | 105517797 |
|  | CTGCTTGATGCTCCTGAAAATCC | GGTGACCGCCTACATCTTCTTC | 105517790 | 105518126 |
|  | GGTGCTGGTTGCGGATATAGAT | CACAAAGCTTCTATCCTTTCTTCCCA | 105518155 | 105518529 |
|  | CTGCAGTCCTAGCTGAGGTTTC | GCAGAGGAGCTGGATTTTGTCT | 105531754 | 105532117 |
|  | CACCTGGCCTAGGTACTCTTC | TGTGCACCATCATGCCTGTTTATA | 105524317 | 105524571 |
|  | CAAAAACAAGGAAGAGCAACTGCATA | CTGGGTCACCATCGAGATCAAT | 105516155 | 105516369 |
|  | CCAGTGAGGTAGGTGTCATTGG | ATGTCCACGCCCAGAACAAC | 105516366 | 105516685 |
|  | AAAAATTGAAAAAGTGGAAAATTGTGGCA | CCTTTGAAAGGAAGGGAAATCCTG | 105521671 | 105522045 |
|  | GCGGGATGGTATTCCATTGTATTC | GTCATTCTCAGCAGCTCAGCTA | 105522045 | 105522253 |
|  | AGAATGACGGTGTCAAGAACATGA | CGAAGTGGTGACAACTGAGTGT | 105522246 | 105522564 |
| GRIN1 | CTCCTCGAGAAGGAGAACATCAC | CGCCCATCTCTGGAGTGA | 140051404 | 140051562 |
|  | GCTTCATGGAAGACCTGGACAA | GCAATCTCGATGAAAATCAGGAAGATC | 140058025 | 140058274 |
|  | CCTGAGTCTGGGTCCACTTCA | GGTCAAACTGCAGCACCTTCT | 140043136 | 140043485 |
|  | CATGCAAGGAGGAGTTCACAGT | ATGCAAAAGCCGTAGCAACACT | 140055566 | 140055780 |
|  | GGTGGCCGTGATGCTGTA | CGCGCTGAGAAGCTTCTG | 140056707 | 140057067 |
|  | CCTCGCAGGTGAACAACAG | ACGGCTGCATGAACGAGT | 140056368 | 140056663 |
|  | GCAGGCTTCGCTCTAGGAG | CCGACTCGTTCTTGCCGTT | 140050993 | 140051356 |
|  | CCGGCTTTGCCATGATCATC | GCACTCTCGTAGTTGTGCTTCT | 140057088 | 140057425 |
|  | ATCCAGGCCGTGAGAGACAAGT | GACACTCACTTGAGGATGGACA | 140057436 | 140057791 |
|  | GGGCTGCGAGCTTATTCAGA | TCTCCCTATGACGGGAACACA | 140061671 | 140061974 |
|  | GGAAGTTCGCCAACTACAGCAT | CCCACTTCCTTTAGTGGCTTTCA | 140052899 | 140053270 |
|  | GGCTCGGGACTGTCTTCAAC | GCCCTCCCTTTACACTGTCTG | 140062950 | 140063260 |
|  | CAGCAGTACCATCCCACTGATAT | CAGGACCCATCAGTGTCCTTG | 140062223 | 140062352 |
|  | TGCTTCCAGATCTCAGCCTCTA | TGGTGTGTGAGTTCCATGTGAG | 140036388 | 140036704 |
|  | GAGGAGGTGGTGTGATTGCTTTA | TGGTCGAGGTTTTCATAGTTCCTTTTT | 140042509 | 140042635 |
|  | GCAACTCCTCCAGTCCTCAGA | GCTCTACCACTCTTTCTATCCTAAAATGAA | 140059302 | 140059657 |
|  | CCGAAATATCGCTGACTGTGG | CTGCAGCAGCTGCAGAAACG | 140033235 | 140033609 |
|  | CGCAGAGCATCCACCTGAG | GTGTCACACACGGAGCCACA | 140040173 | 140040439 |
|  | GCCTCCTCCAGACTCGAGAG | TGCTGGTTCTGTACAAGGTGG | 140062861 | 140063065 |
|  | GTGACGACTGGAGAGCTGTTTT | GTCTTCCATGAAGCCATTCTCGT | 140057688 | 140058038 |
|  | CCCAAGATCGTCAACATTGGC | GGAAAGGATGGAATGAAACTGGG | 140034008 | 140034278 |
|  | GCGACGCGCTATTGAGAG | CCGAGGGATCTGAGAGGTTGA | 140061916 | 140062277 |
|  | CGAGCTGGGTAGGGTCTTG | GCCCATCATCCCATTCCACT | 140056252 | 140056417 |
|  | GTGGAGCTGAGCACCATGTA | CAGATGAAGGCATGCAGCTT | 140057373 | 140057641 |
|  | GCCCTCTAGGGTCTGACAGA | CCGTGGCGTAGATAAACTTGTCC | 140057004 | 140057334 |
|  | CCGGTGCGTTCTCCTTCA | GGCTCCTAGCTCCAGTCCT | 140058118 | 140058477 |
|  | GAGGACGTGTCCTGAACACTT | GGTGCAGATCACCTTCTTGACTG | 140055313 | 140055621 |
|  | CAGGGCCTGACTATTCTGGTC | TCCTCGCTGTTCACCTTGAAC | 140056514 | 140056888 |
|  | GGACGATGCTGCCACTGTATAC | GGTCTTCCAGATGTTGGTGTTG | 140051124 | 140051472 |
|  | CCACCACCCTAGCCATCTAATC | GAAAGGTCCAGAGAGTGAGAGAGA | 140059595 | 140059929 |
|  | CCTGCTAACACTCTTGCTCACA | TACTTGGCTCAGGTACCATCCT | 140043433 | 140043616 |
|  | GTCTGCATATTTTCTCTGTGCACATTATT | CGGAGATTCGCATGCACTTC | 140042569 | 140042938 |
|  | CAGGAGAAGGAGCATCTCTGAGA | GATGATCTTCCTGTCATTAGGGATGAC | 140052782 | 140053099 |
| GRIN2A | ACCCAGCATCACCGCAATATTT | CAAGTTGGTAATCCCTTTCTTCTGGT | 10274146 | 10274356 |
|  | CACTTCTGTTGCCTCAGGAGTT | ATCAACTGCAGCAGTTTATATCTGTCA | 9847350 | 9847612 |
|  | CCCTCTGGGACGTACCTGTA | GGCATCGGGCTTCAGCTT | 10275719 | 10276075 |
|  | CCCTCTGCATATGGAGTTCAATCC | CTGCCAAAACAAATTTAAAAGCATAAACCT | 9847745 | 9848119 |
|  | GGAACATGAATGTTAAAAGTTCCACTTTCA | GAAGATCCAGAAATACGAAAACATTGTCTT | 9848322 | 9848680 |
|  | TGAAAGAGAATTAACTGGAAGAGGTCATG | AGCTATGCAGAAATGGAGCACT | 9848896 | 9849204 |
|  | GCTAGTGTCCTCATCCATCAAATGG | AGAGCTTTGGTAGTCAGAATGATTGAG | 9849428 | 9849797 |
|  | TACCCACACACATGAGAGCATG | TCCTGGGCATATACCCTTGAGT | 9850000 | 9850327 |
|  | TGATATTTTCAAATTCAGGTGCAGATCCA | AATTCTTTATCAAGTTCCAAGTGCCTACT | 9850580 | 9850819 |
|  | GAGGCACATGTCATAATGCAAGG | CAATGCAAACAGAGACTAAACAGCAA | 9850981 | 9851328 |
|  | CCCACTTCACATCAAGACAGATTCT | CGTGGTAGCTCTGCTGATGAAC | 10273748 | 10274065 |
|  | TCTTGGCTGTGAGATGGGATCT | AGGCTTCTTGTGATGAATATAGCATTGT | 9984716 | 9985089 |
|  | TAAGGAAATCACACACAAGTCCATATCG | TGAATACAACTGCTTGTGTACACATGTAT | 9852141 | 9852513 |
|  | CATGTGCGTGCTATTACCCATG | GTCAGTTCAGTCTTTGCAAAAAGAATATCA | 9853803 | 9854167 |
|  | GGCCACGTTTTCAGTTTCTTTATCC | AGCCTTTTAGCCCTTTCTACCTTC | 9854414 | 9854762 |
|  | GACCCTGAAATTTATTGCAACCACT | GTATTTGGGATAACTAGCCATCAGTGT | 9854993 | 9855341 |
|  | CATTTCATGTCATATATGCACAGGTTTGAG | CTGAGTATCCTCCAAACTGTGGATAC | 9855591 | 9855792 |
|  | GAAACGGAACGAGTTATGGTTACTCT | CGTGATGACTGTGCTACCTTCATTAT | 9856040 | 9856386 |
|  | CAGAAATACAATGGGAGCAGATAGGA | TCCTAACACCAGACAAAATGATCACTAATT | 9916016 | 9916390 |
|  | GGGAACCCTTGTCTTTCAAGGTG | AGGAGCCTGGTTTCCACTTAGA | 9857806 | 9858030 |
|  | GCCCGATTTGACGTTTCTGAAATG | GGGAAAGAGAGCATTTTTGGAGACA | 9858198 | 9858550 |
|  | GACGACCGAAGATAGCTGTCATT | GGATTAGCCGTCAGCATTCCTA | 9857190 | 9857547 |
|  | CCCAGTCATCGTAGGAGACAGA | CTGTCATCTTGCTCTACTGTTCCA | 10031970 | 10032149 |
|  | CTCCAGGGTGCACAGGAGAAT | CTGGCTCGTCACAAACTCAG | 10276130 | 10276332 |
|  | AAGAGAGAGAGAGAACAACAGTACTTTACT | ACTCATCTGAAAAATAGGAGAAGAGTACAG | 9934689 | 9935054 |
|  | GCTTTTCTAAACCTGCTTGCAGT | TCGTCTGTTCCAAACCCAGAAG | 9862656 | 9863028 |
|  | CACCATGCCTGGTCTAGAGTAATG | AAATATTTTTGGCGAGCCTGCAAA | 9927860 | 9928189 |
|  | GCACTTTCAACCCTGTTCACCA | CTCTAAATACCTTACAGAGGAGCTGGATAT | 9852666 | 9853010 |
|  | AGTGGTCGACATAGCAAATAGAATAAACA | ACTCATTACTGTTATGTCCTCTGCAAATC | 9853246 | 9853592 |
|  | AAATTACATGGTGGGCTGACCTTAAT | GGAGGGTAAATGTTGGATGTCCAATA | 9856630 | 9856950 |
|  | ACTGCCAGCCTTTTATCTTCAGT | GTATCAGAGCGAGGATTAGATCCAAG | 9851550 | 9851903 |
|  | GCTGGCTGAGTTTGTGACGA | GAAGCGGAGCTAGGGATCTT | 10276308 | 10276658 |
|  | GGAAGTTGAGGCGAACTCCAA | ATGCGGGAACCCGCTAAAC | 10275518 | 10275829 |
|  | TCTGCAGCAGGGCTCTAAC | GAGGTGCCCAGTTAGCTTCTC | 10275911 | 10276195 |
|  | ATCATGCAAAGATCCACTGGGAA | AGTGTGGGATGCTTTCAGGATC | 9892040 | 9892414 |
|  | CACATATTGTTACTATCGATGATCCATGCT | GGAAAGGATTTGCCTCTCCAGAA | 9943535 | 9943889 |
|  | CACTTATTAGCACTTCCTCATGCAGT | CAGGTTTATGTCTGAGTCCATTTTTAACC | 9847549 | 9847808 |
|  | GGTGATATAAACTATGGTGAATTCTGCCA | CCAGGTGATCTTGTTTGAAATTTTTGATGA | 9848050 | 9848392 |
|  | CTTCGATTGAATTCAAAAGGCTATGATTGT | GCATTTTTGCAATCCTAGAACATTCTTCAT | 9848605 | 9848965 |
|  | CCCAGAGAAGAAATGCTATTGGTCAAA | CCTGGCATTTATATTTACTGAGAGGTGA | 9849145 | 9849494 |
|  | ACCAAGAAGGAAAACAACAACAACATTTT | TCAACATCAGGGCTAATTCCATTAAACATA | 9849731 | 9850062 |
|  | ACCCTAACAGACTTCCCTGTCA | GGAATGTTACATTTTCTGCTTATCGCA | 9850273 | 9850646 |
|  | CTCTAGGGAACCTGATGCTTTCTG | TCTCCAGTCCTTGCCAAATCATG | 9850756 | 9851037 |
|  | CCTGAATCTCTTCCTCCTGAAAGGA | GCGTGGTTGTCATACACAAACA | 9923205 | 9923579 |
|  | ACGAAGGTGTGGGAGGAGATAA | ACTATGGGCAGAGTGGGCTATT | 10273904 | 10274271 |
|  | GCCAAACAGAGCTAAACAAGTTCC | CCTAGGCTCTTACTCAGACAAAGG | 9934399 | 9934773 |
|  | TCTAGCTCCCTAGGTTTGTCGA | TTCCAACAACGACCAGTATAAACTCTAC | 9857493 | 9857867 |
|  | GGCAGGGTCACATTTTCAACAA | GCCCTAGGTATCTTCCAGAAGAGATG | 9857976 | 9858258 |
|  | TTTTTCCTGGCTACAACTTTAGTTGTC | TCCTTAGGAAAAATCAACAGGGATATTGTT | 9851265 | 9851613 |
|  | GGTCAGAGAATCGAGCCAGAAA | TAGAATATATTCTGCTCGCATTTGCTTCT | 9851845 | 9852208 |
|  | CACTTACGTGTGAATTGTAGAAATCAAGC | GGAGACAAAATTAGACCTTTCCAGATACC | 9852445 | 9852727 |
|  | CCAGTCATGGTGCTGGCTAAAA | TAGGTACACTTGAATGCAAACGGTT | 9853531 | 9853860 |
|  | GTGGGTTCCATTTACCACTGGA | CAGCAGCATTATTTCTGCTATTAACCC | 9854105 | 9854476 |
|  | GGTGGATTAAGGTCTGGAATGCA | GAAAGTACACCTGACCTACAGTGTTC | 9854705 | 9855054 |
|  | GTAATGGCCCAAAACAGACTGAG | TTCTGAGTCGCCTGCATTATCC | 9855281 | 9855655 |
|  | AACTCAGAAGGGAGAAATTGCTTGT | TGACTCAGGGTTGTATAGTATCTGTTACC | 9855731 | 9856105 |
|  | CCTTTGGCTCCAGATGTGTCTT | ATGCCAATGGTGATGTTATGATTTCCTATA | 9856328 | 9856696 |
|  | GATGAAAGAAAGAGAGAGGAGAGCAAAATA | AGCTCATCCCAAAAGAGTTTCCAT | 10031654 | 10032026 |
|  | GGGCCTCACTCAGAATGAGAAC | TCTATATAAACCACCTCCTGCCTCAT | 10032093 | 10032463 |
|  | GGTTGGCCACAAATGTTTGGAG | CAAAGTGGTTTCATGTTCACTGCT | 9858493 | 9858866 |
|  | ACAAAGAAACAGTACAGGAATGTGCATAA | AATTAGTCAAAAGATCCACAGCTGTCATA | 9852941 | 9853314 |
|  | AAAATACCTCCCTACATCTTCTTCCTCT | CCTTACAAACACTCGTTGCCAT | 9856886 | 9857245 |
|  | TGCAGAACAATGAGATAAATGAACCTCT | GGGATTGAAATTCGTGTGCAAACT | 9847120 | 9847406 |
| GRIN2B | GCTGAGTTGAGCTCCTAGCAAA | CTATACAAAGAAAACCACTGATAGGCTGT | 13767924 | 13768296 |
|  | GGCTTCTTCCTTGGTACAGTAAAGA | CAAAATCTCCATTACCATCTTTGGTTTGAT | 13906550 | 13906919 |
|  | GCACAGGTTAAAGAAATGGAAGAAAAGG | TTCTGGAACCCTACTGCTTCATATTAATG | 13722669 | 13723043 |
|  | GTACATGAGAACTTTGAGTATGGCTGA | TGTGTATTTGTAGGTTTCAACAGAACCA | 13761464 | 13761838 |
|  | CCAAGTGCTAACAGGTGCATCA | AATACTGGGACAGGCTGTTTGT | 13769320 | 13769660 |
|  | CTGAGGACTTGTTGTTGGCAAAG | GCAAGAAAGCAGGCAACCTGTA | 13716106 | 13716438 |
|  | GGAGTCGCGCTTAAAGTCATCA | GCTCCGATGTCTCTGACATCTC | 13716674 | 13717002 |
|  | GCCAGTCGAGAGTCTAAATGCT | CAACCTTGACTCTTTCTATTGTTTCTTTCA | 13714556 | 13714930 |
|  | CTCTGCTTGGCACATGATTCCTA | CTCCTTTTCACAGTTCTCTCCTTCTTC | 13715172 | 13715529 |
|  | TCTCTGTTCCCTCACTCAGACAT | GTCACGCAAAACCCTTTCATCC | 13715702 | 13716028 |
|  | TGAGGATCAAATGAAACCAAACATGC | TTGTTACAACACCCACGAGAAGA | 13905992 | 13906301 |
|  | CTTCCGGTCAGACATGAGATCAC | GACAGGTTACGTGATGTAGATCCTATTTT | 14018864 | 14019215 |
|  | AGGTTCCCGAACGTTCTCTCTA | GTCATTTCTAGCCTCTCTGGAGATCTA | 13717254 | 13717625 |
|  | CACCTGAGGGTTCCTTTTCAGA | GAAGGCAGACAACACATTGTGG | 13764534 | 13764907 |
|  | CCACACTCACCTATTTAGCATATTGGA | CAGAATCAGCTCAAGAAACTTCAAAGC | 13906241 | 13906612 |
|  | GCTCCACAGGTCTAGGGACAAA | GCGAGTTCAAGAAGTACCAAATTTGATAAT | 13828570 | 13828944 |
|  | ATGGTATACCTAGTGCATGTGATTCAATAG | GTCCCTTCCATTATAAATCCACTAATGCTA | 13724650 | 13725024 |
|  | CTGAAATGCATAAAGTGAGCACGTT | CTGCTTTGGGATTGGAGGTTTTT | 13768333 | 13768701 |
|  | GGGTTGGACTGGTTCCCTATAC | CCCACATGTTTGAGATGTCAGCT | 13715811 | 13716162 |
|  | CAGGGAGTTGTCCTCACTGATG | ACGTAGACCTGACCGACATCTAC | 13716392 | 13716729 |
|  | CCCTCGATGTTCCCATAGGTGA | GTGAGGAGAACCTCTTCAGTGACTA | 13716948 | 13717311 |
|  | TGTGACCTTTTCTTCCAAAAAGATCCA | CGGAATAATTACTCTGGATTCTGCATTGT | 14132756 | 14133130 |
|  | CCGTCTCCCATTTCCAAGTCTC | ACACTTGGGAGCAAAAGATTCTACA | 13714282 | 13714613 |
|  | TGTCTGTATATAAGCCAAACAATCACACTG | TCTGGCTAAATTCATGCTGCTTAGA | 13714860 | 13715230 |
|  | GCATCATCTCATGGGAACAGGAATG | CAATGGGCATGTTTATGAGAAACTTTCTAG | 13715464 | 13715765 |
|  | GCTTTGCACAGTGCTAGGCTAA | CTTTTCTCTGCTCCTTTCCTGTCT | 13719894 | 13720260 |
|  | AAAAGGATTAATTGCTGGCCTATCCA | ATGAGACCGACCCAAAGAGCATCAT | 14018586 | 14018922 |
| GRM5 | CTGAGGCTGACCGAGAAACACA | GATTTTGAGCTGCACCTTCTATGC | 88300425 | 88300601 |
|  | CTTTGTTACAAGGGCTGAGTAGCT | CAAGTCAACATCTCTCCCTCCTTT | 88300856 | 88301209 |
|  | CACAGAACTGCAGCAGAGCTAT | TGACCCTAAATGAGAGATATGTCCCA | 88238544 | 88238915 |
|  | AAGAGACAGAGTTAGCATCATTCCAAAAT | CAAGTACAATACGTTTCACTCTAGACCA | 88239127 | 88239484 |
|  | AGTCAAGATAGGAATCCTCAGGTGAA | CTGCTTGAGTTTTCTTTTCTGCTATTTGT | 88239571 | 88239911 |
|  | AAAATATACTTCTGCCTCACAGCACAT | GTGTGCTTTGATAGTCTCAAATAGGTAGAA | 88240079 | 88240309 |
|  | AGCCAAGAATAGTTAAAATCACTGCTCA | GGGTGCATTGTAGCTGGAAATTC | 88240545 | 88240919 |
|  | AGTGTGTTATGCATGGGACACAT | GCTGATATCTTTGAGGGCTGACAA | 88241166 | 88241519 |
|  | GAGTTGAGCTCGCTGATGTTG | CCCAAGGCGCTGTATGATGT | 88242169 | 88242410 |
|  | CCACACTATGAGCTACTCAGCAA | CGCAAAGAACTCTGTTCTCTATAAATACAAACT | 88796559 | 88796933 |
|  | CACTCTCTGAATGCCATACTGTTCA | GGTCCTTTAGAAAATACATCTGAATTGCTG | 88780834 | 88781183 |
|  | GAATGAGTGAGAATTTCTGACATTAGAAGC | ATTGCCTTTTCAAAGCTGAGTATTTACA | 88330213 | 88330585 |
|  | CCTGGGTTGGACACACTGTATG | GTTGGTCATTGCATTGAGAAGCA | 88258354 | 88258713 |
|  | CCCATGACATGGTTTACTTAAATTGGAA | GCAAATGGACTCATTTGCTTAGGA | 88386265 | 88386636 |
|  | CGACGAGGAGCTCTGAGTGTAA | CATGACGACCTTTGCCGAAATC | 88241766 | 88242075 |
|  | ATAGTTTACTCTTCCCAAACCTGAAATTGT | AGAAGGCTTGGTACGCTGTG | 88780299 | 88780672 |
|  | TTTAATAGTACAAGCTGCCACAACAGA | AGACAAGTCAGAATTCACTTTGAAGAGG | 88237966 | 88238320 |
|  | CTCGTTGAAGTTAGCTGGAACATTTC | CTTCAGAGAATTGGCATTGGTCTCT | 88300541 | 88300915 |
|  | CAGTATAACTTGGTTAAACCACGTCTTCT | ACTCCATTGAATACATTGTATGTTGGACAT | 88238850 | 88239218 |
|  | TATCATGGCTCTTTTCATCTCAGAAAACA | TGATTTGGTTTTAAAATTGTGTGCGTGTA | 88237670 | 88238032 |
|  | CCTCATTCTTCAATAGACAGTAGAGACTCT | GGAAGACTAGGAGCTATTTCTGACTGTAAA | 88238249 | 88238606 |
|  | TTCTTGGAGCGGAAGGAAGAGGA | GGACAAAGTTCATGAGAGGAAGTGT | 88780619 | 88780894 |
|  | GCATGTGGAAGTATCAGATGGCA | GTGTTAATACCCTTTGGATCCTCATCA | 88239307 | 88239634 |
|  | GTTTTGTATTGGGAGGTTTGACTTAAAGTT | GACCCAGCCTTATACTTAAGATAGCC | 88239843 | 88240142 |
|  | AGAGATGACAGTGGATTCAGGTCT | GTGTCAAGTCAGCTTAGTGGAGATTT | 88240245 | 88240609 |
|  | CAGGCTGGAGCTCAAGAATGAT | CAGTAACTTGGGTCCAGTATTTACGG | 88240864 | 88241225 |
|  | CAAAGCACTTGGTATGGAACTGTTT | GTCGTCTCCCAAATATGACACTCTT | 88241460 | 88241823 |
|  | TGTCCTACGTTGAGTCGCAAATC | TTAGTTTTGTGTATTGCAGGTGAACAAAAA | 88781120 | 88781490 |
|  | GCCTCACATGGTTTCATTTACCC | CAATCTACTTTGGCAGCAACTACAA | 88300109 | 88300482 |
|  | CGAGTCCACCGAGTCTCTGA | CATGGAGCAGATCAGCAGTGT | 88241874 | 88242228 |
|  | AGTGCAGCGACGGCACATC | CATCCACATCAACAAGAAAGAAAACCC | 88242269 | 88242597 |
|  | CTTCAGTGCTGCCAAATTCCTG | GATTATACCCAATTAATGCAACACTCTCATTT | 88583015 | 88583389 |
|  | TTTCAGGATCAAGAGTTGGCTGTT | CAGGAATGTCGCGAGAGTAGAT | 88337821 | 88338192 |
|  | GCTTGTGTTCCCAAGGAAATTATTTGT | TAATGTATGTGAAAGGCCTTTCTGCT | 88323631 | 88324004 |
| HCN2 | CCTGCTTTCTGTATGCAGGTG | CACCATCTCGCGGTCGTA | 615536 | 615910 |
|  | GCCGTGATGAATGTACTGACGA | CGTGAGCCTTGTCAGTGTGTAA | 617047 | 617268 |
|  | CCCAGGTTCTACTGGGACTTC | GAGGCTGAGGATCTTGGTGAAG | 603539 | 603913 |
|  | GTCCTGCAGTGAAGGCTGTA | GCAGCATCATGCTGATGAGATTG | 604831 | 605130 |
|  | CCCACAGTACAAGCAGGTGTG | CGTCAAACATCTTGCCCTGGT | 610056 | 610364 |
|  | GTAGCCGTAGTTGGACGGA | GTTATTGCGTGAGCGCGAT | 616595 | 616919 |
|  | AGCCCGTCTCTCAGACGAGG | CCGAAGTAGGAGCCATCGG | 613131 | 613486 |
|  | GCCCAAGGTGTCGTTCTCG | GTCGCTGTACGGGTGGAT | 590305 | 590572 |
|  | TTTCTCTTGGTTACCTCTGAACATGG | CCCTCAGACCTTCTCCTGGTA | 607826 | 608191 |
|  | CGCCACGTGCAGAGATCT | CCATGGCCACACTCCTGATG | 613840 | 614183 |
|  | GCGCCTTCCTGCAGATCTT | TCAACCCTCTGTAAGCGGGATA | 605047 | 605313 |
|  | AGCGACTTCAGGTACCGC | CCCTCTATTCGTCCTCTCTGC | 590567 | 590899 |
|  | CAGAACCACTCGTGGAGTGAA | CCTGGCCTGACTCTCTCTCA | 607961 | 608264 |
|  | GCTGACTTCCGCCAGAAGATC | CAGGTCTAGCTCGAGTCCCT | 610301 | 610538 |
|  | CGCCTCTCGTCCAACTTGTG | CGGCTACCTACGTCTCGTC | 616454 | 616601 |
|  | GTCCGAGGAGGATCGTTTTCTAAG | GCGCCAATGCTGTCGTTTATTG | 616842 | 617156 |
|  | CGTGGTTCGTGGTGGACTTC | CCCACCCATGCACCATTATAG | 603777 | 604072 |
| HLA-DQB1 | GTCATTTCCAGCATCACCAGGAT | TAGACGTGGTGTGTGTTCTCAC | 32629818 | 32630088 |
|  | CTACAGACAGGACCACTCTGAAC | ACTCTCTCACCTATTCACTGTATCCAAG | 32629050 | 32629422 |
|  | GGGATGAAAGGAGATGACCTGG | GCATCAAGCTGAAGTTCTGTGTC | 32627857 | 32628216 |
|  | AGAGAGGAAATGTTGATGAAAGATTGTGT | CTTTTCCGAGGGACCATCCAAT | 32634153 | 32634515 |
|  | CTGGGATATTCTAAGGCCAATGCA | ACTGCTCAAGAGAAGCACATGAAAA | 32627422 | 32627739 |
|  | GTGGAATGAACTGGGCTCAGA | GCCGAGTACTGGAACAGCC | 32632463 | 32632686 |
|  | TGACATCAGGGATAAGAGATGGGA | ATCCAGGACAGAGGCCCTCAAC | 32629653 | 32630000 |
|  | CACCAAAGTTAAGGCTTGGTTCTG | TGGGAAGAGAATGTAACTCTAAGTCATGT | 32628441 | 32628763 |
|  | AAAATGCTTTCAGTGTCTTTTCAAGGT | GTCCTACAGTGGCTGTCACG | 32627530 | 32627904 |
|  | TGCCCAGATAGAGACATAACAGAAAGA | CAATTGTCGCATCATTCATGGTTCTTTAAT | 32627114 | 32627487 |
|  | AAATAGGATGTGGGAGAGGAGGAA | GTGACTGTATATCCTTCAAAGACCCA | 32549238 | 32549604 |
|  | GCCTGGGCACAATGTTAACAAA | TGCTATTGAACTCAGATGCTGATTGG | 32557345 | 32557692 |
|  | TTGGAAAATAATCTTCCCTTTTTCTTTTCATT | CCTGCTTGGCAGTTATTCTTCCA | 32546481 | 32546805 |
|  | GCTTCACCTCTCACTAGGGAAAC | TGTTGGAGGACAGATTTGCTTCAAT | 32547965 | 32548108 |
|  | ACACACTCAGATTCCCAGCTC | CAGCCTAAGAGGGAGTGTCATTTC | 32551830 | 32552141 |
|  | CTGTCGAAGCGCACGGACT | GCCGGTTAAGGTTCCAGC | 32552044 | 32552268 |
|  | CGTTCAGGAACCACCTGACTTC | AGACTTACTCTGTCTTCCTGACTCAT | 32549496 | 32549666 |
|  | GCTTTCACATAGTAACCATGCACTGA | CCCATTTTCAAAGCTCTAAATCTTAGAGTC | 32548347 | 32548720 |
|  | TTTGGAGCCAAATGGACCAGA | GATGACCACATTCAAGGAAGAACTTTC | 32546520 | 32546861 |
| HRH3 | CACTTCTGCCCACAACTCAGAA | TGTATGTACCCTACGTGCTGACA | 60793441 | 60793695 |
|  | GGTCCTGGGTGAGCAAACAG | TGAACTTGATGTTCCTGGGATGTTTAA | 60790225 | 60790437 |
|  | GTGCGCCTCTGGATGTTC | GCCTTCCTGCTGTACGGAC | 60791714 | 60791913 |
|  | GCAAACTGCTAGGGCAGGAAG | GGCCCTCTCTTAATGCCACG | 60790686 | 60790945 |
|  | CAGGGTTGACAGCCGAGTT | CTGGAGAAGCGCATGAAGATG | 60791172 | 60791403 |
|  | GCCTGAGTGGCAAGGAACTT | ATGGCGCTGCTCATCGTGG | 60794592 | 60794906 |
|  | CCCTGAGCTCCTCAGCAATTTT | GTTGGTGTTCTTCCCAAAGCAAG | 60790378 | 60790748 |
|  | GCTGCCGTGGCATTAAGAGA | ACTACTGGTACGAAACCTCCTTCT | 60790921 | 60791228 |
|  | GTGAAGCTCTGGGACACCATC | CTGAACATCCAGAGGCGCACC | 60791366 | 60791733 |
|  | CGTAGCTGATGAGCACGATGTT | GGATGGCTAGTTCCAGAAAAGCAG | 60793573 | 60793835 |
|  | GGAGTCTCTCCTCAAAGCACG | TAAGGCTTCCGGCTGAGCT | 60789962 | 60790335 |
|  | AGGTACTCCCAGCTCAGGAT | TCTGAGGCTCTCACTGAGTGT | 60791870 | 60792199 |
| HTR1A | GTGACCGCCAAAGAGCCAATAA | GCGGCAACACTACTGGTATCTC | 63257305 | 63257482 |
|  | GCGGCAGAACTTACACTTAATGATC | GTGGGCAACTCCAAAGAGCACT | 63256284 | 63256655 |
|  | TTCTCCACCTTTTTGACCGTCTT | CATCGACTACGTGAACAAGAGGA | 63256843 | 63257124 |
|  | GCGGCTGTGTGTACAGTTTATT | GATCCGTCCCATTCACTATGCTT | 63255887 | 63256243 |
|  | CCAAGCAGGAAGTTCTTACTGC | ATAGGGAGAGGAGGGTCACAGAGT | 63257670 | 63257988 |
|  | CGCTCATTTTTCCTCTCGAAAGAG | TGCTCATGCTGGTTCTCTATGG | 63256567 | 63256921 |
|  | AATAAGCCAAGTGAGCGAGATGA | CTGCAGAACGTGGCCAATTATC | 63257058 | 63257348 |
|  | GTGCACTGCACGAGAGAGTTAA | GATGTTTAATGCTTAGTTCAAGGCAGAAAA | 63255599 | 63255949 |
|  | GGCGAATTATCTTAAGTGTTGATTCCCT | CGCATACTTCAACAAGGACTTTCAAAAC | 63256182 | 63256347 |
|  | CAATCCCAGAAATATCTAGAACCGAGAA | AATGCAAAGACGCTGAGCTAGA | 63257919 | 63258253 |
|  | AGAGGTGATCACTTGGTAGCTGA | GAAAGCTGCTCCTCGGAGATAC | 63257427 | 63257724 |
| HTR2A | TGGAAGAGCTTTTCTGAAGACAAAGAA | ATGCTCTTGCCTTCATATACTTAATTCCTT | 47409501 | 47409837 |
|  | GGCTTGAGATTGAGGTGCGTAT | CAAGTTTGTTTCAACTGTGAGAGTAGTTTT | 47407248 | 47407395 |
|  | GTGGCATGCACATGCTCTTTAT | TCTCTCCTTACTTCATCTCCAGGAAAAA | 47469569 | 47469847 |
|  | GGTGTAGAAGGACTAACAGGTTATAGTTTC | TCGGCATAACCAACAAAATGAGATATAGTT | 47470069 | 47470443 |
|  | TCGAATATCAGGATGATACCTGGAGT | ACAGCGCCATCTCATGGTAATT | 47405581 | 47405933 |
|  | GGCCCAAAGGTACCATTTTATTTAGG | TACAGACATGTTCTCATGCACTTTATTCA | 47406183 | 47406502 |
|  | TGCAGAGTTACAAGTGAAGGCAA | AACACTCAAGGTTAAATTGCATGTTGTTAT | 47406696 | 47407066 |
|  | CATCCTCAATCCTGACACAGTTACA | TGACAACATTTTTCATTCCTGCTTTTTCAT | 47408051 | 47408424 |
|  | GCAATAGGTAACCAACTCAATCCCA | GCTACAAAATGTGTGCTTGGAAAATGT | 47408590 | 47408809 |
|  | TCAGCAGGCACACATTTAGAAATCA | GGAGAAGAAAAAGCCTGTTTGGT | 47471061 | 47471289 |
|  | CGGCAACTAGCCTATCACACAC | ACTAGTCTACACACTGTTCAACAAGAC | 47408958 | 47409257 |
|  | GGAAATGGCAGTGGTGTGAACT | GGTTCCACAATCATCTATTGAGTGTACATT | 47407542 | 47407909 |
|  | AGGTTTCAGTACAACAAAATGGAACAAG | CAGAGCTGGAAATTGTCATAGGATAGG | 47466437 | 47466811 |
|  | ACTGACACTGAATATACCGTGAAAAGG | GGCCAAATTAGCTTCTTTCAGCTTC | 47409193 | 47409563 |
|  | GTTAGAATAATCACTACGGCTGTCAGTAAA | CAGAGTGTGGGTACATCAAGGTG | 47469779 | 47470132 |
|  | GTGACTTAGTTCAATTTGGCTACGGTA | TGGCGAAAACAATGGTCCACATATAT | 47405874 | 47406245 |
|  | TTTCATTGACTTGCTTTTCGTCCATATAAA | GCTGCATTTGAACTGAACTACTCCTT | 47406433 | 47406805 |
|  | CTCATTCAATAGAAATGCTTCAGCATTGT | GAAAATATCACTTGAGGCTTAACATTGCAA | 47406983 | 47407310 |
|  | GCCAGCAACCTTGTGAATATTTCA | GAAACCTTGCTGCTATGCTGTT | 47408360 | 47408647 |
|  | AGCATGATTTCAAACCGGAAAGAAAATT | GCAGTTCTTTTGTGCGACTTTGAG | 47470746 | 47471120 |
|  | AAATAAGTATCAGAAAGCTCACAGCTGAA | GACAATAGCGACGGAGTGAATGA | 47408743 | 47409013 |
|  | TGGTGTTTATTGGTAACCAATCAGGAA | ATTATCTGATCCTTGTGGCACACAA | 47407328 | 47407663 |
|  | TCGTTTGAAGGCAAGGATTTTGTTTTAATT | GCATGCACTCTTTTGAGCAGTAA | 47407809 | 47408109 |
| IL10 | GTCTTTCCTCATTTACAGCTAGCTCT | ACCAAGCTGTCCTCTTAAGCTAGT | 206944119 | 206944493 |
|  | CCCAAGCCCAGAGACAAGATAAATTAG | TGTGAACGATTTAGAAAGAAGCCCAATATT | 206941544 | 206941802 |
|  | GCGCAGGAGGAGGGTTCTTATA | AGGCCTCCCTGAGCTTACAATA | 206945526 | 206945889 |
|  | CGTTAGCCTTGAAAATCAGCATGA | TTTAACTAGAATTTATTCAATTCCTCTGGGAA | 206940674 | 206941048 |
|  | CCAGATCCGATTTTGGAGACCTCTA | AACCTCTGCACTCAAGGTCATG | 206941916 | 206942177 |
|  | GCTGCTGGATGTGCTGAGTTAA | CACTGTTGAATCCTCTGTTTTTAAAACTCC | 206944556 | 206944922 |
|  | AGGTTCCCACACTCTCTCCAAT | GTCCTGGAGAAATACATTTAACTCCCA | 206943048 | 206943422 |
|  | CTATGGAAACAGCTTAAAAACAGGTGAAAA | GGGTGGCGACTCTATAGACTCT | 206941720 | 206941973 |
|  | CGGCCTAGAACCAAATTTAGGTT | CTTTCTAGCTGTTGAGCTGTTTTCC | 206941370 | 206941606 |
| IL1B | GGCTTGATGACTTCCAAGAGGA | CTCGGGATTCTTTCAAGCCCTT | 113590891 | 113591265 |
|  | AGAACTCTCACAGCCCTGTTTG | TTCGCTCCCACATTCTGATGAG | 113587254 | 113587627 |
|  | GGAGCGAATGACAGAGGGTTTC | AGAGAGTCCTGTGCTGAATGTG | 113587620 | 113587923 |
|  | TAGACCTGTTCCCAGCTTTTCCTA | TCTTAGGCCAAGGAACCTCACT | 113588759 | 113589133 |
|  | GGGACTTGTAATGGGAAAGTAGTCTTTAAA | CTAGACCCAAAGGACTTCTCTTTCAC | 113593595 | 113593969 |
|  | ACCTGGCATTTTTGCAAAAAGCT | CCCTAAGAAGCTTCCACCAATACT | 113594121 | 113594495 |
|  | TTTTCACTTGTGTTGATCATTTGCCTTAAA | GCTCAGGTGTCCTCCAAGAAAT | 113590126 | 113590500 |
|  | TAGTGAGTGACTGTGGTGATATGATGA | GGTGTTTCAGGCAGCTTTGAGA | 113592950 | 113593324 |
|  | CCAGTCCAAATTGAATTGATTCCATAGCTA | GGACTTTCCTGTTGTCTACACCA | 113587466 | 113587840 |
|  | CTCAAAAACCTTTCTGTTCCCTTTCTG | TGGCCAACCCAAAGAACATCTT | 113587855 | 113588229 |
| IL1R2 | GTAAACAGTAAAGCTCTGTGTGAACAG | CTGTCTCCAAAAGGAAGAGCGAA | 102640818 | 102641192 |
|  | GGATTGCAAATCCCACATTGGTTG | TTCCAAAGACCTGCAAGGAATTCT | 102636000 | 102636374 |
|  | AAAACAATAAAAGCGCAATGGGTACT | AGACAATGCAAGGGAATTTCTATGGA | 102642458 | 102642832 |
|  | CCGTGAGGAGGAAAAGGTGTG | CATCTCTCTCTCTTCTCTCTGCCTT | 102608307 | 102608649 |
|  | AGTTTTGTTCTTAAACAGAGTAGGCCAAA | CCCACACTTTGCTTTCTGACAA | 102624839 | 102625204 |
|  | ATGGCATAAAAATGACTCTGCTAGGA | GCGGTGTCAGCTAAAATGGCTA | 102626160 | 102626534 |
|  | CCAGGTGCAGGGAGGTTTTATG | CGCACAGGGAGACTTACCTAGT | 102625950 | 102626305 |
|  | AAAGCCTGACTCTCATAATGACTGG | GGAAATATTTCCTCACACTTGTTATTCCAG | 102638518 | 102638892 |
|  | CATTGCCCTGTCCTCTTGTACA | TCCTTTACTACAAAGCCACTATAAATGTGG | 102644621 | 102644995 |
|  | GGCAAAACGCCCATCACTTTAAAA | CTCTCTCTCTCTCCAAAGCCTCTTA | 102608148 | 102608521 |
|  | GTGGAGGCTGCATGGATTTTTC | AGAAGAAACAAGGACAGGAAGATCTG | 102615290 | 102615664 |
|  | TTTAGCTTTACTTTTATTTTGCCTTGCCAT | GAAAGGCAAACCATGTGTGTGTT | 102632276 | 102632650 |
|  | CCGTGTAAGGTGTTTCTGGGAA | TGTTTAATGAAACAGCTACAAAATGCAACA | 102641012 | 102641386 |
| IL4 | CTGGTGTAACGAAAATTTCCAATGTAAACT | CTCTAGCAGCTGTTAACGTTTTCATG | 132009590 | 132009964 |
|  | GCAAACTCACCTCCATTTGTCCT | TGCAGCTCCTAAGGAGTGTTTAGA | 132015294 | 132015668 |
|  | CTAGAGAAGTTGGAACTGGTGGTT | TCTCGTTCTTTGAAAACATCTAACACCT | 132009959 | 132010333 |
|  | CAAGTGCCACAGTAGGCTTGAT | AAATTGATTTCCCTAAAAGTTGCAAACCA | 132018092 | 132018449 |
| IL6 | CCCACATTTCACATTTGAACATCATCC | AGTTCTCCATAGAGAACAACATAAGTTCTG | 22770920 | 22771291 |
|  | GAAAGTAAAGGAAGAGTGGTTCTGCT | GATTTCCTGCACTTACTTGTGGAGA | 22766560 | 22766916 |
|  | AGGAAATCCTTAGCCCTGGAACT | CCGTCGAGGATGTACCGAATTT | 22766909 | 22767231 |
|  | GAGAACTAAAAGTATGAGCGTTAGGACA | ATTAAGCTTCACGTGACACACTCA | 22771285 | 22771648 |
|  | GCTTGGAACTGAACCCAAGTGT | TATGTATACAGGCACTGCATGCAA | 22768131 | 22768503 |
|  | CTAAGAGGTACTTGAAGTTCTCTAGAGGA | GCCTTCTCTTTTGGAAGTGGCAT | 22769008 | 22769382 |
|  | ACTGGAGATGTCTGAGGCTCATT | GCGGCTACATCTTTGGAATCTTC | 22766799 | 22767165 |
|  | CAGCCACTCACCTCTTCAGAAC | AAAAGGAAGCCCTGAGAAGCAATA | 22767176 | 22767527 |
|  | CTGCAGGACATGACAACTCATCT | CTGGCTCTGAAACAAAGGATATTCAAAC | 22771109 | 22771483 |
|  | AGAGCCAGATCATTTCTTGGAAAGTG | TGGTGGCAGTGACAAGAAACTT | 22771476 | 22771676 |
| KCNS1 | GTGCAGCAGGCTCAGGAAGAAG | GGATTTTAACCTGGTCTTCCAGGAA | 43727065 | 43727405 |
|  | CCTGCTTCATGAATGGCTTGGA | GGAGACATCCCGAGAAACCTCT | 43723273 | 43723601 |
|  | CCGGTAGAAGTGGGAGAACTTG | ACTGTAAGCCCATCCACTCTGA | 43723697 | 43724028 |
|  | TCGCGTGTCTGTCCCAAAG | GCCGAGTCAGGAATAGAAACCG | 43729586 | 43729811 |
|  | CAGCCAGCAGCGTGAGATA | CCTCTGGCTGACCATGGAGA | 43726446 | 43726798 |
|  | GACCACAAACAGCCTCATGGTA | GTCATACACAGGGCCTAGTACATAGT | 43721027 | 43721374 |
|  | TCAAGCAGACAATGTCTTCCATCTT | GGCTGACATATATGAATACTGGCTTCTAA | 43722136 | 43722506 |
|  | CCCTACTAGTCGGTAAGCTTCATG | CCTGCTTGGTTCTTGAAGTTTTTACAATAT | 43721592 | 43721957 |
|  | ATATGTCAAAGCCCTGGGAAAACA | TGGATGAAGGGATTTGCCCAAT | 43728712 | 43729086 |
|  | AAGATTATTGCTTAGCAAATCTTGCATACC | AGGTTTGGAATCTGGGCTTGTT | 43722746 | 43723088 |
|  | CCTGGCTCTCTAGATCTGCAGA | CCTACACAGCTGAAAAGGAGGA | 43723546 | 43723911 |
|  | CTTGCTCGGCAGCGAGTA | GCGCGAATTCTACTTCGACC | 43726753 | 43727116 |
|  | CCCTTTTTGACCAAGTTTGTTCCA | GGAGAAAGCTCAAGAGGGAATCATC | 43720751 | 43721084 |
|  | GTATTTGTTGAGTGAGTGAGTGAAGAAC | TGTGGCTATCTCTGTCCCATCA | 43721889 | 43722193 |
|  | GAACTGCCAAGGTCAGACTGTT | GGAGGAAGTACCATAGCATTGTACTTG | 43722445 | 43722813 |
|  | GGGATAGTCACACTGCATGTGA | TCAACCTCATCGACATTGTGTCTG | 43726125 | 43726499 |
|  | GAGACCCAGAATCCATGCGTAT | GTCTCAAGAGATACAGTGGTGGAAAAATAT | 43721316 | 43721656 |
|  | CACCTAGTGGGTGCTCACATAG | TCTAAAGCAGGAGCTCTCAAACTTG | 43727661 | 43728027 |
|  | GCACCTCCTATCTGCTAGTCACT | GAGGCGAGACCGAGTATAGC | 43729273 | 43729646 |
|  | ATGGAGTTGATGGAGCATTGCT | CTGCAAGCAACTCAGAAAGCTTT | 43723034 | 43723328 |
| KIT | GTCAGTCCATATGTCCAGTTGCA | CCAATTTGCAACCTAAGATTAGGAGAGAAG | 55603221 | 55603573 |
|  | CCACTGAAGCTGAATATTAATGGCCA | TCATACCTCAGAGTACCTCAGTTCATTTT | 55595391 | 55595764 |
|  | AGGAAAATGTCTCTGGACAACATTGT | CTCAGGACTTTGAGTTCAGACATGAG | 55593867 | 55594231 |
|  | CCGGCATTAACACGTCGAA | CAGGCTTCGCCGAGTAGT | 55523944 | 55524299 |
|  | GCTCTGAGACTCACATAGCTTTGC | TATCTCCTCAACAACCTTCCACTGTA | 55593249 | 55593623 |
|  | GCCTCAGGAAGGTTGTAGGGAT | CTTCTAAAAAGCCACATGGCTAGAAAAA | 55589654 | 55590008 |
|  | GGAAATCAACCAATTGTTTTTGTAATTCCA | GAAATAAGATCTGATAAGCCCACTGGAA | 55573162 | 55573525 |
|  | GCTTCTATAGATCCTGCCAAGCTTTTC | GATCAACGAGAAGAGAAGTCTTCTTTAGAT | 55564440 | 55564806 |
|  | CATCCATCCAGGAAAATCAGACTTAATAGT | CCATATGTCATCCAAAATTAAGAGCAGGAT | 55561724 | 55562022 |
|  | GGAAAACACCATAAGGTTTCGTTTCT | GGCTAAACCTATACTTTATTTCCTGCTACA | 55605434 | 55605783 |
|  | GTATTTGCAGTTCACCTGCACTTAAG | AAAGGTGCGAGAGCATAGAACTC | 55606029 | 55606402 |
|  | TGGCTTAATGTTTGAAATTATTTTGTGGCT | CAGGATGATTGCACTTTAATGCTTTAAAGT | 55606634 | 55606948 |
|  | TTCCCAATCGTTAATAATGACTGTCTTTCA | ACCGTGATGCCAGCTATTATATTTCTC | 55569667 | 55569928 |
|  | TAGGTAAAAGGTTTTTGTGAGATGGTACTC | GCTGCTTCCTGAGACACAGTTT | 55602524 | 55602894 |
|  | GCCTGCAAGTTCACATTAGTTCATT | CCACACTTCAAAATGACATTCTGCATTTTA | 55597919 | 55598293 |
|  | GCAATCCTGTCTTTCTGAGCACA | GTGTTCATACTCTGTCCTTTGTTCTAATGA | 55604839 | 55605199 |
|  | TCTGACCTACAAATATTTACAGGTAACCAT | AAAACTCATTGTTTCAGGTGGAACAAAA | 55593469 | 55593819 |
|  | TGATTATGTGAACATCATTCAAGGCGTA | ATCGAAAGTTGAAACTAAAAATCCTTTGCA | 55599053 | 55599417 |
|  | TTTCCTCAAACAGGCATAGATTTCCA | TGTGAAGGTCAAGATTATCATGAAACGTTA | 55575489 | 55575833 |
|  | CAAACTTTACATGACTTTCCTCAAATTGGT | GGCACTGCTACCATAAAGCAGAA | 55597359 | 55597713 |
|  | GTTTTAAAAGTATGCCACATCCCAAGT | GACATGGTCAATGTTGGAATGAACTTAAAA | 55591936 | 55592309 |
|  | AGATAGGTTAGCACCATGCTTTGT | GCACTAGTCGAGGCAGTTTCAG | 55565659 | 55566031 |
|  | GACCAAATGTGACCCTCAGGAT | CAGTGCATAACAGCCTAATCTCGT | 55561454 | 55561788 |
|  | AGCTGAAAACCTAAGTCCTTTATGTGG | CTTCTATACACAGTGGACATAATGCCA | 55605132 | 55605497 |
|  | TGGGAAAACACTGCCATCTTAGT | AGGAACAGGTACAGTAAGATGAGTCTAAA | 55605720 | 55606094 |
|  | TGTGGCCGTTATCTGGAAGTAAC | GCTTGGGAATATTCAAAAGACATTATTGCT | 55606346 | 55606715 |
|  | GCCATGACTGTCGCTGTAAAGA | AAGAGAGAACAACAGTCTGGGTAAAAA | 55594063 | 55594417 |
|  | AAGTATGCCTTTTGTTGCTATGTTCG | GATGGTGGCTGATGACAAAAATCATC | 55604527 | 55604898 |
|  | TTAGGAGGGTTGCTTTTATGACACC | CGTTGTCTTCTTTCCCATACAAGGAG | 55564131 | 55564503 |
|  | TTCTCTTTAGGTAAAATGATCCTTGCCAA | GTGTCATAAAGAATCCAAGTGACATTTCAA | 55602766 | 55603113 |
|  | TTTCTCCTTTTCTGAAACCAGCAGA | TGGTTTTGTGCTTTCATTGCAAGA | 55569866 | 55570227 |
| LTB4R | TGTCCTCTGCTCTGTGGTACTT | CTTTGCACCTTCTTTCATGGCT | 24784062 | 24784298 |
|  | CCCACAGGCAGCTTTAACCATT | ACAGCAGTGCTGTTTTCTCAGAA | 24786105 | 24786452 |
|  | CAAAGATTCCCAAAAGTGAGAGGGAT | ACAGATGCTGGTATATGGGTCTTGA | 24786695 | 24787002 |
|  | GCATTCTGTGTGATACCAAGGAGA | AGGAGGTATGGTAGCCAGAGAAG | 24783432 | 24783806 |
|  | GCCTTGGCCTTCTTCAGTTCTAG | AGCCAGATCCAAACTCTAGTCAAAG |  |  |
|  | ACTGCTCCCTTTTTCCTTCACTTC | GCACAGGCTCATGTTCGTTTTC | 24785065 | 24785361 |
|  | CGTGGTGAACCTGGCTGAG | AGGAAAGTGCGCCTCCTTC | 24785571 | 24785943 |
|  | ACCACAACCTGCCAGGAATTTTTA | TCTTTCCTATCTTCTCACAGCCTACTATT | 24783079 | 24783331 |
|  | GACCTCAGTGGCCACCATTAT | GGAGCAGATCACTTGATGTTAGCAG | 24782683 | 24783039 |
|  | CTCTACTTTAGCGACTGCTCACA | GTGCAACAAAGGAAGTTAAGGAACCT | 24782161 | 24782513 |
|  | CCCTCTCAAGTTAAACGAACTGAAC | CACTCACAAGGTTGACCAAATTTCAG | 24785889 | 24786163 |
|  | GGCTTATCACTCCAGGTTCTGT | GTTCTCTAAAACAACAGTCATCTCTAGGA | 24786397 | 24786760 |
|  | CCAAAGTATGAGCCAGTGAGAATGAG | ACTCAAAACAAAAATTTGCCCTTTCCT | 24786941 | 24787298 |
|  | CACGGGATTCTTTCTGTCCTCAT | CCTTTCAACTCTCACTTCTCAGGAA | 24782454 | 24782739 |
|  | CGGCCTCAACTTTGTGTGTCTA | CTCCATTCTCTTTGGTAGAAGCCAA | 24783270 | 24783491 |
|  | ATATCAGCATTGTAGCCTCCAATCTG | AGGTCTAGGTGCTGTTTGCTAAATC | 24783747 | 24784119 |
|  | CCTGAGCCTGGAGACTCTGA | GCGGTGAAGACGTAGAGCA |  |  |
|  | GAGGTCAGTGTTCTGGGACATT | CTAGGTGGCTTCAGTACAACTCAG |  |  |
|  | TCAGGAAACCCTTGGTCCTCTA | CTCCGCAGACATAGTGACACAG | 24784789 | 24785152 |
|  | CTCGCGTACCGCACAGTA | CGAGTGCGATGAGCACGTT | 24785314 | 24785677 |
|  | GGTCAGATTGAAGGAAGGACTTTTTAGT | CCTTGAAGTCTCCTGCAAAAGC | 24782757 | 24783131 |
| LTB4R2 | GCTCTTTGTGGCCTTCCTGAC | TCCACAGGTGGCGGTAGAC | 24780098 | 24780360 |
|  | CCAGACTAGAGGAGTGGTGGTA | CAGCTCAGCAGTGTCTCGTT | 24779549 | 24779917 |
|  | GAAGGATGTCGGTCTGCTACC | CGTAGTACACCGCCTTGCA | 24779866 | 24780168 |
|  | CCACTTCAGCTTGTGCTGTTTC | TCACTATCTCTACCACCACTCCTCTA | 24779208 | 24779580 |
| NF1 | GGGAAGTGAAAGAACTTGAAAGATTCA | CCTGTCATGGGTATTATTTCTTTGCTTTTT | 29559024 | 29559297 |
|  | TGGACAGTCTACGAAAAGCTCTTG | ACGAAGAGTCAGAACTTTAATGTTAGCAAT | 29527450 | 29527723 |
|  | GGGTTCTATGATTTCAGTGATGTCTTCAG | AGGCTTATTTCAAACAAGTCACTCTATTCA | 29556255 | 29556546 |
|  | CGGCCTTCACTATGTAAAGGTCA | TCTTGTCTGGAGATCCTTGTGGTAA | 29559678 | 29560051 |
|  | CATTTTTGCTACTCTTTAGCTTCCTACCT | ACGAATAGGAGTTTATTATACCTGCTCCAT | 29562549 | 29562923 |
|  | CGCTTCGACGAGCAGGTA | CGGAAGGGCTGGGATAAAGG | 29422373 | 29422675 |
|  | TCAATTTGGAAGCCTCTTGTTACATATGT | GCTATATGTTTACTTTACTGAGCGACTCTT | 29554353 | 29554727 |
|  | CCAGTCTACTTTTAGGAGGCCCTTA | CCTACTGTCTCAAAGCTAAAATCACTATCA | 29684154 | 29684521 |
|  | TACTCTCTCAACTGTATGTCCAATGTAACT | TTGTGAATTATTTTTCTGGTTTTCAGCACA | 29685773 | 29686145 |
|  | CTGTTTATGGAATTTTTCTTTCCTAGCAGA | TCCTGAAAATGAATGTGAAACTATCACTCA | 29508249 | 29508623 |
|  | AATGTACATTAAGCTAGCTACCAAGATCAC | AAGAAAGTAACATTCAACACTGATACCCA | 29665563 | 29665886 |
|  | CAGAATGCATTTGTGTAGTTGCTTAAATG | TACACACCTTGAGTAAAATAAACTGCTTCA | 29509376 | 29509750 |
|  | AGAGTTTTTATGCAAAGTTTGACCTTTGAA | GCACATAACTGAAAACCATAGGGTATTTCA | 29585266 | 29585640 |
|  | CCAGAAGTTGTGTACGTTCTTTTCTAAA | CATGTCCTCCTTTCTACCAATAACCG | 29557129 | 29557503 |
|  | GGGTTTCTAGTGAATCTCCTTCAAGT | CCAAATTTCATTCAGAAAACAAACAGAGCA | 29551961 | 29552335 |
|  | GAGCTTTCTTTGAGTCCTCAGTGA | GAGAAATGGCAAACGTTTCTATATTTCACT | 29683354 | 29683725 |
|  | CTGGGAGGTAAAATGGAAGACTATTGTT | TCTCAGACAAAAGTCCATAGGACTGT | 29485911 | 29486226 |
|  | CACCACTTTCCAGGTTGGTTCT | GCCTAAATTAAGTAATGCGATATTGAGCAG | 29654504 | 29654837 |
|  | CCAAAATATGTGCACATTTAACAGGTACT | GCCATCTCTAGATTTCTCCCATGTT | 29677080 | 29677453 |
|  | CTAGCAATCGCTTTAAAACAGACTTTCT | TCTAGTTAGTCAAGAAAAGCAATGAATCGT | 29652951 | 29653325 |
|  | TATGCATACTTGTCATGTAGAGTTATCCCA | ATGAGAACTCTGTCAGACTGAGTCT | 29664128 | 29664430 |
|  | AATGCTTAAATAAAAACACTTGCATGGACT | CAAATCTGCAATTAAAAGATCCACAGAACT | 29664703 | 29665047 |
|  | TCCTTCTTCAACTAGATTACAGATCTGCT | AGTTGGAGATAAGCATGTCTTGTCAATTAT | 29663336 | 29663703 |
|  | CTATTGATCCACATTAGGACCATAAGTCC | GGTACAAGTTAAGGCACACAGAAGATT | 29656981 | 29657353 |
|  | GTGTTTGCATGGTCTTAGAAAGTTCC | AGGAAATGAAGGACCCATTCAATTCT | 29533134 | 29533507 |
|  | TGAACAAGCCCTCCATATTTGTAATCT | AGTCCAAGAAGATGCAAAGTAAAAAGC | 29587264 | 29587638 |
|  | ATTAAGTGAGCCTTTAAAGAAAGCTACTGT | AGTAAGACATAAGGGCTAACTTACTTCAAT | 29669959 | 29670266 |
|  | TCCAATGAAGTCTACACGTTGCA | GACTCAGTATTATAGTTAACCACACACCAT | 29575882 | 29576250 |
|  | CTGCCTTGTTTCTTGCTTTCGTA | AGCCAGGCAGATCTATAGAAAAAGAAAAA | 29528126 | 29528440 |
|  | TTTTCCATTACAGCAAACACAAATTCCA | CTAATGTTGGTGTCTTATATTGTTGCTCAA | 29687492 | 29687778 |
|  | CCCAGTATGTTTAATCTTTTAGCTGTGGA | GACTGAGTTTGGATAAGGACTTAGAAGC | 29701641 | 29701914 |
|  | ATCCGCAAGCCAAGTGCAGAAG | CAGATACACGCCAAAAGTAGAAGAAAAA | 29701101 | 29701398 |
|  | TAGATGTTTGCATTAGCTCCATAGCAA | TACCACAAAGAAATTGGATCTGCATTACTA | 29702140 | 29702500 |
|  | GGGAGTTTCCTGTAGTGCTGTT | TCTTCTAATGCAGCCTCTACAATAGTTTAA | 29702674 | 29703036 |
|  | GGTTTGGTGCATATGATAGTGGGT | GTGGTGAGAGGTTTATCTCATGTACATTTT | 29703269 | 29703603 |
|  | TACCTGTTATGCCAGTACTCCCAT | GCTGACACTAAGAATGAATTTTTGCCTAAA | 29703818 | 29704187 |
|  | ACTTACACTTCCAAAGGTTTTATGGTTTTG | AAAAATACAGTAGTAACCACATTGAAATCTGAA | 29549043 | 29549246 |
|  | ACTGCGTTACATCATTTAAAGAAAATGCTG | GGGAAGAAAACAGAGCTCGATACATC | 29704390 | 29704746 |
|  | ATTTCTATTTTAGCAACCAAAGGACACAAT | GGGCAAAAGAACTTGTCTCAAAAAC | 29490191 | 29490523 |
|  | ATTTGCCGACAAGCCCAGA | GTGCTTTGAGGCAGACTGAGT | 29553486 | 29553748 |
|  | GCTCTCAATCTCTAGCTCGCT | AGGAGAGGGTCTGTGGAGTG | 29421939 | 29422229 |
|  | GGAGGAAAATGTAAATGTGTAAACCTCAAG | TATTGAGCACTTTAAGTGTGGCTTGTT | 29549475 | 29549845 |
|  | AAATTTTTGGTGCATGTTGCCAAATTAC | TCAATTCCTGTTAAGTCAACTGGGAAA | 29592088 | 29592462 |
|  | GCCTGATTCTAGGTAATAGTCTTTACCTTT | TGGCACCAGATAAATATGTGCACA | 29661737 | 29662111 |
|  | CCAGCCTAGTTCTAGAACATTGTTATCA | CATACCATTTCCAGAAATGACATCTACCT | 29545884 | 29546257 |
|  | CTTTTTAGGTTACAGGAATTAACTGTTTGTTCA | TCATGATACTAGTTTTTGACCCAGTGATTTTT | 29496901 | 29497075 |
|  | GCTCAGAATTCACCTTCTACATTTCACTAT | CCTTTTTGAAAACCAAGAGTGCATTTCTTA | 29528438 | 29528602 |
|  | TGTCATGGAAGAAATGTTGGATAAAGCA | CGATCCATAAATTTGCTGACAGGTGTAT | 29555951 | 29556323 |
|  | TCTGGCTTTTATGTCTGTGATAGCAG | TCAAACACAAAAGTTTGACATCTCAAAAGT | 29557695 | 29558069 |
|  | GTTAGTAAATTTGCATCTGTTTGTCCACA | ACTAAACATCTTTCTTCTGGCTCTGAAAT | 29559987 | 29560359 |
|  | ATGGAAAAGTGAAGAGCTTACTCATATCT | AGAATAGCAACAAGAAAAGATGGAAGAGT | 29667364 | 29667737 |
|  | CTGACCTTATGCTTACTATTGAGTGTTTCT | AAGGTCTTGGCGTTTCAGCTAA | 29541306 | 29541678 |
|  | GTCTCGGACTGTGATGGCT | CGGTTACCTGCTCGTCGA | 29422020 | 29422394 |
|  | GTAGACATGGTCCTGAGGTCTTTTT | GGTGGCAAACTCTCCTTCTCAA | 29588586 | 29588938 |
|  | CATTTGAAGCATTTGCTCTGCTCTT | CTACAGCCAAGTTTCAAACTTGATGTATAT | 29554121 | 29554481 |
|  | TGAGTGATAGTTTCACATTCATTTTCAGGA | AGTATTTCCTATTTGACACCAGTTGACAAT | 29508594 | 29508949 |
|  | TCTGCTCATTTTCTCTGTTTTGGAAAATTG | ATCAAGGCATCAAGAAACTTAACAATAAGC | 29687186 | 29687560 |
|  | GCCATTCACACCATGCACATAT | GCCTTGCTGAAGTAATTTTTGCTTC | 29559618 | 29559983 |
|  | GTAGGAGTTATATTTCCTTTCCTTGCAGA | GACTAGACTGTGAACTTTCTGCTCT | 29676019 | 29676393 |
|  | GCTGGACCAGTGGACAGAACTA | TGAAAGATATGCTTTACAACTTGAGAACCA | 29664869 | 29665222 |
|  | TGTCTCTTCTCTTAGCCTTATTTCTCAGT | GTCGTAAGCAAAGCCAGGAAAAACAACAA | 29652647 | 29653018 |
|  | GTTGTTAGGAAATAGGACAGCCACTT | CTTTGCTACACTGACATGGAAAATTTTGAT | 29679179 | 29679551 |
|  | TGTTCCTTTATTCTCTTACAGAAGAGACCA | AGTCCATGCAAGTGTTTTTATTTAAGCATT | 29664365 | 29664732 |
|  | AAAAAGCTCTTGTGAGTTATTGTATGCG | AGAGGCAAACTCCATGAATGTGTTATAG | 29553306 | 29553680 |
|  | GTAAATAGGTAGCCAAAACTTTTGTGTAGG | GGGCCTCCTAAAAGTAGACTGGA | 29683804 | 29684175 |
|  | TGTGGTTGATGCAGTTTTCCTAAAAC | GGTTATATCCAAAGTCCACAGAAAATCACT | 29482889 | 29483263 |
|  | ACAACTTCATTTGTGTTTTCTCCTAGGT | CTGGAAAATTCTGAAATGAAAGGGTTTTCT | 29657288 | 29657598 |
|  | ACAGCTTGGCCAGTAAAATAATGACAT | CCTTGCTTCATGCAGTGTTAGTAAAAC | 29562753 | 29563123 |
|  | GGGTGCACTTACTCTGTGTGTT | GTGCAGTAAAGAATGGCCAGTTATTT | 29556748 | 29557101 |
|  | TTGCTGTTTTAGTGCTTGGCTTAAAA | CAGTGAAGGTCAAATAGGCTGAAGT | 29579748 | 29580122 |
|  | ACTGACAGGCCTGTAAATAAAATCTAGT | AAAAACCTCCTGATGATAAAACAGATATGC | 29663200 | 29663553 |
|  | TGCTATTGACACTTTGATAACTGTTTCTCT | CTTTAGTAATCTCTCACCATTACCATTCCA | 29550302 | 29550653 |
|  | AATTAGGGCCTGAAATGAACCTATATATGG | GCTCTCACCTTAAAGTGTTGGTTGTT | 29527852 | 29528185 |
|  | GGCTCGTTTGGTGAACAAGAGA | CAACTGTTTGACCAGCATCGTTT | 29489912 | 29490254 |
|  | CTTCCAACTCCGGGAGCAAT | CGGAAGTGGGATCCTTTCCA | 29421645 | 29422001 |
|  | AAAATAAGTACTCCAGTGTTATGTTTACCA | TGCTGTGATACTTTTCTTACAGTTAAAACAAGA | 29548786 | 29549135 |
|  | CCTTGCCTAAATTTAATGCTGCCTT | TCTATGGTTCTTTCAGTTTTGTACGGT | 29701335 | 29701707 |
|  | GTGCGACCACATATATCTTAACATTACTGA | AAAAATATCAGCTACCCTAAATGTCACGT | 29701845 | 29702206 |
|  | CTGGTGGTATGGATTATCATGGCAT | ACATTGTTACTGTGTGGATAAATGTGAAAT | 29702435 | 29702808 |
|  | GGAAATACTGAAGACAGGTGCAATTTACTA | GAAGTTTCTTTATTTTGATGGGAAGAGCAA | 29702966 | 29703333 |
|  | ACTTCTGACTTTTGAGGAAAATCTAGCTT | CATCTCCATCTGGGCAACTAAGG | 29703534 | 29703877 |
|  | CACCCTAACATAAGTACTGTTGTTTGGT | CCAAGTAACTTAGCACACCCATTCTTATAA | 29549175 | 29549546 |
|  | GCATAATGAGGCATGTCTTACTCAATGT | CGGATGGAAAGGACAGTAAGTTATCATAT | 29704119 | 29704469 |
|  | AATCCTGCTTCTTTACAGGTTATTGGAA | TGAAGTAAAATGGAGAAAGGAACTGGTAAA | 29663635 | 29663987 |
|  | TGTGTAGTAGGCTATGCTATCTAAGTTCA | CTTAATGCTATTACGTTTGAAACTGCCA | 29700852 | 29701161 |
|  | CACCAAGATTCGGCCAAAAGATG | GAGGTCACAGTGGTTAACCAACA | 29654774 | 29654997 |
|  | AAAAGAAGTTCAGAAAACAGCTTGTTTG | CACTTTCTGTCAGCTGCCTACTT | 29527167 | 29527509 |
|  | CGGCCTTCTATAAGATTCTTGACCT | TTGTTCGCTCTGCTGAAGTTACT | 29654275 | 29654559 |
|  | TTGGGAAGGTTAGAAACACTACCTAAAA | ACTTACACAGGAACTTCATGTAAAACAGG | 29585876 | 29586247 |
|  | CCCAGAAAGTAAAAAGCACTCATCTC | CCCTTATAAAGAATTCTGTAGTCAGTGCA | 29685362 | 29685732 |
| NGF | GACAAAGGTGTGAGTCGTGGTA | ACACTCAGGATCTGGACTTCGA | 115828793 | 115829121 |
|  | CAGACCGGTATCCTTGCAAACT | ACCTCTGAAGGTTTAAAGTCCTTCTCT | 115880725 | 115881099 |
|  | GCCTGTATGCCGATCAGAAAAG | ACTGCCTTTTGACTGCATTTAGTACT | 115829364 | 115829488 |
|  | GCTCCTGTGAGTCCTGTTGAAG | GCGTAATGTCCATGTTGTTCTACAC | 115829060 | 115829421 |
|  | CCCTGCATCTTCCTCAGACTTG | GCATTGACTCAAAGCACTGGAA | 115828471 | 115828845 |
|  | GCTCAGCAGTTGGTTCTGACTT | AAAATCCCAGAACTCAAGGAAATGGA | 115836137 | 115836486 |
| NTF4 | TCCTTAGATCAGCTGGGCCAT | CGCCAGTACTTCTTTGAAACCC | 49564552 | 49564858 |
|  | CGACTCGCTGGTGCAGTT | CTCTAATCCCAGCCTCCCTCTT | 49564986 | 49565299 |
|  | AGTTCACCTCAAAACTGCCACT | GCCTCTATAACCTGTCTATTCTTTTCTTCC | 49566791 | 49567154 |
|  | GTGGTGTCCTCTGAGACTGAGTA | GGAAAATAACAGAGCTGGATGCTGA | 49564263 | 49564609 |
|  | GCCACCTTCCTCAGCGTTATC | CCTGAGTGGGACCTTCTCTCC | 49564805 | 49565146 |
|  | CTCGGAGCACCTGGAGACAGA | TTACGCAAGCCCTAGATATAGGGT | 49565257 | 49565421 |
|  | AGACCTGTCAGCACCTCCT | GTAATCTCTTCCAGCCTCCTTCATC | 49566992 | 49567366 |
| NTRK1 | GGGCGTCAGAGAGTAGGAAG | AGGTGGTGGAGGCTATCCA | 156830538 | 156830911 |
|  | GCACTGAGCAAGCACTGAAAAG | GCGAAGGTCTTCTCACCATCA | 156838109 | 156838455 |
|  | GCTCCCTAGCTTCTCAGTCTCT | CCTCACAGGCATCACTGAAGTA | 156845186 | 156845464 |
|  | CAAACCTAAAGGAGGAAGGCCATT | CCTCTTTTGAGGGAAGAAGAGATGAG | 156841235 | 156841609 |
|  | GGGAGCCAGATGTCAACTTCTT | GTTTCACTGGCATGCACATAGTC | 156836595 | 156836914 |
|  | TCAATGGCTCCGTGCTCAAT | GCTATAGCAGGAAGTTGGCTGTAAC | 156843524 | 156843824 |
|  | CAGAGGGTACAGCTGAACTGAT | CTCAGGCCAGATGGTAGTGATC | 156844250 | 156844530 |
|  | TGCTCATGGTCTTTGAGTATATGCG | GGGATGTCTATAGGGAAGGGAAGA | 156846313 | 156846439 |
|  | CCAGAATGAGGGAGGGCTTACT | GGAAGGACTTGCAGATGGACAA | 156785377 | 156785735 |
|  | GGGTGCAGGTTGAATTTTAGCC | CCCACTCTGTCCTGATGTGATG | 156845730 | 156846078 |
|  | CCTAGTGGGCTTTCTCCTCTGT | CTGAGGGTGAATTAGATACTTTGAGATCAC | 156851210 | 156851548 |
|  | ATGTGGCATGTGCATGTGTATTG | CCTCATTGTTTCCTCAACTACTCTAGAC | 156834047 | 156834402 |
|  | GTGAACCACCGAGCTTGTGTAT | GGGAACCGAGTTCCCTGAAAAA | 156849680 | 156850032 |
|  | GCTTGGCTGATACTGGCATCT | CTGCCTGACCAGCAAACAAGCA | 156830796 | 156831033 |
|  | CTAAATTTTATATGCCTGCTGCCTGATTT | GCTCCAGGAACTCAGTGAAGATG | 156843203 | 156843577 |
|  | CCACATCATCGAGAACCCACAA | CCTGAGAAGAATATGACAGGGCCTAT | 156845421 | 156845617 |
|  | GGGTCTAGAGTAGTTGAGGAAACAAT | CCAGGAGAATGGTGAGGTGACT | 156834373 | 156834730 |
|  | AGAGCAGGGAGATCACTACCAT | AGTCAGGAAGAGCCAGTAGGAAA | 156844499 | 156844873 |
|  | CTCAGGCTCCTGGGAGTTCTAT | GGATCCAGGGTGTCTACAGTTTG | 156848855 | 156849214 |
|  | GAAGGTCCAGGTGCCCAAT | CTCCACAGTGTGTCTCTCTTTCC | 156838310 | 156838524 |
|  | AGGGTCACATGCATCTTCTTCC | CCAGATGTGCTGTTAGTGTCTGTAG | 156844008 | 156844381 |
|  | AGACCTCTGTGTCCTCCCTTT | CCCAGGCCTTTTCAGTGCTT | 156837773 | 156838137 |
|  | GGAGTGGTTAGCCGGAATACTG | AGAGTGACAGGTCCATGGAGTAG | 156851454 | 156851784 |
|  | CCAGGAGCTCCATCACATCAG | GTACCGGAGGAAGCGGTTGA | 156846047 | 156846368 |
|  | GGCTCAATGGACCTCAGAACTC | CCCAATCTTGGCTCTAGAACAACC | 156811711 | 156812080 |
|  | TCACTTCTCAGTGTCAGAGCAGA | CAGGACATCCAGGTAGACAGGA | 156851061 | 156851428 |
| OPRD1 | CCTGGCAATCGCCATCAC | GGGTCTCAAGTTCGTGATCCC | 29139036 | 29139194 |
|  | CCAAGATCTGCGTGTTCCTCTTC | GCTTGAAGTTCTCGTCGAGGAAAG | 29189314 | 29189655 |
|  | CGCGAGCCATGATGTGGA | AAACCGAAGCTGTCTCAAGGTT | 29189864 | 29190167 |
|  | TGTGCTCTCCATCGACTACTACAAT | CCTACACTCACCCTGGTAAGTG | 29185607 | 29185913 |
|  | ACGCCAATAGCAGCCTCAA | CCCAAGCCTCCGACCTTCTA | 29189599 | 29189910 |
|  | CTTAAACAGGGCATCTCCAGGAA | CAGCTCAGTTTTCAAGCAACTGTC | 29190112 | 29190257 |
|  | GGCCAATCCTGCTAGGTCTTTA | GCACACGGTGATGATGAGGATG | 29189043 | 29189372 |
|  | CTTAGGAAAGCTACTTCCCTTCCTT | TCATCATGGTGAGCGTGAAGATG | 29185379 | 29185665 |
|  | CCAGCACGGAGCTCTACCTA | TAGTGGGAGACGTGCGCTG | 54163959 | 54164195 |
|  | CCCTCCACTTTCTACTAAACATTGCC | TGAAGGCAAAGATCATCAATATCTGCA | 54147054 | 54147411 |
|  | CTGCGCATAGAGTTTTCAGCAG | CGGTCTACTCCGTAGTGTTCGT | 54163038 | 54163407 |
|  | TGTTTTTATCCACTAATCACCCACAACA | GCTGTTCGGATGATTCAGTATAAATACGAA | 54138130 | 54138503 |
|  | TCTCATTGATAGGACACACAACCTTTC | GGAGGAGAGTGGAAACACTCTTAG | 54138522 | 54138886 |
|  | GGACCTGTCTTCCAGACCTTCT | GAAGGGAATATCATTTTAGGGTACTGCTTT | 54138969 | 54139340 |
|  | AGGGCAGATAGGAATCTCAATAAAACAAG | GGCTGCATCCTCTGGAATTTAATAGAAA | 54140116 | 54140431 |
|  | CTGGCACCAGGAGGGACATCAT | CAATTTCCTGAGGGCACACTAATG | 54140653 | 54141027 |
|  | GTTCTGGTATAAGTGAACATCAGATTCCTC | ACAAGGGAACAGGTCTTTTCCTTC | 54141270 | 54141599 |
|  | GACTAGTCATACTGGTTTATTCATCCCAT | CTTTCTGGCTCCCGAGAGAAAG | 54141851 | 54142225 |
|  | CGTGAAGTGCTAACTTAAAGGAAACAG | GAGATGAAATCACAGTAGTGTGTTTCCT | 54139510 | 54139884 |
|  | CATAGCTGATCTCTGACTGCATGT | GGCAGGGAAGCCTTGGATTA | 54155025 | 54155399 |
|  | AGGACCAGTCTGGTGATCCTAC | ACGGTAATAACAATAACAGGTTTCTGTGT | 54142170 | 54142443 |
|  | CAGAGATGCCAACAGATGACGA | CCTTATGGGTTTGACTTGAAAATGGAG | 54147352 | 54147726 |
|  | GAACATGACCAGCGAGTTGC | CGGGCAAAGTTTGCCTCTC | 54163352 | 54163694 |
|  | AGCGCACGTCTCCCACTAC | AAATGGCTCAGTCTCAGGTCTTG | 54164178 | 54164482 |
|  | AAGGAAGCTGGGTTATACCGTTTT | CTGGCTGACTTTAAATTGGAACATTGTAAA | 54138439 | 54138589 |
|  | CGAAGGATGCTCTTAGATTGGTAACT | GATCCATTCTCTACATGAACCCTATTCTTT | 54138826 | 54139031 |
|  | AGGAAACACACTACTGTGATTTCATCT | TGATTTCTCCTAATGAAAATGAAGTGCTCT | 54139857 | 54140185 |
|  | TGTAATGATGTCTGTAGTTACATAGGGCTA | AGTAGAATTGTTCCTAAATGTGTGCTGA | 54140363 | 54140710 |
|  | AAAATGTCTCAGCATCAACTTAGCAAC | TGGTAAGAATTACAGCTTACATATGGCTTG | 54140966 | 54141340 |
|  | GCAGATGGGTGCTGAACCTTAC | TACAGTTCAGGATCCTGCTTACCT | 54141543 | 54141914 |
|  | GGCCACTCCAGCCATTCTTA | GGTTTATGTCTTGGATAAGGAAGGCTTATA | 54155362 | 54155577 |
|  | GTATTTTCCCTGCTACGTTGTTTACTTT | CTCCTGACCTCAGAATATTCACCTG | 54139272 | 54139612 |
| OPRM1 | CCCTCTTATTTCTCAAAAGCCAGTCT | CAATAGCTTAAGAAAAGTTAGGGATGGTCA | 154412979 | 154413146 |
|  | AGATATGACCTCCCAGCTATCCTTC | GAACAGAACTCCTCCAATTAGCTTCTT | 154414492 | 154414707 |
|  | TCCCTTTTGTTTTATTTGGGTTTTCACTTT | GAAGGGATCCCTTAAGCAACCA | 154431316 | 154431690 |
|  | TGTAAGGTCTGCTGGGTAGGAA | ACTTTGGGAGTTTCACTTTCTTAATGGA | 154407894 | 154408258 |
|  | TGCAAGTATTGCAGACCAGATCA | GGAGGAGCTTGTCTGAAAATGGAC | 154408321 | 154408683 |
|  | GACATGACTTTGCCTGCATGAA | AGCAAATGCAATGAAAAGGATTTCTTCA | 154331855 | 154332206 |
|  | GCCACTCTGCTCTGCACATTAG | AGCCAGATGCTAATTTTAAGGTGAAGTTA | 154439998 | 154440372 |
|  | CTTTCAGTGGTTTGTTCCTCAGTTTTA | ACATCCAAGTAACTACACAGGATTGAAAAA | 154440576 | 154440950 |
|  | CTTTGTTGTGAAACACAGTAGAGATTTGG | GTTGAGTCCACATAACTATAGCCAATAGA | 154441118 | 154441490 |
|  | AAATAACTGAGGAGGAAAATCCATGCT | AGAGTCTCAAAGATGACATGGAAGAAAC | 154441792 | 154442037 |
|  | ACCCTTGCCTAAGGATGAGATTTC | GCCGTGTCTTAGAAGAGGTTATTTTAAAAC | 154442284 | 154442658 |
|  | TGTTTCCCACATTGTCACAGCA | TGGCGTAAGCTGGAGAAATTTCTAG | 154442800 | 154443170 |
|  | GAAACTCAACAAAGCAGCATCGTT | TTGCAAAGGATGGTTCCAAATGG | 154410740 | 154411098 |
|  | TGCCCTTGTAGAAACATCAAGATTCT | CAGTTTGTAATGTACACAATTCCTTGTACT | 154443419 | 154443792 |
|  | CATTAAAACCTGCACTTGAAATTGCAC | CCTTGCCCTTTATCATTTCTACTTGATTCT | 154445543 | 154445917 |
|  | CAGTGCTCTGTCTCAGTGGTAA | CCAGCCCATTCAGAAGTTATATGATTGAAT | 154449025 | 154449316 |
|  | CTGAAGGACCATTTATTTGAAGCAATGT | TCCCATTTATCAGACCTTCAGCTCT | 154449544 | 154449918 |
|  | TCTGTGTTAGGCATAGCTATATGAATATTTGC | CACTCATTGTTTTACATTTTCATAACTGGCTAT | 154451686 | 154452060 |
|  | ACTCTGTCAAACATAGCAAATAGGAAAGT | GGCAGGAGTCTGTTTAAAATGTCTGAA | 154452143 | 154452457 |
|  | TGTATGCCAGGGTGACATTTTAATTTACA | CATCAAAAGGAAAACAATGTTGTGTGTT | 154452689 | 154453049 |
|  | AGGATGATCAGATGCAGCTTTCA | CCTACGTCGCAACATTTACTTGG | 154453201 | 154453575 |
|  | GACCAGTTAGGTGTTTTCAGAAACAC | TTCTGACTGGCTCTAAGTCAAAATGA | 154448478 | 154448821 |
|  | CAATATAGACCTCATGGAGGATCTAGCT | GTTTTCCCAGTACCAGGTTGGAT | 154411788 | 154412139 |
|  | TTCCAGACTGTTTCTTGGCACT | CCTCTTCTCTAACACTAGAACCACAATT | 154412386 | 154412755 |
|  | CCAAGCACAAGCAGCTACATTAG | CATGAAATAGCTTTGGATGACAACAAATCA | 154446143 | 154446461 |
|  | ACAACTCTTTTCCTAAGAGTCTGGGTA | TGAGACATTTGAATTTTGCAAATATTGGGT | 154447355 | 154447639 |
|  | TGAGCCTCTGTGAACTACTAAGGT | GTGGGACAAGTTGACCCAGGAA | 154360445 | 154360787 |
|  | CAGAGCAAAACACATGTGATAAAACATAGG | GTTTTTCTCTCTCTCCTTTTCCTTTCCT | 154429317 | 154429519 |
|  | GAAGGTACTGCCGTGTGGTTAA | CAAACGGTTGAATGAATGGCAATAAAATAA | 154428807 | 154429145 |
|  | GCAGCAACACTGTAGAAGTTCAG | AGCATTCCTCCATGGATTGCAA | 154429582 | 154429953 |
|  | CTTTGGGTATATGCTGGAGGCAAT | GTGAAAGGAAACGTGGTTATGCATA | 154407317 | 154407685 |
|  | CCACATCCTTTTATCAGTAAGATCTTCGT | AATATTGTGTGGAAAGTACCTTGCTACA | 154445020 | 154445300 |
|  | TTAGAGAGAGAGCACAGAGTCCCT | AAAAATGTCTAGCCTTAGTGGGC | 154446644 | 154446929 |
|  | AATTATTTTGTCTCTACCCAAACCATCGA | TATGAAATTTTAAACATTTCTGGCCAGGC | 154451253 | 154451623 |
|  | GCTTCTATGCTGGGCCTTACTT | AAGTTGCCCTATGCGGTCTT | 154444490 | 154444861 |
|  | GGTAAACATGTGGGTCCTGCT | GCATGGCATGTGGCACTTTTT | 154447900 | 154448271 |
|  | ACTGTTAAGAGTTTGAATGTCCGCTT | GAAATAGTTACAAGCCTTTGCAAACTCAA | 154428367 | 154428737 |
|  | GGCTCTATTATGTCAAAAGAGAATAGGAGTTTT | TCTGTTCTCTATTGTATAGAAGAACTCCCTG | 154450065 | 154450422 |
|  | AGAGGGTCTTTCTCTCCTCTCAATG | GAGGGTGTCGGTGGTAAAGTAC | 154450612 | 154450978 |
|  | CCTCATGCAAGAAAGAATTCAGGAGTAAA | TGCATAGATAGAGGCAGTTGTACAGA | 154444009 | 154444328 |
|  | CCTGGCAATACATTTCCTGAACTTT | GTGACAGAGCACCTAATCTTTAGACTT | 154451992 | 154452209 |
|  | CCCTTCCAGAGTGTGAATTACCTAA | ATCACCAACATATCAGGCTGTGAA | 154411040 | 154411370 |
|  | AGGGCTGTTAGGGTTTCATCAAG | CATCTGACATTCTCCTCTGCGTAT | 154360132 | 154360503 |
|  | CAGCCAGGACTGGTTTCTGTAA | GTAGAGGGCCATGATCGTGATG | 154360543 | 154360910 |
|  | TCTCTCCTCAAGTTAAATGGCTCTATCTT | TAGGACAGCTTTGTTACTGAAGCTTTAAA | 154408191 | 154408383 |
|  | CAAAAACAAAATTGTCTTTGGCTCAACA | CTTGACCGAATTCTCTTGTTTGTAGGA | 154331546 | 154331914 |
|  | CCATTATAGAGGATGAGAATGGAGGGAA | CACTCACCCTCTCTCTTTAATCACA | 154412689 | 154413040 |
|  | CTGAGGCTTGCAGGTGAAAGTA | TTCCTTCCAAATGCTCCACTTACTTT | 154439689 | 154440056 |
|  | GTTTTGCAAGGGAATGAATCCATTATTCTA | CTTTTGTGAACACTTAAATAGGAGCAAGAA | 154440303 | 154440643 |
|  | AAGACAGACTCATATGACCATACAGAAAAG | ACTTGGCTGTCTACCTAGATCAAGTAAA | 154440880 | 154441185 |
|  | ATTTCCCTTCCCAGGAAGAGTCTA | GTCGTTTTTCTGTGTTGAGGGATACT | 154428674 | 154428998 |
|  | AACAGCTATCATGGATCAGAATAACTGAAT | CCATTTTACTTTTGGAAGCTCTAGTAGTGT | 154441421 | 154441755 |
|  | CCTGATCTATCTTTTTCCACAAATGTCATG | GCTACAGTCTGCATTTCTGGGATAC | 154441969 | 154442343 |
|  | GCCATTTGAAACACTTCTCAACATTGAAAT | CAACTTTTGCCTGGATCCTGAGA | 154442588 | 154442855 |
|  | ATTCTCCATCTACTGGCAATTACCAAG | GGCCTGTGAGTAAAGACTTATGTTGA | 154443108 | 154443481 |
|  | AGGAAAAACAGATTGACAGTGGACT | CCTATGGAAAACAACTTCTTCTGTGATTTT | 154445237 | 154445610 |
|  | GTGCAGAAGCTTTATTGTTTATTTGGGA | GACAGAAGTTTTCCTGAAATACAGAGC | 154445849 | 154446203 |
|  | CCCTCGCTTTCACTAAATAACAACTC | CAAATGAGTTTTGAGTTAATACAGCAGTGA | 154448759 | 154449087 |
|  | AATCTGGCAAGTGTTCTCAGGATC | CCCAGTTCTTAGACTTCACTGGAATTT | 154449248 | 154449609 |
|  | AGAATCTGAGTAAATGAGAGCCTCTCAT | CAGTTGACATTTCTATGTCCTGTTTAGGT | 154452392 | 154452757 |
|  | GTTTGTTGTGATAATGCACAAAAAGGAA | TGCACATGAGACCATTATGATTTAGCA | 154452983 | 154453261 |
|  | CCCATTTCAAGAAGGGCAGAAGT | GGGAAAGTAAGAAGTCTAATATGTCACC | 154447576 | 154447948 |
|  | TTTTTGATAAATTCACAGGGTTACAAAATACC | ACTCATGTTGTTCAGTGAATATGGCT | 154447248 | 154447418 |
|  | CTTTCTTCTAGGTTCCATAGATTGTACACT | AGGCAGCTGTTTGTGTAACCTAG | 154412076 | 154412441 |
|  | TTGATGGTACCTGAATTTGCCTCTATC | GCATGTGACAATATGAAGATGAATTTTCCC | 154446394 | 154446708 |
|  | AAGTTTCTCTTATCAGTCAGGCACTTT | TGAAATCGATGGTTTGGGTAGAGAC | 154450919 | 154451286 |
|  | ATAGGAGGAAGAAAGCAGACTCACCAT | TTTTTGCATTGGTTTTGCATAGGTTTT | 154567701 | 154568074 |
|  | CTAATGCTAAGCAGCCCTCCTAT | TGGGAGCAGTATTTCCACGAAG | 154414179 | 154414549 |
|  | ACACCAGCTTAAAAATAGCCTTTGAATT | GCTAGTCTGATATTTGGTGCTAAGCA | 154429014 | 154429384 |
|  | GGCAAAACAGTTGCCCTGAGTA | AGCTTTGATGCTATGGGATATTAATTGCTA | 154429459 | 154429802 |
|  | CCGCTTTATAAACTTTTAATTGACTCCCAT | GCTAACATTTCTTTGGTTCTAGACACTAGA | 154429894 | 154430213 |
|  | ATACACATTGTGGAATGGCTAGCA | CTGCATGATCTAAAGACATGCTTCATTG | 154407626 | 154407954 |
|  | TCTCACGCTCTCAAATCCACATC | CAGAAGTATACCTTGTAAACATTGGACATC | 154408626 | 154409000 |
|  | ACATCATTGAGGAAAAAGGCTACCTT | CTACACCATGAGGTTCCTATACTTCAAAAT | 154449857 | 154450151 |
|  | CACTAGATTTCACATGAAAGGGTCGT | TGTGGAAGTCTCCAATCATATATGATGTTG | 154450293 | 154450667 |
|  | CTCTGCAGCTATTTGAGTAACTTCTCTTAT | CTGGAGAAAACTCAGACTATAACTGCT | 154444262 | 154444549 |
|  | CCCTTTTAAGACCGCATAGGGC | GTAATTCAAGTGTCAGAGGTGTTTGAAC | 154444835 | 154445201 |
|  | AGTGCCACATGCCATGCTAT | CCTAAACCCTTGAAGAGTAGGCAA | 154448255 | 154448538 |
|  | CGCCTGGCCAGAAATGTTTAAAAT | CCCATTAAAATAATGGAAATGTATGTGCCAATA | 154451594 | 154451950 |
|  | GTTGTGGATGTTTATGTTCTGCCTT | GCCACTACATTTTGGAATGATTTGTAATGT | 154415238 | 154415574 |
|  | CATAGTCAGTGTTCTTCACTGTCTTCA | TGAGACCATTGATCCTGACCCAT | 154443725 | 154444071 |
| OXT | CCCAGAAATGGTGAAAATAAAATAAAGCAG | ATCCTCGTCTTTTTGAATTTTGAAAGATCC | 3053120 | 3053467 |
|  | CAGGAGCTGAGCGGATTTTGA | GCGACGGCAGGTAGTTCTC | 3052514 | 3052852 |
|  | CTGCCAGGAGGAGAACTACCT | GCACTTAGACACGAGTCAAGGTAG | 3052824 | 3053184 |
|  | GCGACCCTCTGTGACCAAT | GCTCAGCTCCTGGAGTTCTCA | 3052171 | 3052525 |
| P2RX7 | TCATTGGAGGAGCTTGAAGTTAAAGAC | GATTCGAAAACCATGTGAGATTCAGAATG | 121570632 | 121571006 |
|  | GGTGAGATCCAGGAGATGATTCTTT | TGGCAATTCTGCTTTCATTTTCCC | 121592920 | 121593294 |
|  | AAAACCCAAAACCCAGCACTTTC | CCACAGCAGTAATTAGGACACAGT | 121613129 | 121613345 |
|  | GCTCACTCCTGGGAAAGAGACA | AGTAAATGTGAGCCACATTATGAAGACC | 121603785 | 121604101 |
|  | GCGATTCTGAGTAGTGCTGCTATAAG | CTATGGCTTGGTGATGGTGGAA | 121598583 | 121598906 |
|  | AGTTAATGATGTCCCTCCTGGAGAA | CCCAGAGATACTAGTTTGTGCCAA | 121600146 | 121600496 |
|  | ACATTATCCAGCTGGTTGTGTACAT | CATTTCTGAGTGATTTCATGGAATCTCTC | 121614963 | 121615313 |
|  | GCAGTTCCTCCTGCTCTACCA | TGCCTGGCTTCAGTAAGGAC | 121622386 | 121622614 |
|  | CTAACAAGGCGAAATCCTGTCTGTA | CACTTTGCTCTCAGACACAGAGA | 121622699 | 121623051 |
|  | AATGTCCAACTAAAAACAAACCCTTTTCAT | TCTGGTCTTGGACTCCTGACTTTAA | 121623302 | 121623665 |
|  | CTAGCTATCAAGAGGCTGAGACAG | GCAATGTGGTTTTCTGGATTTATGGG | 121623741 | 121624097 |
|  | GTTAAGGCATGAAAATTGTCTGAACCT | CCTGGACAAATCTGTGAAGTCCATC | 121621766 | 121622140 |
|  | GCTGCTCGTCCAGCTTTGATAT | CAAAAGAGGCAGGTGCTCAGTA | 121593710 | 121594083 |
|  | GGGCTGTACATATGGTTCTTCAATCA | TTTTTCATTCATCTTGTTGCCTTGGAA | 121605155 | 121605529 |
|  | GATCTCAGTCCTTTTCTGAGGCAT | GTAACTGCAAAATGAAACGTACTGGT | 121570350 | 121570695 |
|  | CAGCAGAGCTAGGATTGGAACAG | TTTTTCTGGAGTGAGGCTGTAGATC | 121592483 | 121592853 |
|  | TCTTGGTTAATGAGAGTTTTGAGCCA | TCTGAGTGCCGGTTATATGAGTATGT | 121618019 | 121618393 |
|  | GGGCTTTGTGGAGAGTTTCAGA | ACAGAAACCGTGGGAGACAATAG | 121602989 | 121603351 |
|  | TCTGAAACCCTCACCTTGTATGGA | CTCTTACCAGACCGAAGTAGGAGA | 121614648 | 121615022 |
|  | GGCTAATAGGTTTGGAAACTTGCTTTTT | CTGTTGGTGGAATCCACATCCA | 121622077 | 121622445 |
|  | GAAGGGCAGTACAGTGGCTT | CCCTGCTATTGGTAAGGACTCTTTTT | 121622570 | 121622919 |
|  | TCAGCCAAAGGGAAATATGCTTTCA | GGCCACCTTTAGTTCTCCACATT | 121622993 | 121623365 |
|  | GGAAGCCAGACGCCATTTAAAA | CAGTCTCAGGATAGGCAGACATTTTT | 121623542 | 121623902 |
|  | CTGATACCGCATGATACATCCCAA | CGGCCTTCAGTCAAAGAATTGT | 121624037 | 121624395 |
|  | CCCTTTGAGAGGCCATGTTCTA | CGTAGAACCACAGATGTGAAAGAACTG | 121599888 | 121600208 |
| PLCB1 | CAGGGCAAGATAGGAGTTTCAATATAGC | CTAACTCCCAGCGTGTGACTAC | 8639051 | 8639422 |
|  | TGTTTCCAACCTAATTCAATTGAGAAGTGA | TTTAGGGCATACTGGCTATTGTGTT | 8351858 | 8352232 |
|  | GAAATTACATTGAGGATTATTTGCCCTCAA | CCCAGCATTGTATATGATACCTGAAACTAT | 8707824 | 8708197 |
|  | AGATCCAGGATGATAAAAACCAATCCTG | TATCCTCCTTGTCAAAGATTTCATCACC | 8696769 | 8697137 |
|  | CAGCTCTTAGACTCTTTGTTTTCCTG | GCCTTTGGACTTAATTGTGACCACT | 8678164 | 8678538 |
|  | TGAAAAGAGATTTAGATTCAAGGCCCAAA | GCTACAAAGTATGGTTACTTCTCTGTGATT | 8740950 | 8741307 |
|  | GCACGCCTTGCAACTCAAG | TTGGCAGATGCCAACCTGT | 8113322 | 8113545 |
|  | TACTTGTAAGTGTAGTTCTTGATCAGGAGA | ACATGAGTTTCATATGCAAGAACTGATCT | 8770683 | 8771037 |
|  | GTTTCCTGCATTTCTACAATGACTCTC | TGACTAAGCCACTTCTTTATGCAATGT | 8713744 | 8714118 |
|  | AAAAACCTTTGGCATGCACATTG | CCCAAAGGAAAATTTGTGTTTATCAATGTG | 8626633 | 8626954 |
|  | TCAGTTAAAGAAGCTCAAAGAAATCTGTG | CAAAAGAATCTTTTGAAAGGCTGACTAGA | 8769140 | 8769509 |
|  | TTTGCACTTTGAAAGAAAGGCGTT | CAGTTTTGTGTAAGTCAAGGTTATTCAACT | 8864494 | 8864714 |
|  | TTTAAACAGCCCAACTTCCAAGGA | GGCAATTGGAAGGCACAAATCTAC | 8702881 | 8703188 |
|  | CCTAAAATCCACACCAAAGGGAGA | CATGTTGACTTGAAGATAATGGTAGTTGAG | 8862606 | 8862978 |
|  | GTACATTTGGCTGCATGAGCAA | GCACTTACATGACTAAGTGGGTACAT | 8863185 | 8863533 |
|  | GAGACGCTAAGAAATAATTGATGGAGC | TTTTTCAGAACTTTCAATTATGCTTTTGATCA | 8863686 | 8864048 |
|  | TGTAACTTAGAGAGTAACACATGAACATTGAAG | CCCTTACGTCTTACTAGATGTGTTTTT | 8864074 | 8864373 |
|  | GGGTCTGTATGATGTGCAGTTTTG | TATTAACACAGTGCACAAGCGCAACAT | 8864937 | 8865304 |
|  | CGGAGCTCGAGCCCAAATT | GCCTCAAAGCGCACCATTC | 8112598 | 8112966 |
|  | GTGGGTAGATGGGTGATGAATGAA | CATGAAAGTGTTGTTCCAATGTGCTATT | 8721990 | 8722345 |
|  | ACAGAGAACTTTCTCTAGGAGGAATTCT | CCACCCTTGTCTCCTTTTAGTTATCC | 8755089 | 8755461 |
|  | GCAAATGCATGGAGGAATGGTT | ATTCATTAACCCATTAATCATGGAAGCTCT | 8629868 | 8630238 |
|  | AAAGAAAGAGAAAAGAAAAGCTAGTCCTGA | GCCCTTGTTACATAACAAAATTACAAAGCA | 8719802 | 8720133 |
|  | TGTGCATTTGTAGATACCGCTATTCA | TGCTGTCTTCACTGATCTTTCCTTTC | 8862003 | 8862377 |
|  | CCTAAGAAGGTAGCCGCTCTTCT | AATCCATGTTAACAACTCTTCATGCTATGA | 8745706 | 8746064 |
|  | CTAAAATGTCTTCTACCTTGGACACTACAA | CTTCGCAGGAAATGGAAGAAAACA | 8665462 | 8665835 |
|  | CCTCCCTTTTATAAAATGAGGTGTTGCT | CTATTAAGGGTAACATTCTTTGGTCCCAT | 8705119 | 8705493 |
|  | AGGCAAACAGAGGAGTTAAAACTGT | ATGGGAATGTAGAGAAAGGGAGGAT | 8782566 | 8782936 |
|  | AGTAACCTGAAATACATGCTTTCATCACA | TTGTTTGGTTCAGTTTTCCTTTCACTAAC | 8720892 | 8721194 |
|  | GCCAATGTTAGATGTCTGACAACTTTAAC | TCCTGCCTCTTTTTATCCATTTTCTTCTTT | 8768950 | 8769324 |
|  | CTCAAAATCCCTTCTAGCCATTTCAG | CACCTACAAATGAAAACATGCCAGTTAA | 8689172 | 8689546 |
|  | ATTGACTACTTTGGAGAATCTGTACAATT | GCAAAAGATCTGTGAAATGGATGTTCT | 8130833 | 8131124 |
|  | TTCTACTCTTGCTTCTATTCCATTTTGCT | TTCTCAGTGTAAAACACCTGAAAAATGC | 8717533 | 8717900 |
|  | TATCCCAAAGTAGCCCAACGAGAT | TTTTTCAGTGAAGTCTGGCAATTTTCT | 8709560 | 8709928 |
|  | ATTTCCAACTCCAAAAGAATTGTGAAGA | CCTCCTAAGTCCACACATCTTTCC | 8737578 | 8737951 |
|  | AAAAAGGGCCATTTGCTTGCATTA | TTTCCCTGTCAAAAGAAGGTTGACT | 8865243 | 8865597 |
|  | AGATGGAATAAGAGAAACAAATTATATCAAGGT | GTAAAATAAAACTGTGAGCATCTGGCTT | 8863979 | 8864145 |
|  | AGGCTTAGTTCAAACATAAGGAAACAGT | GCATTCGATTTTGACCATTCTTGCT | 8864308 | 8864553 |
|  | CCCTCGAGATTTTGGAATTCGTG | GGGCATGGGAATTCTTCATGGAT | 8862318 | 8862663 |
|  | GCAGAACATTGTATCACATTTTAGTCCAG | TTCACACATTCGACAAAGAACAAGGCAAAT | 8864645 | 8865001 |
|  | GCCGACTTTGAGCATTGTTTCT | AAACAGCACACTGTCTGGAAGT | 8862916 | 8863239 |
|  | ACAAAGACCCACATGTAAATGATAGGTATTATC | ATAAGTTCTAAATCTACTTCGGTTTGCATCA | 8863464 | 8863746 |
|  | CTCGGCTTCTCTTCGCCTT | CCTTCTTGAGGCTGTCGGA | 8113019 | 8113371 |
|  | ACCTCCTAGAAGAAAAGGAAAAAGAAAGG | GACACAGTGTATGATGTTCTTCCATCT | 8637669 | 8638042 |
|  | TTTCTTAATCTGGGTGCCAACTGT | CTGCACTGACATGAGACATGTG | 8628391 | 8628755 |
|  | GAGCAGTGTTGAAGGTAGGGAA | AGCCCAGAGCAGCAATTCTTAA | 8698194 | 8698559 |
|  | GAATGGGAGCCTTAAACTCCTACA | ACAAAAATTCAGTCTTTTGCAGGCAT | 8608793 | 8609167 |
|  | AGAAAAAGAATGTTGAGAGTTCTGTAACCT | CTCTGCTAGGTGCTAGTGATAAAAAGTTA | 8770057 | 8770347 |
| PRKCG | GTCAGAGGTCGGAGACCGACAA | GAGACCACACACAGATGGAGATG | 54395626 | 54395963 |
|  | GGTCTGGGTTCCGGGAAA | GGGCGTGACCATAGAAAGAGG | 54393045 | 54393398 |
|  | TCAGTCCCTTAAGAGATGGAGGAA | GATGTCCAGAACTGGATAAGGCA | 54401570 | 54401942 |
|  | GGAGACTTGATGTACCACATTCAACA | GCATCCAGCATCACATTGTCCA | 54403523 | 54403897 |
|  | AGAGCTTGTGCTGAAAGCACTTA | AGAACCATGGACAGCGCTATTC | 54406214 | 54406531 |
|  | GTCACCCTCCAGGCAACAAAAA | CGTAGGTGAAGCCCTGGAAATC | 54409716 | 54410081 |
|  | GAGCGCCTTTAAGCCGAAAC | GGAGCTGGAGACTCGAAGGAT | 54385382 | 54385684 |
|  | CCTGTGCCCGTCATGTAATCTC | GATGGTTAGTGGTGTGGTCTCT | 54410132 | 54410442 |
|  | GGGATTTCTGGGATATATGGAGGATTCTT | CTGTTTGTGTCTATGTACAGTCACTGT | 54410652 | 54410986 |
|  | CTGGGTCTGAAGGAGGAAGAAAC | GACCAGAGAACACGTGTGTCAA | 54386197 | 54386541 |
|  | TGTATCCCTCCATTAGGAAGTAAACTCC | CACTCACAGGAGCATTTCATGC | 54392637 | 54393010 |
|  | GCTGTTCTTATCTCTCCGGATCTCA | GTCAATCCAGCGGAAAAAGCCAT | 54409434 | 54409660 |
|  | GACTACAGTTCCCAGAAGACCCTA | GGATCCTGGATTATTCAGCAAGCA | 54396426 | 54396798 |
|  | GGAATTTCCCTGTGGCTCCTTT | GGTCGGCGTGATAGGAGTCT | 54385641 | 54385986 |
|  | GGTGGCCATTTTCCTCTGTCTA | CCCACTTCCTCCCTAAGAACCA | 54401085 | 54401444 |
|  | GATGTGGCTAGGTGCTCTGAAT | GATGCCCTGATTGTGAAGGAAGA | 54403353 | 54403727 |
|  | CCAGGAATTTCCGTGGAGGAAA | GCCACAACTCATGACAAGGTCT | 54403746 | 54404119 |
|  | CCCTCGGAGCTGCTTAACTTTC | GTGCAGGATCTAGAATGGGACA | 54409918 | 54410256 |
|  | TGACCTTAGCGTTCTGGACTCT | GAAGTCTAGAACAAAAGCTGATTGGGA | 54410384 | 54410718 |
|  | ACACAAGTTCCGCCTGCATA | GTTACGTGGATCTCATCTGCTGT | 54392906 | 54393269 |
|  | GGCCTCCGATTTTCTCTCTGTT | CCCTCCCAGCCATAAAAATAGCA | 54387251 | 54387599 |
|  | CCCTCTCCCTCTCTTTTTATCTCACT | GAGTCCTAGGGTCTTCTGGGAA | 54396095 | 54396455 |
|  | TGGTTTGGTCTGATCTCTCCTGA | AGATGGAAGTAAAGTGACTTCAGGAATG | 54407734 | 54408108 |
|  | GGCCAAGCTTGGAACTCTTGAT | TCTGGGTTCCCTCTATCCTAACC | 54394832 | 54395195 |
|  | CTCTCTCTGTGTTTCCCACAGTT | GTGTCTGCACCTCCTTTTGTTG | 54409550 | 54409845 |
| PRNP | GTCAGTGGAACAAGCCGAGTAA | GGGCCTGTAGTACACTTGGTTG | 4680156 | 4680361 |
|  | TGAACTAAAAGTCATTCATCAAGTCCAT | ACTCCATGTGGCCACAAAGAG | 4679610 | 4679917 |
|  | CTGATGCAAGTGTTCAAGCGAA | GCGTTTACCTGCCTCGGTC | 4666863 | 4667166 |
|  | GATCTCTTTCCTCATCTTCCTGATAGTG | GCAAAGGTATTTCAGACTGTTCTGAGAT | 4680595 | 4680969 |
|  | TTTTACTTTTCACAGTATGGGCTACACA | TCTATGATGATGGTGCTTTCACAACT | 4681211 | 4681572 |
|  | TCCCTGAATTGTTTGATATTGTCACCTAG | GGTTTCCCACATATTAAGTATTCAGTACCT | 4681811 | 4682031 |
|  | AGCAGTCATTATGGCGAACCTT | GCCATGTGCTTCATGTTGGTTT | 4679857 | 4680204 |
|  | AGGACCGTTACTATCGTGAAAACATG | AGATTAGAAAGATGGTGAAAACAGGAAGAC | 4680303 | 4680663 |
|  | ACAAGGAGTAGAAAGTGAATGAAGGAAG | CGGGAATGAGTCACCGGAAAAA | 4666545 | 4666917 |
|  | CGCGAGCTTCTCCTCTCCT | CGCGCAGTAAAGGGAGCTT | 4667126 | 4667408 |
|  | TTTGGACTTAGTGCAACAGGTTGA | GTTCAGTGTTGTGACAATATTTACTCTTGT | 4680907 | 4681279 |
|  | CCTGGAAACCAGAATGATTTTGACATAC | GCAAGCCAATAATAACATTGCAGAAAAGTA | 4681508 | 4681880 |
|  | GCAGTTAACATCTGAAGTGTCTAATGCAT | GCCACTTGGCTCAGATTAACCC | 4681962 | 4682314 |
| PTN | GCCAAAGTGAAAGAATTTTATGTCTTAATGCTT | AGTACATGATAAATGATAGACAATTGAGGTAAG | 136912176 | 136912536 |
|  | CGGATGACTCACTGGTCTCTTT | TTGGTTCAAATCCGTTTTCTTGCTC | 137028318 | 137028630 |
|  | TTGAAAAGGGAAGATTGAAGGAATAGAGG | TCCTGAAATCTAACATTCCAGAAATGGA | 136939469 | 136939838 |
|  | CCAACCAGGGATGATTTTGACCA | GCAACTGGAAGAAGCAATTTGGC | 136937886 | 136938234 |
|  | GAATACAAAGCCTACGGTACATATAAATGC | CCCTGCAATTTAGTAACTTGACATTTTTCT | 136912680 | 136912889 |
|  | TGCCCAAGAGTGACTTAGCTAGT | GCATGTCCTAGTTCAATGTTGACTTTTT | 136911967 | 136912250 |
|  | AGTCATAACATTTAAATTGCTGCCCAAAA | CATAAAAAGGACATCAGCAAACAGGATC | 136912430 | 136912748 |
|  | GGGAGTTTTTCAGTCACTTTCTTAAGG | ACAAACAAAAGTGTGCCTTTATTATCCTG | 136935852 | 136936220 |
|  | GCCTCTGGAGCAAGTCAGTTTT | GAGCAGAGGAAAATCCAAAGTGGA | 137028020 | 137028374 |
|  | CTCAGAGTAGGAGATTAACTCAGTAGCA | CAGGAATGAACTTGGTTATATCTGGAAGTT | 136938173 | 136938459 |
| PTPRZ1 | CCAGGTAAGGCATTATTTCACTGCA | CCCAAACAGCCACACTAAGGTC | 121674166 | 121674540 |
|  | AATTAGCTTACAGATCAGAGACACTTCAAC | TGGGCTTGCAGTCAACAGATTT | 121691769 | 121692131 |
|  | TCAAGTCCTCAAGGCTAAGTTCTATCA | ACAACCATTTGTAAGTTCTTACCTTGGA | 121636383 | 121636697 |
|  | CCATCAAATTTCTCCAACTTTGCAAGT | ACTTTGGCTCCTTCCTAATGCTG | 121678663 | 121679037 |
|  | AAGGCACGAGAAAGATGTTAAGAGTAAA | AGGACAGATCCTTAGGTCAGAATATGTT | 121694843 | 121695217 |
|  | CTGCAAAGTTGTCAAGGCTATATGC | TGGACTTGTAATTCAGCTTGAAAACTACAT | 121699711 | 121700084 |
|  | GTAAGTAACAGCAATGAAGGTTGCA | GCAGCTCTCAGGTAAAATACATTTTGAATA | 121659079 | 121659434 |
|  | ATTGGTAATTGTTGCTTAACAATTGAATGC | TACTAGAAGTGCAAATGGTTGCACT | 121671379 | 121671726 |
|  | AAGCTCATGTAGTGTTGTGTTGTAGT | CCCACAAACAGTGAATCATACACAC | 121644466 | 121644835 |
|  | CAAGCTTGCATAAAAGTTAGTTCTCATTGA | GGCTATTACATGTTGGATATTTCTTTCCC | 121607681 | 121608055 |
|  | TCCTGACTTGCTGTTGAAGAACC | CAAGAAATCATTCCCATTTGGGCAA | 121680761 | 121681134 |
|  | CCCTTCCTTATTGAAGGATGGTAAATGA | CCACCAGTTTTAATTTTACTTGTGAGCT | 121616074 | 121616445 |
|  | TACATAACAAAGCATGTGTCTCTGTTTCA | AAAGGCATTGGTAAATGCTTTAAAATGGAT | 121623623 | 121623983 |
|  | TCTGTAAAACTCTCCTTGTCTGTCAATG | GGCTCAAATACAAAGTCATTGTACCCA | 121684372 | 121684745 |
|  | CCCTTTGCTCTGTCTTCTTATTACCA | CGGTAGTCATTAGTGAGATTAATTTCCACT | 121612250 | 121612622 |
|  | TGACAGTAACTTTCATGACATAGGATTCTG | TTCTACTTTAGGTGAATACTGCAGTTGTAA | 121701379 | 121701743 |
|  | CTACTGAGTCAAGTTTTCTAGTTCTGTGT | GACCCACATCCACAATTTTCTGC | 121701827 | 121702153 |
|  | TGTTTGCTTTTCAAAGGAGGAGGAA | GAGATCTAAGAACAGTTTTAGAGCCATCAT | 121650372 | 121650745 |
|  | AGAATCTGCTAGAAATGCTTCCGAA | GTCTGCGAGTATACCACGTTGA | 121650972 | 121651343 |
|  | CTTCAGTAGTGAATTGTTTCGCCAT | TGCACAGGTATTGCAGAACTGTAA | 121651578 | 121651952 |
|  | TGGTGATGATAATAAGGCGCTTTCTAAA | TTTGCACTAAGCACAGGTTTAAGC | 121652202 | 121652540 |
|  | AGTGAAAACATGCTGCACTCTACA | AGCAACTGTTGGAATACCAGCA | 121652794 | 121653168 |
|  | CATTCATAAGTGTATGTCATGCTCATCCT | CACTACCAGTCTGAATGTCATTTTCC | 121653414 | 121653685 |
|  | AGATTATTCCTCTCTCGCTGTCTCT | CTGAAATGCTCCCACCCTCAT | 121513318 | 121513678 |
|  | CTTCTTTTCCCTAGGAGCACTGAAT | ACTAGTCTAAACATACTCCAGGTCTGAAAA | 121607991 | 121608249 |
|  | GAAATGTTCCTGAGGGTGGAGATT | GCACTAGTATATGTATGCATGTATGGCA | 121676452 | 121676826 |
|  | TCATGCCTGCCATTGGGTTTTA | ACCATGGCTGAATCATCACAGTG | 121698742 | 121699112 |
|  | AACATCTTGTGACTTGTTAAAATGGATGT | AGCAATTTGCATAGTGTCCCGATA | 121668437 | 121668795 |
|  | TCTGAAAGCCAGGTAATCTTAGAAATTCAA | TTTACCAAAATTACCTCCTATAACACTGCA | 121623865 | 121624239 |
|  | TGATTTGCATGCAACTGTTTGAAACA | ACAGCTCTGCACTTCCTGTAAAAA | 121673977 | 121674350 |
|  | TTTGTTTGCTTTATTTGTTTTCACCTAGCT | AAGTGTGGAAAGTGAGAGATTGCT | 121616674 | 121617047 |
|  | TATTTTCCACGGACCAGGGAGAA | ACTTTGCCTTTTGGCCAAAAGAATAT | 121637829 | 121638168 |
|  | GATTTTCAGAAGCTTAGGTGCAATTGTA | CAAGTGGCAAATCAAAGATAAGTCTTACAT | 121612554 | 121612928 |
|  | ATGTCTTCACTGAGCTTCGCAT | TGCAAAAAGGCACACATTGTTAATGATAT | 121701084 | 121701448 |
|  | CAGTAGCCTGTAAATAAAACACTCTTCCAT | GTAAGACCAGCTAATGAACTACGTCATTAA | 121701669 | 121701896 |
|  | CCTCACACTGTGGAAGGTACTTC | GATCCTTTAGTGATTCTTCTGAACCTGAT | 121650682 | 121651036 |
|  | CCATCCTCCAGACAACAGGATTT | TGAAGTAACTTGTGGAAGGATTTGAGAA | 121651288 | 121651641 |
|  | ATAATGAGGGCTCCCAACACATC | GAATTTGCAGTTCAGTCTCATTTCCATAT | 121651895 | 121652269 |
|  | CTACACATACTGTCTCTCAAGCATCT | ATGAGAAGTAGGCGACACATCAAAA | 121652480 | 121652853 |
|  | TGGGCTATCCCAAAAGCACAAT | TTTTAGAGCAAGCAAACCACCACA | 121653627 | 121653998 |
|  | TTTAACCTCCACCAAAAGTTCTGTTACT | GTGTCTGAATCATTCATTACCTTTTCCTG | 121653108 | 121653482 |
|  | GCCCAGTAAGCATTGGCTATAATAATCA | TGCTTGTTTGTATGCTGGACTTTTTG | 121693804 | 121694178 |
|  | AGGAGTCCATTCCTGTCACCAT | GCCTGGTTACAAGAATTTTTAATGCCTTTA | 121679403 | 121679773 |
|  | GCTCACTCGCTTGAAGAAGGAA | GTTGTTCTAACCCACCCAGTTTGTATTA | 121682545 | 121682918 |
|  | GAACACATTGAGAAGATGACATGGAAAG | TGTTTATGGAAAGCCCATAAGATATCGTTT | 121568081 | 121568455 |
|  | GTCAGTATCTTATCTTGATTGGTGTGTGT | CCTTTGGAACAGTTACATATTGGACAAAAT | 121691368 | 121691720 |
|  | GGAGGATTGTCGGTGTGTGAAT | GTCAGAGACAGCGAGAGAGGAATA | 121513010 | 121513345 |
|  | ACAGTGTGTCTTCCTTTTAAATATGTGGT | GATTCACAATAGCGCCTTCTTCAATG | 121650099 | 121650433 |
| RELN | CTGTAAATCCAAAAGCATCTGGCAT | CTGTTAATGGATTTCCTCTCTCCTCTTTTT | 103363486 | 103363759 |
|  | AAAAAGGTTATTAAATGACTTGCGACTTCA | GGGCCATATTTCATGAGTATTACTTCACAA | 103280904 | 103281144 |
|  | ATCAAGATCATAACATTTCACCTTGAATTG | TTTGCTTCAAGTATTTTTGTCACAAATTTG | 103151277 | 103151510 |
|  | GCAATAACCACAGAACTGCCAT | GAATGCTTACCTGAGATCTGTGCT | 103291829 | 103292181 |
|  | ACAAAGTAAAACCTCAGGTTCCCAT | CCTCTACAGGAAAACTATAACTGTGAATGT | 103301828 | 103302201 |
|  | TGATTTGCCCATTAAACTTCAGAAGAGA | TTTTCATCATCTTGGACTAGGAAAAGAGTA | 103162379 | 103162753 |
|  | TGGCTATGTCATTATTTATCGGTCTATCCA | TAAGCTCAAGTTCCAGAAAGTTAGTTAGC | 103244677 | 103245045 |
|  | AAATTGACAGCAAATTAAGAAACACACAGT | ACAATTTGAGGTTGCTGCAGTCT | 103206952 | 103207303 |
|  | TGCCAAAATCTCTTACATCCTTGCA | AACATCGGTCTTTATTGTCCATATTCTTCA | 103193774 | 103194147 |
|  | CTGCTCAGTTGACAAACACTTCC | TCTCTTATGCATTGATGCCTAAAGATTCA | 103197328 | 103197702 |
|  | TGAAGAGATTCAGAAATCCATTTGTGTTGA | CTGACAGTTTCTTTCTTTGTAACGCA | 103201885 | 103202245 |
|  | ACATGGCAATACCATTAGCAATTAACAAAA | ACACAAATGTGATAAACAATTATGGTTCCT | 103175636 | 103176009 |
|  | TGAAATGCTTACTTGGTACATAGAACACAG | CCTTGGTTTAAGCTTTCATTCCATGTTATT | 103416920 | 103417247 |
|  | TGAGAACAGATTACTGAATGATTGCGT | CATTGGAGGTTCCAGTGCTTTC | 103557177 | 103557550 |
|  | CAAACTTCAGAAAATCAAGCTGGCAT | GGCTGTGGAACACTTAACGATG | 103214272 | 103214633 |
|  | GGAAGTTGTAGAGTAAATCTAATCAGCCT | GCTAATTACATCTTTCCTTGACAGCTCA | 103276345 | 103276717 |
|  | TATCGCCTAAGTGACCTTCGTCT | CAGTTTGCTTTTGCTATGAATCAAGAGA | 103113266 | 103113640 |
|  | AATCTGTAGCTTTTTCTTTGTGAGTTTCAC | AGTTATTTCTATTGCACCATAGCAAATTGG | 103138132 | 103138505 |
|  | CAGTCCTACAAGACATTTTACTTGTAGACA | TCTTGTGGAGGTCACTCAGTCT | 103270177 | 103270464 |
|  | ATTAAGCCCAGTTCCATAGCTTAACAA | AGGGACAAAGCAAATATAATCAACTGCT | 103389725 | 103390099 |
|  | CACTGGGCTTTTTATTTAGCGCT | GCTGGTTGTAAATCTTTTACTCAGAGAAGA | 103155596 | 103155967 |
|  | ACAGTTAACATTTTGGAAACACATCAACAA | CCATGTGAATTTGGAACTGAAACAACA | 103236815 | 103237189 |
|  | AAAATTTGTGTTCTTTGTGAGTTCCACTT | CCAGCATGTCATGAAATGAAAATTCCT | 103234039 | 103234413 |
|  | TGGGAGAATAACATGAAACACAGCA | CAGTCAAGCAGACATGACTTTCAAATAAAT | 103215916 | 103216279 |
|  | TGGTTTATTGAGCTTCACTTTTAGAAAGGA | TCTCTAAGAAAGAAAAAGTCAGCATTGCT | 103338227 | 103338597 |
|  | GGGACCCAAAGGACATTGTTATAGA | GCAGGACAATGGACCTAAATACAGT | 103141088 | 103141436 |
|  | CTGCTTCCTTCTGATTTGATTATGTTCTG | AAGGCCTCTTATCCTAAAACATGACTTTAA | 103183098 | 103183472 |
|  | GCCTGTCAGAACAGGGATGTAT | GGTTATTCTTAAGTGACATCTGGGTACT | 103294425 | 103294773 |
|  | TAAGCATGGGCTTTTATAGCTTGGT | TGGATAAGAAAAATGCTTGACCCTGAAATA | 103112260 | 103112619 |
|  | CCTCCAAGAATTCTCAGGCGTTT | GGTGCTCATTTCCCTGCATATTG | 103629312 | 103629642 |
|  | AAAAGACTACCTTCGAAATCATCTTTGAGA | GGTGTTGAGCATAGGTAGAAGCT | 103185561 | 103185900 |
|  | CAAGTTATGAAACATAGGAATGTCCTCTCA | GGATACTGCTCTGATATTCACTGAAAACA | 103206391 | 103206702 |
|  | CCGACAGGAGGTCAAGCTTTTT | CAGCCTGGTAACAGGTAGGAAG | 103292668 | 103293011 |
|  | CCCAAATGCAATGCTACTTTCTGTTTTA | TCCAATTTTCCTCAAGAGAATAATATGCCA | 103136123 | 103136400 |
|  | GGTCTACCTATAGGTAGAGACAGATGAAG | ACCTCGTCCAAGAAGTAAGTCTAGTT | 103251704 | 103252071 |
|  | AAAAACTAAGGATACTCTCCAATATGTGGT | CCACTCTCCACCATGTGAGAATT | 103205370 | 103205738 |
|  | ATAAAACATGAGACATAGCCGAAATACAGT | CTGTGAACTAATGACAAGTGACTTATTTGC | 103112843 | 103113101 |
|  | CACACACACACAACGATTTGGA | GACGGATGGGATTTTAATTAATGGAATTCA | 103132332 | 103132550 |
|  | CCTCAGGCTGGAGTTTCTATCC | GGGAAGCCCTTTTCTCACAAATAGT | 103130081 | 103130455 |
|  | GCCCAGCCTACTTTTCTCTTGATG | GGTGAAGGAGTTTTGTTGCATTATTCT | 103275598 | 103275963 |
|  | CCCTCTATCTGGAGACGGCATA | CGAAAAAGGCAGAACTTTCCATTTCTTAAA | 103179498 | 103179845 |
|  | CGTTCTGCCACCACCTAGTTT | AAAATAAAGATGATTTCCCTGTGTTTTTCA | 103123968 | 103124328 |
|  | CGCTCATTCAGTTTTGGAGACG | GGGCTTTAAGAAGGTGTGGAG | 103629847 | 103630142 |
|  | AATCCCATGAGAAGTCCTAAGTATTTGC | GATGCCATGATAGGAAGATCTTTGTTTTG | 103473921 | 103474183 |
|  | TCAGAGGAGTAGACAGTGCTGT | GAGAAAACAATCATTTAGGAAACAGCATCT | 103292120 | 103292387 |
|  | GGAAATGTCATTTGTTGGGCAGAA | ACATGTTTATGGAAGACAAAACTTCAGGT | 103155289 | 103155658 |
|  | CTTTTCCCAGGGCCATTGAAGTA | CGTTTTGAATGATTCCAACAAAGCATCT | 103214578 | 103214799 |
|  | GCTTATCTCTCATTGTCTTTTCAGCTATGA | GGAAATTATGCACTCCATCCATGGA | 103301524 | 103301888 |
|  | AAATTGTCATTAGGCAGAAATTACATGTGG | GTCCAACCTGTAAAATAAGCACCAAA | 103185288 | 103185627 |
|  | AAAAGGCAGAAGGTTACCTGTTACA | CCATTCTAGAATTTGGCAAGGTTTAAACAA | 103252010 | 103252383 |
|  | ATCTTCTTCACTGTTACACAGGAAAACA | ACTGAGTTGCTTTTGTCCTTTGC | 103194001 | 103194375 |
|  | TAAGCAGAAAAATGGCTCACAGGAA | TCTGAAGTATACTGGTGGTTTATGAGGA | 103136919 | 103137292 |
|  | TGTTCCTTGCTTTGGGACCAAT | ATCTTTGAAAAGCATTTGAAATCAGCTCT | 103138459 | 103138827 |
|  | AATTAACAACCAGAGTCCTTTTTACCCT | AGGAGATTTCAGCATCATTAGAAGAATGG | 103341237 | 103341600 |
|  | CCCAAATTTCTGGTTTGTGAGAATAGC | CGAGCTTCTTTATTCTCCTCTGTAATTTCT | 103557491 | 103557804 |
|  | TAAACATCATGCTATTGTGCTTAACTTGC | TGACATTAGATTTCTCTGAGGTCTTTTTCA | 103251014 | 103251361 |
|  | TACATCCAAAAATAGGCCATAGAATCACC | ATATCGTTTCTTACCTGAGCAGTAAACC | 103368382 | 103368732 |
|  | GCATCCTCTAGTGAGGCATAAGT | CCATAGAAAATCTATAATCTGCAATCGAGA | 103159658 | 103160032 |
|  | CAGAATGCAAAGGAGAAATGGAAGAAA | GGTAAACTTTAGTGCTTTTCCAAGCA | 103290574 | 103290946 |
|  | GATCTAAGTATGCTTTCGCCATTTAACAAT | TTTGAGAGTCCATCATAGAGGAGCTAAT | 103322460 | 103322834 |
|  | TATGTTAGCCCTTCCATACAACAAAACT | GGCAAAATTTGCTTCTTGATCATGATATGT | 103198296 | 103198625 |
|  | CAAAAGAGTGTAAGCCAAAGTGCT | CTAAGCAAATCTGAATTCCATGCATAACA | 103123182 | 103123556 |
|  | CAGTATGGCTGAACATTTCCAAGG | AGACAGGAAACTGGACATCTCAAAAA | 103270408 | 103270777 |
|  | CTAATGGTGTACCTTCTGGATGCT | AGTGTATATCAAGTGATCAGCAGCTTTTT | 103143406 | 103143778 |
|  | TACATTCAGTGTCAAGCACTAAATCCTT | ACAAAACTTTTCTCTTGTTGTTACCCTTG | 103234667 | 103235040 |
|  | AGCCACCGATTCTACAAAGCAG | TTATGTATTTTCTGCTTCTCATATGCCCTT | 103212485 | 103212857 |
|  | CCCGGTCACACCTAAGAAAGAG | CCTGGAGGAAGGCACTTTAAAGAG | 103202146 | 103202510 |
|  | GTATAACTTAAGGAAGCATGGGAGTGAT | TAGGAATGTACATGCTTTTGTGTTACACT | 103163728 | 103164089 |
|  | AAGTTCACAATCCTATCTAACAGACAATGG | TTCTTAATCTTGAATGACTCTGGTTGGT | 103126555 | 103126926 |
|  | GATGACTTCAGGAATAAACTGTCAAAGG | GAATCATTTCAAGGCTTGTTGCTCT | 103191451 | 103191804 |
|  | GCTTATGATGAGACAAGCGTTAGCA | GCTATTTGTGTAACTGAAAGGTTGCA | 103111955 | 103112321 |
|  | GGAGATAGGGTCTTCATCCACAATTT | ACAGTATTTAACATTCTTGGTCACAAAGC | 103112553 | 103112912 |
|  | TGTCCCGGAACGTAGTAGGT | CGCCTTCTTCTCGCCTTCT | 103629589 | 103629911 |
|  | AATCAGATATTTTCCCTTTATCTGGTGACA | TGTGTGTTTCTTAATTTGCTGTCAATTTCT | 103206633 | 103206979 |
|  | GCAAACTCATTTTTAGTTTTGCACACTT | TGGACTAGCCTAGAAAGGATTTTGAGT | 103292951 | 103293258 |
|  | CATGTAAAGCTCTGCCTCACACT | TTTAATTTAACCCGATCACAAAGCATTACC | 103131011 | 103131370 |
|  | GGCACCCAAAGTTCTGACATGT | CCAAATAAGAAAATGGCACACATATATGCT | 103205683 | 103206053 |
|  | TCCTTACTGGAAAAATACTGCAAGTGT | TCCTGCTCAGTCACAGACTCTT | 103179178 | 103179552 |
|  | TGTATGTATTCTACCCTGCCTTCACT | TCCGAAACAGCCAACAGTTTAAGA | 103276653 | 103276964 |
|  | ACGGACACATCAACATGAAGACA | GACTTACATGCTCTTTTTCCTTTCCTTC | 103113038 | 103113412 |
|  | ATGCTCCAGGAGTTTCCAAGTTATC | AGTATACAAGTGTGCATAAACATCCAGTT | 103275901 | 103276272 |
|  | GTTTCCTCAAGGAGGTTCTGTGT | TTTTTCAAGGGTTCCAGAAACTCCT | 103393450 | 103393822 |
|  | GGGAACAATAGAACCACTTCATATTTGTAC | GTAAGACATGGGAAATGGTTGAGGT | 103243652 | 103244025 |
|  | CCACATTCAGCTCAAGTGACGA | TTGTTAAGCATGTGGATTTCTTTTGGAAG | 103180590 | 103180963 |
|  | GGGTTATAGCATTTGGGACTGTGT | TGTAGCATCTAATTTGTTGAGTGATGACTT | 103229978 | 103230347 |
|  | CTTTTGCACCAGCCTAATAGTTACATTTC | AGGATACATGATGCAGTTTAAAGTAAGGAA | 103135926 | 103136191 |
| RET | TGTCTACAGCACTCCTCTGGTTA | TGCCTCACGAACACATCATGAA | 43617276 | 43617650 |
|  | CCTGGCTCTGACAACACACAT | CCCTCTTACTATAGTCATGTACGGT | 43601692 | 43602028 |
|  | GGGAAGAGGTGTGCTACACATG | CCATGTCCCTTGGCCTTTCAAT | 43604407 | 43604732 |
|  | GCTCCTCCTCATCGTGGAGTA | GCCATATGCACGCACCTTCA | 43614983 | 43615208 |
|  | GAGGGTAAGAACTCCAGGTCTAAAC | TCAGAAATCTGTTGTTTCCAACTAGAATGT | 43625478 | 43625852 |
|  | GATCGGGAAAGTCTGTGTGGAA | CCTTGCAGCTGTACTGCTTTTC | 43606654 | 43607007 |
|  | CAGAGATGTCAGCGATGCAGAAATA | ACTGGGAACTCTGAGGGCTAAA | 43620179 | 43620526 |
|  | GGCCTCTAAGCCAAGAACTGAATG | GCTGGAAAGGAGGTGTTGAAGAA | 43597552 | 43597919 |
|  | CCCACCATGCCACACTCTAG | ACCTGGCTCCTCTTCACGTA | 43615287 | 43615653 |
|  | GCCTCCGTAAGCAGGGTTTAATC | AACAAAGGACTCTGCCTCCAAAA | 43608406 | 43608780 |
|  | CAGTTCTTTTCTAGCCCGTGTGTAA | CTCCTGGATGCAGATCCAGTTG | 43595732 | 43596103 |
|  | TGTGAGTGGAGGCAAGGAGAT | CCTCTGGCTGATGCAGACAA | 43607645 | 43608015 |
|  | TGCACTATCCTTCCTCTCTGTGAT | GGTGGCTCCTTTCTCAACTGAA | 43622218 | 43622571 |
|  | CCTTCAGGACGGTTGTCACTTATG | TGTCTGAGCCTACAACGGAGAA | 43622797 | 43623169 |
|  | CAAGGACTGCTACACCTCTGAT | AAGAGCAGGCAGTGTTTTGGTA | 43624766 | 43625090 |
|  | CTCTTCAGACTTAAAGCACTGATAGGA | GCAGCAATTCACACTTACTGTGT | 43625086 | 43625460 |
|  | CAGGCCTCTCTGTCTGAACTTG | CTGAGCCAGGCTTCCAGAAAGA | 43613706 | 43614069 |
|  | CTGTGACCACACCTGTCATGTAG | GCAGGTACCTTTCAGCATCTTCA | 43611813 | 43612187 |
|  | AGCCATGAGGCAGAGCATAC | GGACCCTCACCAGGATCTTG | 43609843 | 43610194 |
|  | TCGTAAGCACAGTCATCGCTG | CCGAGTCGTCCTCGTCGTA | 43600205 | 43600579 |
|  | CACCCTCAGCGGCAAAATTAAT | ACCCACACATGTGAACACAGAAT | 43623677 | 43624044 |
|  | GTGTGGGTCCAACACTTACTACC | TGGCATGGAATCTCTCCATTGTG | 43624037 | 43624407 |
|  | CATGCCATCTTTACTATGTGGATGGT | AGTCCTTGGTCATTGTCATTGCT | 43624401 | 43624773 |
|  | CATCCCTCACTCACTTCCCTACT | AGTGTCAGCGGCTGTGATAAG | 43595876 | 43596250 |
|  | GCTGACATCTGTGAGCATCTGT | GGAGGGAATGCACACAGATGTC | 43618982 | 43619324 |
|  | TTCAGAAAGGCACTGTGACCAA | GGACCTCAGATGTGCTGTTGAG | 43608821 | 43609195 |
|  | TGCTATTTTTCCTCACAGCTCGTT | AGAGGCTGAGCGGAGTTCTAAT | 43615511 | 43615838 |
|  | GCTGCTAGGCAAAGGTGAGT | GAAACGGCTGTCCCGTTT | 43572766 | 43573128 |
|  | TGCATCCACTGCTACCACAAG | GATGGGAACGGCACCTCAT | 43610014 | 43610387 |
|  | GTCTTCGATGCAGACGTGGTA | CTGGCAGAGACACAGGAAGTGCT | 43601848 | 43602183 |
|  | GAAAGCTGAGGCTTCAAGGTCT | GAGATCTGCCAGGCAAATGAGAT | 43614792 | 43615162 |
|  | TCACCGTCTACCTCAAGGTCTT | TGGCATGAAGGTGAAGAATAAACCA | 43597808 | 43598182 |
|  | CCCTGCTTTGTGACCATGATGT | GCGTTTCCAGGGCTTACCT | 43607343 | 43607689 |
|  | GCAGTATCTGGCCGAGATGAA | GTGGAGTCAGGGTGTGACATG | 43615172 | 43615379 |
|  | GGGTGGGAATCAAGTCATAGTACTT | GCTCCAGCTTTAGCACTGACTT | 43622483 | 43622842 |
|  | GCGGGAGAAGTACGAGCTG | AGGACAAATGAAAGCGCGAATG | 43600473 | 43600767 |
|  | CCCTTCCCTCATTTCCAACATAGG | CTAGAGCCTTCCAGGGAGAGCAA | 43612009 | 43612377 |
|  | GTAACATGTGGCCTGAGTGGAA | GGCCTTGGTGTCATTCACAAAC | 43606436 | 43606807 |
|  | CCATACCTCCTCTCCCATAAGC | CCTACTATCTCCTACCTGTTCCCAT | 43608115 | 43608458 |
|  | CCCTGAGGATGGCTTGTTGTAT | ACTTTGGTTTTGTTCAGACCAGAAAC | 43621941 | 43622280 |
|  | TGGAAACCTGGAACACAAAACCATTAATA | CGGTAGACTTTCCATTCTCAGCA | 43623409 | 43623783 |
|  | CCCTTTCTTTGTGAACGTCACATTG | TTTTTGTGTGTGTGCTTCATGTTTAAATT | 43623785 | 43624150 |
|  | GCCCTGATGACCTGTCCTTATTC | GTACCGAACACGTGAGGATGAT | 43624171 | 43624499 |
|  | CCCTGATGAGAACAGTATGAAGAAAGG | GTAGCAGCATTAAATTACAAACACAGGA | 43624514 | 43624887 |
|  | CTTAGATTCTGACCATGACTCATAAGCTTC | CGTGTCCAATAAGCATTTTTCAAACTCT | 43624903 | 43625277 |
|  | GGACACGTAACCTGGCTCTAATTT | AACTTCTGCATTTACGTGTATGAAAATTCC | 43625271 | 43625638 |
| RUNX1 | CCAATAGACTTGTAATCATATGCCTCAGT | GGAGAAGAAAGAGAGATGTAGGGCTA | 36420976 | 36421328 |
|  | CCATGTTTTACTCAATAATGTTCTGCCAA | ACATATTTGAACAAGGGCCACTCAT | 36171493 | 36171854 |
|  | AGGGATTCCATCACAGAAATCACTAG | CCTCTGCAACCTAAAAAGAAATCATTGAAT | 36252763 | 36253135 |
|  | GGCTTGTCGCGAACAGGA | CCTCCTACCACCTGTACTACGG | 36164351 | 36164673 |
|  | GCCAGTACCTTGAAAGCGATG | CCGTCTGGTAGGAGCTGTTTG | 36259132 | 36259459 |
|  | TCCAGAATAACACAAATAACCAACAGTTCT | TTGATGAGGCTGATCATTTGGCT | 36160624 | 36160991 |
|  | CATCAGGACGGAATGTCCCAAA | CCTTTCTCCTGTTTATGGGTTTCTTCC | 36161189 | 36161459 |
|  | TCATAAATAGGGACATGAGTAAGCAGTAGA | GGCTTGTCACTATTGTAGGCTCA | 36161707 | 36162056 |
|  | GGGCTAATGGTGCTTTTCAGAAATT | GCATTCAAATTAGGCAAATCTTTGGCT | 36162264 | 36162615 |
|  | ATAGTGCCATAAAATTTACAGCGACATTG | TGTGTAACAGAACACAGCCAAAGTAAA | 36162820 | 36163191 |
|  | GCAAATACGCATTTTGCAATTGATAAGG | CTGGAAAGCAAACAGGAAGATTCC | 36163904 | 36164144 |
|  | GCTCCTAGCAACCGATTGCTTA | GTTGGGACTTTAGACAAAACTCACCT | 36259643 | 36259879 |
|  | GAGCAGTGCTGAGCTAGAAGT | ACGGTGGAGGAGTATCGTCT | 36260130 | 36260482 |
|  | TACTTGTCAAACTGTTTATTTGCCATTCAC | GGTCACAAAACATCCTGAAAATTCGTG | 36160109 | 36160483 |
|  | TTCGCAGCCAGGAAAGAAGTTGA | CGAGCTGAGGGAATGGAATTCC | 36260729 | 36261058 |
|  | GTGAGCTACTCACTTGTTTGATTAACATG | TTTCTTTTGGACTTTTGGGTAGTTGTTTTT | 36163428 | 36163783 |
|  | AGTGGGCTCCATCTGGTACTTA | GGAAATCCACAATACTTTTTCTGATCTCTT | 36206684 | 36206980 |
|  | GCTGTGCATCAAGTGATTTCTGC | CGGCTCTATAAAGAATTGTCCTTATTTTCG | 36193707 | 36194056 |
|  | CCATACCCAGAGCTCCTCGTAT | CTTTAACCCTCAGCCTCAGAGT | 36206434 | 36206738 |
|  | TATATTGGAATCAGCAGAAACAGCCTT | CACGTGCATAAGGAACAGTTTATTTCTTAT | 36265078 | 36265450 |
|  | CGGGCTGGGTTATAACTTTTCC | TACGCACTGGCGCTGCAA | 36258813 | 36259182 |
|  | GACAATGCAACTTTTTGGCTTTACG | GTTGTCAAAACTGGTAACTTGTGCT | 36231622 | 36231996 |
|  | TAACTTTGTTGATGCAACTCTTCTGGA | TAGGATTGCAGATTTCTGCCTTGAA | 36160931 | 36161246 |
|  | CAGATCCAATATGGTTGGCAATGTC | GTGTCTGGTCCAGGAGAAGTATTG | 36161397 | 36161771 |
|  | GCTGGTAAGAGTCTGTGAAACTCTAAAT | GCAGCTAAATTACCATAAAATGTCAGCAAA | 36161995 | 36162329 |
|  | ACAAGTTGTTTTAAATAAGTCCAGCTGTTT | CACTACTGTCCGTGGCTACAAT | 36162548 | 36162881 |
|  | TGATTTGGTTCCTATGTAAATGTGGCT | GCTTGGTTATCCAGAAGTATTGTTTACATT | 36163127 | 36163497 |
|  | GCATTGCTAAATCAGAAGCATTCACA | TGTGGAGGCCCTACTGAGG | 36164084 | 36164448 |
|  | GCATCACAACAAGCCGATTGAG | AGATTCGTGGAAGATAGTCTCCACT | 36259406 | 36259700 |
|  | GGAAGTTCCAGTAGTTAATGCCTGT | TTTTGACAGAATTCATTGAGAGAACAGAGA | 36259818 | 36260191 |
|  | TAGCCCTACATCTCTCTTTCTTCTCC | TCACATGATTGGCAAGAACCAATTG | 36421303 | 36421677 |
|  | CGCCGTAGTACAGGTGGTA | CCGCAACCTCCTACTCACTTC | 36164650 | 36164955 |
|  | AAGCATTAAAACATGCTTCAGTGCT | TACCACAGTTTCTTCTGTAAATCCAGTG | 36159868 | 36160177 |
|  | CCATACGTTTGTACCAGGGAGAA | GTAAACAGCTAATAGCATGGTTCCAATTTT | 36160423 | 36160736 |
|  | TCTACATCAGCTGGGTGACTCA | TTTCTTCCTTTTGAATTAACTGGCTTCTTG | 36260420 | 36260792 |
|  | CCAAAGCTGTAGCTGTTTCTCAAAAA | GCCCTGTTTGGCATCTAATTCTTATTTTTA | 36163711 | 36163972 |
|  | CTCAAATGAAAATACCACTAAAACCACCA | TAACACATGGGACAATTGTGAGGATTAAA | 36193399 | 36193769 |
| S100B | CTTCAGGGCAGCTGAGAAGATA | TCTGAGCTTGAAAAGAGTGCCAA | 48022102 | 48022458 |
|  | CATTTCTTCCTGAGCGTCCTCTT | AATAAAGAAATCGCACAAAAAGCTGACT | 48024885 | 48025255 |
|  | CGCTGTTGCTGCGCTTTT | GGATCCTTGCCTCCAACGTG | 48018254 | 48018625 |
|  | ACCAGCTGTTATCTGCATGGATG | GAAGCTTGATTTGCTTTGTGATTGAAAAAT | 48018815 | 48019160 |
|  | CCCAGTGGGAAGCAAAATCTTTACT | GTGAGACAAGGAAGAGGTGAGAA | 48024577 | 48024941 |
|  | GCCGTTAAAACAGCCTTTGGAA | ATGGTCCAAAATTCCATTTAAAAACAAGGT | 48019098 | 48019471 |
|  | TCCCAAGCTGCGCTCTTTTTAT | AGCATTATTTGAAAACACTGCTGTTCTTTA | 48018517 | 48018878 |
| SCN9A | TCATCCTCACTCAGAGGTTCAGT | AGTATGATTTGCCTGTTCCAAATTACAAC | 167055816 | 167056090 |
|  | CCTTCTGCTCTCATTGTCTCCAA | TTCTATCAGTAGGTGCTTCAGCAAC | 167141182 | 167141384 |
|  | CTAGACGTAGGATTCGGCCAATC | AGTTGACAGATAAAACTGTTTAGAGTCATCATT | 167056279 | 167056462 |
|  | TTTTATACAGAAGGAAGCCAACAGAAACT | CGCATTGCTGAAAGAAAATCAAAGGA | 167167954 | 167168191 |
|  | GAGTTTATACACACAGTGACGACACA | TGCATCAAATGAGTCAAATCAATGCTAATT | 167160644 | 167161018 |
|  | CCTTGCTGTCTTTCCCTTTGTCTT | GGAAGAAAGGTTCATGTCTGCAAAT | 167055196 | 167055560 |
|  | AAAAACCTCCTAATACAGGCTCTTAACA | CCCTATAGAAGAAACCTTGAGTTTGTAACT | 167149657 | 167149980 |
|  | GGGTTTAACCTAAATCATAAGTTAGCCAGA | GCAGCCCAAATATGAATATAGCCTCTAC | 167082841 | 167083206 |
|  | ACTCATGAAATGGGACACTTACAACT | GCATTTCTTTCATGTTGCCTATTTAACATC | 167085179 | 167085553 |
|  | GAGGTAAAGAACAAAAGAAAAGCAAGCATA | TGTTCAAAGAGACAAAATAGTCTACAAGCT | 167162873 | 167163247 |
|  | CACAGATATATTTCCACTGGCTGTGTT | GGAAAAAGACCATTAAGATTATCCTGGAGT | 167094430 | 167094752 |
|  | GGGATTAATTCTAGGTAAAACAACTTGCCA | GGTTGAGGGAGTATCACAGAAAGC | 167098905 | 167099267 |
|  | AACACTAATTAAGGAATGCTAACCAAGGT | ACAGGATGGTGTAGATAGAAAGATCAAGTA | 167108229 | 167108602 |
|  | GTCATATGTTGACTTTGACCCTCCTT | TTCTTTACCTTGGAGGTCAAGACATC | 167060608 | 167060982 |
|  | GGCCAACACTAAGGTGAGGTTAC | GCAAGTGGAACAAAATAACAGAGATATTGA | 167133745 | 167134036 |
|  | AATCATTATCAACTCCCAAGATGGTAACC | GTAGATCTTGCAATTACCATTTGCATAGTT | 167136616 | 167136974 |
|  | GTATAGCTTCAAGAGAGCATATGCCA | CCTTATTGTTTACATGATGGTCATGGTCAT | 167133194 | 167133535 |
|  | ACTGACTTTACCCACCTATGGTTATTCT | CAACTAAATTAGGATGGCCAGGGAATT | 167051637 | 167052011 |
|  | CTCTTCTTTCCAGGTGGCGTAA | GCTGTCGGCTTTCCAATTCC | 167232058 | 167232417 |
|  | TGATACACATTTAGCAATTTGGGTGGTA | GGTTTCCTAGGATTTGGAAATGACTCATTT | 167142817 | 167143189 |
|  | AAACCATTTTATGGTCTCTGAATTCTTCCT | CCAGCAATCTAGGCTCTACTTTTAATTACT | 167138072 | 167138435 |
|  | CTAGAATTTGAATCAGGCTCTATCTCCA | AATGGACATGTTGAATACAGCAAAACTTAC | 167083995 | 167084369 |
|  | GGAATTATGGAACTATCCTGTGTTCCT | GCATAATGAATACCCTAGAGAGTGAAGAAG | 167159371 | 167159638 |
|  | TATTGTTGGGTGTAATAAGTTCAGCGAT | AACTGGTTTGTTAACAACACAATTTCTTCT | 167052631 | 167052963 |
|  | TATATGAAGCATTCTTACCACTTTGCTGT | CAAGCAGAAGATCTGAATACTAAGAAGGAA | 167128891 | 167129184 |
|  | AAAACCTATTTCCAACAGGCTTGGTA | TACCATCTTAGGTTCATTCATCTTAGGCTA | 167053208 | 167053342 |
|  | TGCTTCAAAACCTCCCACAAAAATC | GGAAGATGTCACCTTCTCCTTAAAATTCTA | 167053624 | 167053929 |
|  | CTGTGCCCAAGTTATTATCTGCTCA | ACCTTTAGAGTATTGTGTTTCATAGCCTTT | 167054134 | 167054498 |
|  | GTCAAGCTCCCTAATAATGCATTTTAACT | CCTAAGAAAGGTGGGCAGCATTAG | 167054679 | 167055044 |
|  | AAATTAGGTTCTCTAATTTTCATCCGAAAGA | TGGAAGCAAACAGGTTTTCTAAAAACTTTT | 167052230 | 167052589 |
|  | TGACCCAAAGATGATAAAGACGACAAAA | CCTGGTCAAACCATGTGAAAAAGAAAATAA | 167083140 | 167083289 |
|  | TTGTGTTCAAGAATTCAGCATATACTTCCT | TGTCTCAACATGGTAACCATGATGG | 167060374 | 167060663 |
|  | CCTTTAAATACAGACATTCCATGCTGGAA | AACAGCCATCCTCCAATATATGTCTTC | 167162182 | 167162548 |
|  | TGGTTGTGATGGGTTCATAGGAC | TCATACTGGAGAATTTTAGTGTTGCCA | 167055502 | 167055876 |
|  | GCTTACTGTTAAGAATAGGTGCTAGCAATC | TGTTTCTAGCTGATTTGATTGAAACGTATT | 167056015 | 167056371 |
|  | TGAAAAGGAGAGGTGGATTCAAACAG | TGCTGAGGAACTTAGCAGTGATTC | 167128580 | 167128954 |
|  | TAAAACCAGAGTCTTTCAAGGTGCA | GATGGCAGTGTTTCTTCAATATAACACTG | 167163357 | 167163699 |
|  | AAAGTCTTGCTTTCAAGAGTATGCATT | GGGACCAGGCCTGAATTTGTAG | 167150913 | 167151285 |
|  | CCTGGTCGAGGAATTGGCTTTT | GGAGACCCATGTTTCTACACATATGTAATT | 167060871 | 167061157 |
|  | AAGGTCTTTCAAAAATGATTGAACCCAAA | GAGCACATGAAAAGAGGTTGTCTAC | 167142646 | 167142880 |
|  | TCCATTTTTGATTAAAAGGACCAGAGTACA | GGCCTGTTTCACAGATGGTAAGA | 167107927 | 167108291 |
|  | AATGCATGAACATCTGGTTACATACCA | ATGCTGATTAAGATCATTGGTAACTCAGT | 167133468 | 167133807 |
|  | TGCTCATTTCAGCAAGTGTATGGTTA | GCTGGTTTGTATTGTGGCCTATATTC | 167129118 | 167129492 |
|  | CATATACCGCAGAGCCTCTGTT | GATTTGCTCATGCCTGTCAAATTGAAATA | 167144872 | 167145235 |
|  | AGAAACAACTCATAATTTCTACATACCCATTGT | TCCTGTTGAGTTGCTTTTAGTGAGTTT | 167089758 | 167090069 |
|  | AAAGTCACAAGTAGACATTCCTGAAACA | GAATTCAATCTACCGTTATTTCAAACTCAC | 167052807 | 167053117 |
|  | TGGTACTGTGTTGATTGAGGCAAAATAT | CCTGATATGCAGTTAGTTGTTTGACCA | 167053861 | 167054196 |
|  | TTCTGTGGTTATTCTATGAACAAAAAGGGA | CAATGAAATGCTTGATAGAAGTAATGGACA | 167054428 | 167054753 |
|  | ACGATTCTTAAAGAATCATCAGTGCAAAA | CCAGACAAAGAGAAATATGAACAAGACAG | 167054981 | 167055259 |
|  | TCTCTCAGGGCTGCTTCTTTTTC | GCATGGTGCAGAGGCTACA | 167232295 | 167232666 |
|  | GGGCTTCTTCATCATCATCTTTCTTTT | CCTTTCTTGGCAGGCAAATAGTTAAG | 167168128 | 167168367 |
|  | GCATTTCCAGAATGAAGATGTAAGTGAAGA | GAATCATGAAGCTTAAGTTGTGCATGAT | 167094681 | 167094830 |
|  | GGGTGGTGTTCCATAGCCATAAAT | AGTGAAACAGACAAATGGCTGACT | 167136910 | 167137155 |
|  | CAACCTAATAACAAATGCAAGGACATTCTT | GTTTCGATTCAGAGGCTTTATGTCATTAC | 167159568 | 167159866 |
|  | TGCCTTTTGATTCAAATTCCCAAAAGT | TCTGGAACACAGTAAATGCTAAGCA | 167134549 | 167134922 |
|  | GCCAAGACTGGCACTGTTTTAAAA | CTTTGATAATGTCGGACTTGGTTACCTA | 167085064 | 167085243 |
|  | AAAGTTTTTAGAAAACCTGTTTGCTTCCA | CCCTTCAGAAGGCACTGTCATATTAATATT | 167052561 | 167052699 |
|  | GAAAACACTGCATGGTGTCTTTCATATTAA | AAACAACTTTCACTAATTTGCTTTCCATAT | 167053265 | 167053615 |
|  | CCCTTCAGCAGAGAGACTGACT | TGAGACTGAATTTGCCGATGATGA | 167140890 | 167141239 |
|  | GGTGGCCTTCATACATAGGTCCT | CCCAAACAGGTTTGAACACAAATC | 167051951 | 167052282 |
| SLC6A4 | ATCAGAACTGGAGGAGGAGGTT | CCAACTCGCTCTTAGATGTTATTAAAGTGT | 28525420 | 28525671 |
|  | CACACTGAAGCCACATTTCAGTT | CAAGAATAAAAGACACTGACATCCATTCAC | 28542994 | 28543368 |
|  | GCCCAGCCTTCTTTGGAAAATTT | CACACGGACAGATGCTCCTTAG | 28539634 | 28539958 |
|  | GTGTGAGATGGAAGGAGACGAA | TTGACCAGTCACTTAGCAACCTTAC | 28537445 | 28537769 |
|  | GATGCCTAAGGCCTGACTGATT | GGCTCCTGACACTCACATCATC | 28545705 | 28546059 |
|  | GTGCCCAGCCATTACAATTCAT | ACTCTGACTCTCCCAGAGAATCAG | 28534665 | 28534938 |
|  | CTGTGTCCAGTCTATCTGCACA | CGTTTGACGACTCAGACCTCAAG | 28549748 | 28550102 |
|  | GTCCAACACACAATCAAAAGTATTCTGAAA | GCAATTTTTAAACACTGACAGTCCAAACA | 28521499 | 28521799 |
|  | CTTTTGCACCAACTAATATTTACCACTGAA | TATAAAAAGAAGGTCCAGACAGCAGAGGAA | 28523619 | 28523993 |
|  | CCATTTCCCAAGCAGCCTTACT | GATCTGGCCTATTTCATTAGTCACCAT | 28524235 | 28524596 |
|  | GGCCCAAAATATTGGACTAGAGATAAGA | GAATCAAGTCTGTGAAAGTCTCCTGTATC | 28524842 | 28525172 |
|  | CCCTCACATGGTCTGATCTCTAGA | AGAGCAGGAAAGTCAGGATTCCT | 28562755 | 28563044 |
|  | CCCTCCATTCTGGTAACATATGTAGG | GCGTGTGAAGATGGAGAAGATTGT | 28548635 | 28548937 |
|  | CGCATTGGAGCAAACTGACTTG | CTGCAAGAACTCCTGGAACACT | 28544899 | 28545237 |
|  | TGCCTGGTGGAGAGGGTATTA | GCCAAACGTTCCACTGCATTC | 28522570 | 28522906 |
|  | CATTCAAACTTATTCTGACGAGGTCCTT | AAAAACCCTCTCAATTGGTTTAATACCA | 28521908 | 28522276 |
|  | CCCAGCCTCTTGGGTATTTCT | TAGCGTTTTGCCAAGTCTGGTATT | 28523129 | 28523465 |
|  | AATGTACCAGAGGGTGGTAAATGC | AACTGTTGTTGTGGCTGAGTTTTC | 28538157 | 28538530 |
|  | GTGGAAGGAAACAGTGCTGTCT | CCTCAGTGACCACTTGGTCCTA | 28544083 | 28544452 |
|  | CCTGGTGAATCTCATTTTGAATAGAGCA | TGAAAGTATCTTAGCTTTAGCATTTGGTGT | 28530088 | 28530462 |
|  | GGCAGTGGTATCAAGGCCTAAG | TGAGCCTTAAAAGCTGTTTTTCTGC | 28542505 | 28542860 |
|  | ATGCAGTGATGTTTAGGGAGATGTTT | ACAGTTATTTTGTGAATGGCCTTTAAACTA | 28521205 | 28521579 |
|  | GCCAAGGGAGGAAAACCTAGAATT | CGATTGTGAGCCCTCCAGATAATTTTTAA | 28523929 | 28524296 |
|  | ACAAGATCCACCTTCAACGTACTTC | CAAGCTTGTGAGTCTGTGTATATTGTTG | 28524534 | 28524908 |
|  | AAGCAAACAGATATCAGATACCAATGATCA | GTAACACACTCACCGAGAGGAAAAA | 28525103 | 28525477 |
|  | GCAAAAATATGGGACATCCTTCCTCA | CAGCCAACTCTAATTTGGAGACAGT | 28521734 | 28521971 |
|  | AAAGTTTGAAAACTTTCAAAGCTCCTGT | GCATGCTGAAAGAAGGAAGTGATCTAA | 28523403 | 28523686 |
|  | GGGAAGAAGGTCTGGAAAGAAACG | GACGCAAAAATTCTTCAAGAGCTCTTT | 28562574 | 28562816 |
|  | GTTCCTAGTCTTACGCCAGTGAA | CTCTCAGTGATTGGCTATGCTGT | 28548336 | 28548709 |
|  | GTGGGAACAACCTTCTGTAGAACT | GGATGTCTCACATTTAACAGATGCATTTG | 28548879 | 28549231 |
|  | TGATGTTGTCCTCGGAGAAGTAATTG | GGAGTCCTTGGAATGGAATGGAG | 28545179 | 28545499 |
|  | CTGGCCGAGACTCTTTTACAGA | CCAGGTGGGATTTTTCCAGAATG | 28536014 | 28536373 |
|  | ATCTTTTCTATCGTGGTATTAAACCAATTGAGA | AGAGTGAAATAATACCCTCTCCACCA | 28522236 | 28522599 |
|  | GTCTTTCCATGGAACTAAGCATAACAATTTTT | TTTTTCTTGTATCAGTAAGAAATACCCAAGA | 28522810 | 28523166 |
| SOD2 | CCTTTCCCATGGAAACTCAGTGA | ACCACGTATAAACATAAATTGTATTTCCTGTTT | 160100065 | 160100439 |
|  | GGTGCTGAAGACGAGAAAGCA | GGTAGCACCAGCACTAGCA | 160113892 | 160114220 |
|  | GCACCCGATCTCGACTGATTTA | TGGTAGATGTCACCCAGTGGTT | 160102720 | 160103059 |
|  | CCAGAAGTATCACTGCAGAGGATTAC | GCATTTCTTGATGTTGCTTAGTCACTT | 160103057 | 160103431 |
|  | AGAAATGCTACAATAGAGCAGCTTACTG | ACAAGTGAATGAGATTGTTACAAAGGGT | 160103424 | 160103796 |
|  | ATCAACAATCGATTCCTACTGTGCA | GGTCCTTCATCAGTTGTTCAGCA | 160109097 | 160109468 |
|  | AGAGAGACTATCGTGCCTGGAAA | CGGGCTGTGCTTTCTCGT | 160113547 | 160113919 |
|  | AATCACTTGCCCAATAACAAAATGTTTAGT | ACTGAAACTGATGGTTGGTTTGTTTTC | 160103339 | 160103713 |
|  | GGGACTAAACTCTGACCTCCATTCT | TGCTTGAGACCCAATCTCCATG | 160102459 | 160102833 |
|  | CTTTACTGTGCAGGTGAGCAAAC | CTGATTGGACATTTTCTTCAGAGAGCTA | 160102934 | 160103308 |
|  | ATTTCTAGTTGAATGCTTTACAGTAGAGCA | CGCATTCTGATGTTGTCTAATTTCTTGG | 160105820 | 160106193 |
|  | CCGGCTCAACATGCTGCTA | CGCCACTCAAGTACGGCA | 160114188 | 160114541 |
| TH | GGCGTACCTGCGAGAACT | CTTCCGCGTGTTCCAGTG | 2187703 | 2187928 |
|  | CGGCACCATAGGCCTTCA | CTTCCACCCACGAATTGTGAC | 2186927 | 2187140 |
|  | CAGGGCGAAACCTCTGAGT | CCCAATCACCGTCACAATAAAAGA | 2185063 | 2185196 |
|  | GAAGCTCTCAGACACGAAGTAGAC | GTTTGGGAGAAGGCTTGTCTCT | 2186485 | 2186778 |
|  | GCTTGGTAGCCTCAGCCCTA | GTGGCCTTTGAGGAGAAGGAG | 2190652 | 2190981 |
|  | GCACTGATGCTGGTGACAAGAT | GCAGGAAGCTGATTGCTGAGAT | 2188983 | 2189135 |
|  | TCCAGGTCAGGGTCGAACTT | TTGAGGGCCACCAAATGACCT | 2189334 | 2189701 |
|  | CTTGGAGCTGAACTCCCAAGAA | TGAAATGTGGTGTGAGTTGTAGCA | 2191790 | 2192132 |
|  | ACAGCTGTTGCGCTGAGAA | CCCTTCTCCGTGAAGTTCGA | 2185305 | 2185597 |
|  | CTGGGCACACCCTTCAGGAAG | AAGAGGCCTGCGTTGGTAG | 2188107 | 2188374 |
|  | GGGTCGGTTTTCTCATCTGTGAC | GTGCAGCCCTACCAAGACCAGA | 2186340 | 2186541 |
|  | GGACGCGTGGCGGATATAC | GCTACCGGGAAGACAATATCCC | 2187886 | 2188170 |
|  | CAATGGCATCACTGACTCCACT | GAGTTCGGGCTGTGTAAGCA | 2186608 | 2186975 |
|  | CCCAGCCTCTCAAGGTCATTTG | GCTGCTAGCACAAAAGTCAAGG | 2189669 | 2190027 |
|  | CGACAGGATGGGTAGCCTCTT | TGTCCATCCTCCAACCCAAAG | 2188486 | 2188845 |
|  | CCTCACTGCCTGTACTGGAAG | CACCTGGTCACCAAGTTCGA | 2189090 | 2189365 |
|  | AACTCCACCGTGAACCAGTAC | ACCTCGAGCATGACCCAGGG | 2186971 | 2187345 |
|  | CCAGCTCACCTCAAACACCTTC | CGCTCAAAAACGTGCTCTCATC | 2190871 | 2191231 |
|  | GGGTTTGCATGGACCCTGA | GCTTTGACGTCAGCTCAGCT | 2192808 | 2193094 |
|  | GCCAGGCAGGTGTAGAGAC | CTGAGTGCCATTGGCTAGGT | 2185147 | 2185480 |
| TLR4 | TGGCTCACAATCTTATCCAATCTTTCAAAT | CTTCATTTCTAAATTCTCCCAGAACCAAAC | 120474876 | 120475205 |
|  | CTGGACCTCTCTCAGTGTCAAC | GCATGCCCTGCTTATCTGAAGG | 120475907 | 120476261 |
|  | CTCTGCCTTCACTACAGAGACTTTATT | ACTGTTCCTTCTGGATTCCATGATTTAC | 120476519 | 120476886 |
|  | ACTGTCATGAAAGCAGCATTGAAATAATTT | CAGGAAACAGCAATGTATACTCCCT | 120477735 | 120478098 |
|  | CTGAATTTTGTGCTTGCACAAAAAGAG | ATGAGACTCCACAAACCAAGCTT | 120470728 | 120471102 |
|  | GCTTTCACTTCCTCTCACCCTTT | GGGCAGAAGTGAGGGAAAGTTC | 120466574 | 120466890 |
|  | CATTTTCCCTGGTGAGTGTGACTAT | GCCCAAGAAGTTTGAACTCATGGT | 120475341 | 120475666 |
|  | TGGGCATTTCAACCAACTCAGT | GTTGTGAGCATGTGTTAATCAGGTT | 120477135 | 120477492 |
|  | CACCTTATACCAGGTAGAATGGCTACTATA | GTACGTATATGCACATTTTCTTTGTCCATT | 120478299 | 120478640 |
|  | GAATAGAACAGTGGTTCCTAGGGAAAAG | CCTCCAAAAGCTTCCTTGTGTATATGTATA | 120478808 | 120479040 |
|  | AGACAACAAAATTCAGTTGTCAAAACTGG | CTACTGTACAAGCACAAGAGTAGAGAAC | 120479180 | 120479520 |
|  | TTGTATTCAAGGTCTGGCTGGTTT | GTTGCCATCCGAAATTATAAGAAAAGTCTT | 120475141 | 120475406 |
|  | CTTGGTGGAAGTTGAACGAATGG | CATGGATGATGTTGGCAGCAATG | 120476206 | 120476579 |
|  | TTCTGGAGACGACTCAGAAAAGC | AGGTAAGAGTTGAAATGACTTTCTTTGTCA | 120476825 | 120477197 |
|  | CCTTACAGAGGTTAAAGTCTAGTGGCTA | GAAATTTTCTTCCCGTTTAAAAGAGTGC | 120477429 | 120477803 |
|  | TGTATGAAGAGCTGGATGACTAGGA | CAAGTGCTCTAGATTGGTCAGATTAGAAAA | 120474574 | 120474946 |
|  | CAGCCTAAAGTATTTAGATCTGAGCTTCA | GAGAGTGAGTTAAATGCTGTTGGAGA | 120475603 | 120475965 |
|  | CATATGATAAAATGAGGCTCACTGAGGTT | TCCATCCATTCACTCAATTCATGCTTATAA | 120470411 | 120470684 |
|  | CCCAGCAAACTAATACAAATAGTGTTTCC | CCACAGCAATTGGTGTATTCAAAGC | 120466291 | 120466632 |
|  | GCCAGGAGAACTACGTGTGAAG | GGTTCCAATTTCTCTATATCCTTGATGACA | 120478041 | 120478369 |
|  | CCAAAGTTATGGAAACAACCCAAATTTCC | CCAATTAGACAACTCCCTATTTCCTCATTT | 120478571 | 120478876 |
|  | CTTAACATTTTGTTAAGAGGGTACCTCTCA | ACACTCAGTAACAAACACTTCTGACTG | 120478963 | 120479246 |
|  | ATCCATTTGAAATGGATGTCTATGGCT | GGTTTCATGGTCAGTTATGGTCTGAAA | 120479455 | 120479821 |
| TNF | GGCTGATGGTAGGCAGAACTTG | ACCTGGGAGTAGATGAGGTACAG | 31544652 | 31545025 |
|  | CAGGGAAAGAGCTGTTGAATGC | AAAAAGCTGAGACCCTTAAACTTCCTAG | 31544150 | 31544523 |
|  | CGACTCAGCGCTGAGATCAATC | GAATCCCAGGTTTCGAAGTGGT | 31545231 | 31545487 |
|  | CAGAATGCTGCAGGACTTGAGA | GGGAACAGCCTATTGTTCAGCT | 31545623 | 31545922 |
|  | CTCCAGATGAGCTCATGGGTTT | TCAGTGCTCATGGTGTCCTTTC | 31543225 | 31543529 |
|  | TGGAGCTGAGAGATAACCAGCT | CAGACTCGGCAAAGTCGAGATA | 31544962 | 31545282 |
|  | GGTAGGGTTAGTACCGGTATGGA | CATGACGTTCTGAGTATCCCACTAAG | 31544403 | 31544757 |
|  | GTCGGAACCCAAGCTTAGAACTTTAA | CCTAAGGTCCACTTGTGTCAATTTCT | 31545429 | 31545681 |
|  | AGGTTCTCTTCCTCTCACATACTGAC | TCTTTCACCCATCCCATCTCTCT | 31543457 | 31543791 |
|  | GCCTTGGCTCAGACATGTTTTC | CTTCTTCCCACCCACAAGAAGAG | 31545868 | 31546171 |
| TRPA1 | ACAGACCTCAGGGATGTACACA | GAAGCAATATCCAAAGATCTCCTGATCATA | 72933295 | 72933669 |
|  | GGGAATATGTGATGGAGCCATGT | GCTTCATGTTGAAAGAACCTATGGGT | 72934367 | 72934665 |
|  | ATCTATATTTGTGTCTGCAATTGTTTTGGG | GAAGAGCATGGGTACAGTAGACAG | 72974815 | 72975177 |
|  | CCCAGAGTACCTTAAGCGATTCC | TCCGCGTTTCTACCTCCTCT | 72987524 | 72987894 |
|  | GTTATTGTCTCATTTCCCTTACAGCCTA | CAAGGGTGAGCATTGCATTTCAT | 72962875 | 72963239 |
|  | CTTGTAGGAGCCTCTGACAGGTA | ATTTTCCTACAGAATAGTTTGAAAACCATCA | 72967798 | 72968171 |
|  | CCCTTAAAAATCACTTCCTTCCATTCTTTC | CCATAAGTTTTGCATATGCTTGTTTGG | 72983860 | 72984154 |
|  | CTACTCAATACCCACCATTTCATGAGTAA | AAAGCTTATGCTAGGTTAAGTAGTCAGAAA | 72951947 | 72952287 |
|  | CCATAATTCACAGCGACAAAATCAACT | GATCTCATCTGTGAAATCTAATACCTGGTT | 72946437 | 72946723 |
|  | CCACTGAAATTAAATAGTCTGCTAGCTCA | TTCAAACATCTTTACGGTGATTTTACAAGA | 72971624 | 72971954 |
|  | CTTATGTTCTGCATATGAAAATATGTGCA | CTAAATGAGGGTGTGTTCATCTCTAGTAAA | 72950160 | 72950504 |
|  | CATTGAACTTACGTTGAGGGCTGTA | CTCCACTTGAAATAACTCAACTTTGTTCAA | 72958736 | 72958991 |
|  | TGGGACAAATATTGTGAAGGAAACAAGT | GGAAACACTTTTTCATTGTAGAAACTTTGC | 72945900 | 72946122 |
|  | CCGTGGTATCTAGTATTTCTGAATGATCAC | ACAAGGTTTCTGAAAGAGGTTATAACCATT | 72951099 | 72951364 |
|  | GCATGCACCTATAGTCCTGTTACTC | CATTTTCAGGTTCCAAAGAATGCATG | 72975483 | 72975742 |
|  | GTGTTCTAGCAAATTCATTTTCTACCCATT | GTGCATGACTTGCTGGTTCTAAC | 72934855 | 72935106 |
|  | TGTCCATCTCTAGAAAAGTCCAGTGT | CCTGATTAGCATGTTTATTCCCTCACTAC | 72933902 | 72934266 |
|  | AGTCTTCTATAAGCAATCTCAGATCCTCTT | GTGGAAGGCAAGTACCTTATTTGGA | 72977485 | 72977859 |
|  | ACACAATAGATGGTGGCACACAA | GTTCAGTGCTAGCCATAATATAGACACTC | 72969844 | 72970172 |
|  | TTGCTAAACACCCAACAAAAGGCT | ATTTAGTTGCCAGATCTCAATAATGCTTTT | 72965857 | 72966230 |
|  | TGACTCTCAATACACTAGCTACAGCT | GGTTCCTTGAGGGCATAGCTAT | 72935903 | 72936273 |
|  | GCCACATGATCAGGAGGGTAAATG | TACACTACCCAGACTAAAATATTTTGTCACAG | 72941969 | 72942322 |
|  | GGGAAAACTGAGAATGTAAGGGAAAAA | TTCTTGAGATGCAAGAAACCAAGGA | 72959207 | 72959570 |
|  | TGTAATAATTGCATTTTGTTTGCCATGGA | GCACTCAGGAGGTGTTGAATACA | 72981127 | 72981498 |
|  | GGTTTGAGTCACATGGAAGATATAGACA | CACCTACACAGGATGTTATATATGAACCG | 72958479 | 72958791 |
|  | ACAGGCAGAGAAGACTCAAAGTC | CAGAAGTCAGGACATAGAAATCAGTTTTCA | 72938084 | 72938427 |
|  | TTTAGGCTCTAAAGAGCTTCTTCATGTC | CATAGAGCTTCTCAATCATCCTGTGT | 72951644 | 72952012 |
|  | GTCATAGGCATATAGCTGTTGAAATGC | TACATCCTCCTGAATTTACAGGTAAGACA | 72946167 | 72946503 |
|  | AATGAAAAGATAGCCTGAAAATGGCTAATTA | GCAATGTATGAACATGTCTTCTTTCTGTTTAAT | 72967603 | 72967905 |
|  | TAAGCCAGTTTTAAATTTACACCAGGTACA | GCTGCAAAGCTAGAAAAATTGTATTACGG | 72948430 | 72948793 |
|  | CTTTGAACCACAAAATCTTTGCTAGCA | GTGATCAGCCTGATAGATATTCCTTGATTT | 72973749 | 72974120 |
|  | AAACACTGATATGGATATCCAAATTACGCT | TTTTTCAGAGCTTCATTGTTTGATCACC | 72971386 | 72971681 |
|  | GGGTGGCTTTCCCATTATTCATAAAG | CTCGGTGCCCTTTCATTGTTTTT | 72975117 | 72975288 |
|  | AAGCCTCAACTCACCAAACCTT | TCTCATCCACCCTTGCGATTTG | 72975684 | 72975870 |
|  | AAGGACACATACATAGCCAAAGACATATT | CTGTGATTATTGTATGAGCCTTTGTTTGAG | 72934185 | 72934430 |
|  | CTATTCATCACAGTAGGGACAGAAAAGTC | TTCAAATTGGACTGGACTTTGATGAGATAT | 72934600 | 72934928 |
|  | TTATTTTGTCTCAGACTTCAAATCATCCCA | AGCAGTTAAGCAGTGAAATAAGACAGAT | 72964760 | 72965072 |
|  | GTCCAAGTGAAACGGCACACAT | CGGACGACACGGAGGATTTC | 72987199 | 72987568 |
|  | TTGAGGAGAGGGAAGACTGAAAAGA | GTGGCTTACTATTTCATTGTGTGCA | 72933604 | 72933966 |
|  | AGACTGAATATTGGTCTGCTTTCTTCAT | GAAATGAATTGGCACATCCAGTTCTG | 72945590 | 72945964 |
|  | CTGGCAACTACAAATCTGCTTTCTTG | GCTTTCAACTCAACTGGCATCAT | 72950796 | 72951162 |
|  | CACCACCATCACCATGTTGTAAC | TTTTTGCTAAGTTGAAAATCCACGTAAAC | 72968982 | 72969333 |
|  | AATCATTCTGCTTCTTCCTCACTCTTT | TCAGAAGGGCTAAAAGAAGAACTGAAAAT | 72935046 | 72935409 |
| TRPM8 | GCTCACTTGGCATATGAAAGAATAAACA | CTATAAAGCAGACACCATGGTCTTGT | 234888689 | 234889063 |
|  | CTTCTTGCAATTTCAGCGGCTTA | CCCACAACACTTTGAGGAATTTAAATTTTT | 234825905 | 234826241 |
|  | AGGGTACTAGGAAGAAAATCAGAAAGGTA | CTTAGAGCATGAGAAGCAATACCATTC | 234916540 | 234916914 |
|  | TGTTGTGATGGCATTTTCTATGCC | ATTGTTTAATGCTGCTAGTAGCATCTGA | 234875104 | 234875478 |
|  | GCATCCACATATGTGTGCTCTCAT | GTGCTTGTCGACAGGTTTCTTC | 234878506 | 234878875 |
|  | GAAAATCCCATCACTGTACTGTGAGA | GAGACCATGTTCATCTGAACTCAGAC | 234871785 | 234872159 |
|  | TTTTTGACCAACATTATCTCTACATGGGT | GCCGTAAACATGACTTCTTGAAGG | 234869100 | 234869451 |
|  | CTGATCCGGAACTCCACTACAG | ATTCTAGCTTCCAATCTAGGATCATCAAAA | 234862442 | 234862806 |
|  | GGTTTGGACATCAGCAAGCAAA | GTAGATGACCGAACGGAATATCCAC | 234891455 | 234891822 |
|  | TCTGTGAGCCCAGAATGGAAAC | GCACTCCTTGAAAGGCAGACTT | 234851152 | 234851524 |
|  | TGTATACTCTAAAGGCATGAACAAATGCT | GCGCAAACAGTGTTTCTGAGAA | 234839155 | 234839522 |
|  | AGCTTAATACCACTATTACCATACTAGGCA | TCCTTCCTGTTGAGATACACACCA | 234904883 | 234905247 |
|  | ATCCGTCTTTAATGAGTGGATAATGTTCA | GGGAACAGTATGTGGAGGGAAA | 234855571 | 234855944 |
|  | TGCGTGTCCTTCTTAGCACTTT | GTGGGCAAAGTCATACGTGGTA | 234893982 | 234894354 |
|  | TTATTTTCCTTTCACACTTCCACTTCCT | GTGTCGCAGGACAGACGTATATA | 234847437 | 234847664 |
|  | ATCTCTCAACTTTGGTGGTATATTTGGATC | GTCCCAGGCATTGAGACACAGA | 234925808 | 234926172 |
|  | TGGATGGTGAAGGGAATGGTATAGA | TCAAAGCTGAGAAACTAATTATGGGCA | 234927530 | 234927671 |
|  | CTTGCCCAATACTGAGAAGCAAC | TGGGAACCAAATCCCTATAGAAGTGAA | 234927883 | 234928252 |
|  | CAGATTCTAAAACATGCTGCAGCAA | ATCCAGAAATTGTTAGCAGTGATTACCT | 234926397 | 234926749 |
|  | CATTGAAGGCTATCTCCAGTTGATCATT | AATAGTTCAGGTTCATGTATCTCATATCTTATG | 234926957 | 234927188 |
|  | AAAAGGCACAGAGCGATAATTTAAAAATGA | GGAACTTCAACTTGTTCTTTCTCTATCTCT | 234923126 | 234923473 |
|  | GGGTCTTGGAGGGTAAAGAGGAA | GCAGGATTTGACGGCTTACGAA | 234834992 | 234835365 |
|  | GCCAGCAAGCTTCTGAAGAC | GCCGCTGGCAATGTCTGT | 234873302 | 234873469 |
|  | CAAGGGATCCTTAGGCAGAATGAG | GGTTTTGAGGAGTGAGTTTTTACTCTGTTA | 234891763 | 234892018 |
|  | GGATGCTGACGATGCCCTTATC | CCTCTTGGCTTTAGAAAGATGCCT | 234878821 | 234879139 |
|  | TGTTAGTACTGTCTCTGTTCTTTCTCCT | GAAGCCTCAAAATATCCGAGAACTCA | 234869389 | 234869761 |
|  | ACATTAATTCAAAGAGCTGTGTATTTTGGG | ACAATGTAAACATCATAAGTTCCAGGCTT | 234878218 | 234878571 |
|  | CCTCACTTTGCCTGTTGGTTTCT | CCCAAAAGATCTCTAAAGTATGTTCCTCAA | 234894299 | 234894631 |
|  | AAATAAGACTTGAAGGTATCCTTTGTGTGT | CATCTCTCCTGCCTCCATCCTAT | 234845886 | 234846257 |
|  | CCTGTGCAGGACGAATCTCATT | CGGGTAATATCTTTGTGGTGGTATTATTCT | 234915375 | 234915733 |
|  | TGTGATTGAACCCAAAGTATTAGCTTTCA | CACGCATCACTCAATAGTTGGTAGT | 234854394 | 234854768 |
|  | GGCCTATCCTGATACTTCTGCTTC | TCCATTCATTTGAGGATATGGCTTTCAATA | 234847607 | 234847981 |
|  | TGAATGTAAGAGAACATGATCAGCATTACC | CCTGATATTCTGGAACAGAACAATCCTTTA | 234863651 | 234864025 |
|  | CCAGCTTGGCTCAGAACCTTAG | GAGTTGGGCGTTTAAGGCATTC | 234858509 | 234858871 |
|  | CCTATTTTCACTCACTCAACCTTCCA | GATGGTGAAATGCAACAGTCGT | 234890294 | 234890668 |
|  | ATACAGAGATGTAGAAAGAACATAAATTGTCCC | AAATACAGCAATAGGATTCATGCACAAAG | 234926679 | 234927024 |
|  | AACCCTTAGTTTAAGAAGAAGTCAATATGCTT | AGGTGTTCAGTAAGATAATCTCTCCAATTCT | 234927208 | 234927582 |
|  | TTTCCTTTATGTGTTTCTCCAGAATGGT | TGAGAAATGAAAACACTTTTCCTGAAGAGA | 234926112 | 234926462 |
|  | GTATTTGAGCAGGATGTGCACAA | TGGGTTGAAAACATTTAACAGTTCCCT | 234927611 | 234927943 |
| TRPV1 | CCCTGGCTTTGTGACATTTAGC | CTGGATGGAGACCCTAACTCCA | 3495259 | 3495572 |
|  | GGCCACTCACTATGCCATCTTA | CTTTGGGATCCTCTGGGAATCAG | 3500145 | 3500468 |
|  | TTGACCGCAGGGAGAAGCTCA | CCACCACATCTCCATCCTGATG | 3474824 | 3475046 |
|  | TCTTCATCCTTGCTGGATCCTCT | GCAGCAGCAAGTTGGAGTTTTG | 3495638 | 3495801 |
|  | TCGAGCACTTGCCTCTCTTAAAA | GGGAGGCCAAGAAGTGAATGTTT | 3470220 | 3470344 |
|  | TCCTTGCCATCAGGTGTGTAC | GCCAGGCTACAGAAACAAAGCTAG | 3475437 | 3475705 |
|  | CAGGGAGTAAGGATCCCAGAGAA | TTCCTGCAGAAGAGCAAGAAGCA | 3494152 | 3494526 |
|  | TCACGTATGGTTGCTATGCTTAGG | CAGGTCTTGGCCTATATTCTCCAG | 3491293 | 3491664 |
|  | GGAGTTCTGCAGCAGGAACTT | CCATCGAGAGACGCAACATG | 3493329 | 3493665 |
|  | CCCGAGTATGTATTCAGTGCCTT | ACCCTTGCTGTTCTGCTCTTTT | 3483600 | 3483966 |
|  | GCAGGAGGATGTAGGTGAGAATTACA | CTTGGGATATTGGCTTCTATCCAGTG | 3477004 | 3477302 |
|  | TCAGGGCCAGATTGCTTGATTC | TGTACTTCAGCCACCTCAAGGA | 3480734 | 3481019 |
|  | CCCATGTCTTGTTGCTGATACTCA | GACGAGCATGTACAATGAGATTCTGAT | 3492860 | 3493227 |
|  | CCACAGGACCTCAAGGTTAAAGG | ACAAGTGGGACAGATTCGTCAAG | 3488812 | 3489171 |
|  | GGGCTAGGCATAAGACCTGAAC | GGTGCAGAGTGTGCAGTATAGA | 3498769 | 3499128 |
|  | CGGCCAAGAATGGGCTTTAGTA | CCATTTGAGTTGGCATCCCTGTA | 3512475 | 3512840 |
|  | GCTTTGCAAACTATTTGGTCTCTGT | GAACCACATCCCAAAGGTACCT | 3468509 | 3468881 |
|  | TCCTGGGCTTCATTTACTTTTGCT | ACCTGGCTGTTTTCTTTGATTTTATTCTTT | 3469137 | 3469510 |
|  | AAAAGAATAAAATCAAAGAAAACAAGGGCCT | GGGAAGCGTTCTTGGAAGCA | 3469793 | 3469959 |
|  | CTCCAGCACCGAGTTCTTCTC | CTGGTTGTTGAGGTACTGTCTGA | 3491512 | 3491733 |
|  | CTGGCCTCTGGATGGTGATAAC | GCAGTCCTTACTTTGGGAGTCAAC | 3495383 | 3495723 |
|  | CCATGAACCTTTCGGCAATGTT | TGCCTAAATCACGGTAGAAGTTCC | 3498975 | 3499339 |
|  | GGACAGTGACGGTTGGATGTAC | GCAGACACTGGAAGAACTTTGC | 3469907 | 3470275 |
|  | CCCAAGTAGGGCTATGATGTGT | CCTAATAACACACTGTCTTTGTGTGTC | 3486516 | 3486868 |
|  | CCCTGTCTCAGGGTCTGAAAGA | GATTTAGTCGTGTTCCCTTGACAAG | 3494397 | 3494736 |
|  | GCCAAGTCCTGGAGCTCATTTC | CTGAGCTGAGAACCAGCAAAGCA | 3480253 | 3480619 |
|  | CGCCCTTTGGTTTTCTTAAAGAAGT | CTTCCAACCCGTTATTTCCTGTTC | 3493563 | 3493912 |
|  | CAGCATGAGTGAGGTGCTCAA | CGTGGGCATCATCAACGAAGA | 3474631 | 3474897 |
|  | GCTTTCTGCTCCCTCCTCAAATC | TTCAAGGCTGTCTTCATCATCCTG | 3476733 | 3477064 |
|  | CAAGGCCAGGGAGAATACCATG | TATACATAAAGAGTGAGATTTTGCACAGCT | 3480964 | 3481288 |
|  | CCCTTCTGGACACCTCGAATTT | GAGCTTCCTTAAGTGCATGAGGAA | 3475175 | 3475514 |
|  | CGTCATTCCCTTCTTGTTGGTGA | TTGGGCAGAGACAGAGGGAGTTT | 3493137 | 3493491 |
|  | GGCAGTAGACCAGGAAGTTGAAG | CACTTAAGAGCTTGTCCAGTTTCAC | 3489115 | 3489485 |
|  | GCAGGGTGAGGTTGATAACCAC | ACAAAATCCAAAATCTCGTATAAGCTCAGT | 3496006 | 3496315 |
|  | GCATCACTACCAGCATCACTACT | CAGGAAAGCTGTCCACAGTAGTC | 3511658 | 3512007 |
|  | GGGTTCCTAGAAATGGAAGATCTTTCAA | CGCTCGGCCTTCTTTGATTTTA | 3468821 | 3469192 |
|  | AAAAAGAATAAAATCAAAGAAAACAGCCAGG | TGGATGGTCAGTCTCTACTGGG | 3469479 | 3469852 |
| TRPV4 | CAGCAGGTCGTACATCTTGGTA | GCTGTCTCCGTCATTATCCTCAGA | 110236524 | 110236832 |
|  | ACTCTCCCTGGCCAGCATTATTA | TGTTCTGTAGTGTCTGGGATTTGC | 110220594 | 110220950 |
|  | TGAGAGCAGAGCAAATAAATAATGGAGAG | ATTTCTAGTCCAGCCGCATTTCA | 110221133 | 110221390 |
|  | GGATGATGAATGGGTGAATGGATACAG | CCCTGGCCAATCTGTTTGAGG | 110252151 | 110252498 |
|  | GTGAGCTGGACCACAAGGAGAA | TCATCATCCTGCTGGTGACCTA | 110225956 | 110226329 |
|  | TAGGCGGTGAGAGTGAAGATGA | TCATGAATCCGTGAATGCAGCT | 110232156 | 110232439 |
|  | CTCATCCTTGGGCTGGAAGAAG | GGGAATATCCAAGGACCTATTTGCTG | 110238442 | 110238729 |
|  | TCAGAGAGGTCACACATCGCTTA | CCATTCTTGCTGACCCACAAGA | 110245779 | 110246149 |
|  | AGAAATGGGAAAATAAAAGGAGGAAGGAA | GAAGACTCAGGAGGAAGAGAGTGA | 110231595 | 110231962 |
|  | GGGCACACTCTCTCATTCTGTA | CACGCCCAGCCTTGTTATGTATA | 110222000 | 110222363 |
|  | AGGAGAGGTCATAAAGCGAGGAA | GCCTCCATTCTTTATCTGTAAACCAAGATA | 110234395 | 110234688 |
|  | ACTTACCTCCGGGACGGCTG | TCGCGACACCCATTTTCCA | 110271149 | 110271443 |
|  | CTCCCTCCAGGAACACACGAGT | AAGGCATTGGGAAGTCCTGATAC | 110230411 | 110230735 |
|  | AAAGAACTCAGCAAGGCTGCTAT | GGGAAAGTGAGAAACCATGTGTC | 110230068 | 110230293 |
|  | TAAGGAGCTTGGCTGGACACTA | CTGACAACACCCGTGAGAACAC | 110236234 | 110236576 |
|  | TGAAGCGTAGCCGATCATGAAG | CCTATAGCATCATGATCCAGAAGGTAC | 110230169 | 110230479 |
|  | CAGTGGCTCCAGAAATTGCAATG | GAGTAGGAGTCTAAATGCCTAGTTCTG | 110224407 | 110224765 |
|  | CATCTCCACCGTCAATGAATAAACATTT | CTCCTATGGAGTCACATAAGCCAAC | 110220888 | 110221221 |
|  | CTGGAACTTCATGCGCAGATTT | ATTACAACGGTGGCTTTGAAGC | 110252386 | 110252751 |
|  | GGCACTTAGGCCCAGATCAAAA | GATGTTGACAGCCAGGACATCT | 110240698 | 110241061 |
|  | CTTCACTCTCTTCCTCCTGAGTCT | CCTTCTACATCAACGTGGTCTCCTA | 110231937 | 110232216 |
|  | GCTCACCTCGAAACTCCTCATC | ACTACCTCACCCTGTTCCTTGTA | 110246095 | 110246406 |
|  | CCCAAAAGTCCATCTTCCCACA | CAAGTTCAAGGACTGGGCCTAT | 110234111 | 110234450 |
|  | CATGTTGAGGAGCAGCACAAAG | TCCTCCAGCAAATGAGAATGACAC | 110226274 | 110226604 |
|  | TGGCTGGAGTGAGGATCTGAAT | AAACACTACGTGGAACTTCTCGT | 110238180 | 110238525 |
|  | TGTTTGTGGCTTTGTCCTGACT | TCTCCTTTTCTAACAGGAAAGAGTCCT | 110231179 | 110231552 |
|  | CTGAAATGCGGCTGGACTAGAA | CCCAGTACACAGTAGGCACTCAATATA | 110221367 | 110221666 |
| TSPO | CCTGATCAGCTGACTGGTTGATC | TGCAAAGGGCCAGGCTTATC | 43555098 | 43555471 |
|  | CTCCCAAATCCAGTGGGAGTT | GTGACGGCCACCACATCACA | 43558715 | 43559067 |
|  | CCGTCACGCTTTCATGACCA | AGGGCTGCTCTGTTCATCTC | 43559061 | 43559435 |
|  | TTCGGGAGGGAAAACTTGGACAAC | CCGATCTGGACTGTTCTCCACTA | 43547843 | 43548067 |
|  | GAGAGGTGGCTTTGAGGAGTG | CTGCCAAGAGTCGCAGCCAT | 43547407 | 43547678 |
|  | GTTTATTTTGCCCAAAGCCGGTTA | CATTCTGGGACAGCCTCAAGT | 43548072 | 43548410 |
|  | CAGTCAGTGTCAGGTCACGTAT | AGCTAGAGAGACATGGCCTTTTG | 43556953 | 43557327 |
|  | GACCACACTCAACTACTGCGTAT | CCAAGCAAAAAGGCCATGGTAA | 43558928 | 43559267 |
