# Supplementary material for: Development of an AmpliSeqTM Panel for Next-Generation Sequencing of a Set of Genetic Predictors of Persisting Pain
Source: Front Pharmacol. 2018 Sep 19;9:1008. doi: 10.3389/fphar.2018.01008 (PMC6156278; doi:10.3389/fphar.2018.01008)
Supplement: Supplementary file 2 [file Table_2.DOCX]

Supplementary Table 2: AmpliSeq™ amplicons and coverage details of the present NGS assay.

| Gene | Chr. | Num. of Amplicons | Total Bases | Covered Bases | Missed Bases | Coverage (%) |
| --- | --- | --- | --- | --- | --- | --- |
| ABHD12 | chr20 | 19 | 3111 | 3009 | 102 | 96,7 |
| ABHD16A | chr6 | 22 | 3531 | 3412 | 119 | 96,6 |
| ABHD6 | chr3 | 16 | 2843 | 2843 | 0 | 100 |
| ADCY1 | chr7 | 65 | 14031 | 13849 | 182 | 98,7 |
| ADRB2 | chr5 | 12 | 2092 | 2092 | 0 | 100 |
| BDNF | chr11 | 37 | 8279 | 8182 | 97 | 98,8 |
| CACNG2 | chr22 | 29 | 4723 | 4670 | 53 | 98,9 |
| CDK5 | chr7 | 13 | 1793 | 1793 | 0 | 100 |
| CHRNB2 | chr1 | 27 | 6018 | 5989 | 29 | 99,5 |
| CNR1 | chr6 | 24 | 6155 | 6155 | 0 | 100 |
| COMT | chr22 | 17 | 2953 | 2813 | 140 | 98,3 |
| CSF1 | chr1 | 32 | 5415 | 5184 | 231 | 95,7 |
| DLG4 | chr17 | 32 | 5964 | 5422 | 542 | 90,9 |
| DRD1 | chr5 | 14 | 3473 | 3473 | 0 | 100 |
| DRD2 | chr11 | 16 | 3099 | 2982 | 117 | 96,2 |
| DRD3 | chr3 | 11 | 2175 | 2175 | 0 | 100 |
| DRD4 | chr11 | 5 | 1562 | 656 | 906 | 42 |
| EGR1 | chr5 | 14 | 3236 | 3236 | 0 | 100 |
| ESR1 | chr6 | 32 | 7568 | 7568 | 0 | 100 |
| FAAH | chr1 | 21 | 2845 | 2845 | 0 | 100 |
| FKBP51 | chr6 | 47 | 11278 | 11068 | 210 | 98,1 |
| FOS | chr14 | 12 | 2358 | 2358 | 0 | 100 |
| FYN | chr6 | 23 | 4676 | 4346 | 330 | 92,9 |
| GABRA5 | chr15 | 19 | 3721 | 3710 | 11 | 99,7 |
| GALR2 | chr17 | 7 | 1457 | 1457 | 0 | 100 |
| GCH1 | chr14 | 15 | 3225 | 3225 | 0 | 100 |
| GDNF | chr5 | 26 | 4481 | 4430 | 51 | 98,9 |
| GFRA1 | chr10 | 56 | 10145 | 10141 | 4 | 99 |
| GPR132 | chr14 | 25 | 4120 | 3927 | 193 | 95,3 |
| GRIN1 | chr9 | 33 | 5495 | 4302 | 1193 | 78,3 |
| GRIN2A | chr16 | 63 | 15485 | 15485 | 0 | 400 |
| GRIN2B | chr12 | 27 | 6591 | 6591 | 0 | 100 |
| GRM5 | chr11 | 34 | 8523 | 8396 | 127 | 98,5 |
| HCN2 | chr19 | 17 | 3808 | 2592 | 1216 | 68,1 |
| HLA-DQB1 | chr6 | 10 | 1964 | 1762 | 202 | 89,7 |
| HLA-DRB1 | chr6 | 9 | 1516 | 1444 | 72 | 95,3 |
| HRH3 | chr20 | 12 | 2830 | 2331 | 499 | 82,4 |
| HTR1A | chr5 | 11 | 2295 | 2295 | 0 | 100 |
| HTR2A | chr13 | 23 | 5642 | 5642 | 0 | 100 |
| IL10 | chr1 | 9 | 1879 | 1503 | 376 | 80 |
| IL1B | chr2 | 10 | 1848 | 1848 | 0 | 100 |
| IL1R2 | chr2 | 13 | 2199 | 2199 | 0 | 100 |
| IL4 | chr5 | 4 | 818 | 818 | 0 | 100 |
| IL6 | chr7 | 10 | 1434 | 1434 | 0 | 100 |
| KCNS1 | chr20 | 20 | 4789 | 4789 | 0 | 100 |
| KIT | chr4 | 31 | 6226 | 6226 | 0 | 100 |
| LTB4R | chr14 | 21 | 4770 | 4770 | 0 | 100 |
| LTB4R2 | chr14 | 7 | 1660 | 1569 | 91 | 94,5 |
| NF1 | chr17 | 90 | 16160 | 16040 | 120 | 99,2 |
| NGF | chr1 | 6 | 1199 | 1199 | 0 | 100 |
| NTF4 | chr19 | 7 | 1099 | 1099 | 0 | 100 |
| NTRK1 | chr1 | 26 | 3807 | 3807 | 0 | 100 |
| OPRD1 | chr1 | 8 | 1924 | 1499 | 425 | 77,9 |
| OPRK1 | chr8 | 24 | 5446 | 5397 | 49 | 99,1 |
| OPRM1 | chr6 | 81 | 20323 | 19669 | 654 | 96,8 |
| OXT | chr20 | 4 | 663 | 652 | 11 | 98,3 |
| P2RX7 | chr12 | 25 | 4715 | 4709 | 6 | 99,9 |
| PLCB1 | chr20 | 48 | 8857 | 8767 | 90 | 99 |
| PRKCG | chr19 | 25 | 4028 | 4028 | 0 | 100 |
| PRNP | chr20 | 13 | 3064 | 3046 | 18 | 99,4 |
| PTN | chr7 | 10 | 1795 | 1795 | 0 | 100 |
| PTPRZ1 | chr7 | 48 | 9659 | 9638 | 21 | 99,8 |
| RELN | chr7 | 86 | 14821 | 14763 | 58 | 99,6 |
| RET | chr10 | 46 | 7397 | 7099 | 298 | 96 |
| RUNX1 | chr21 | 37 | 8481 | 8464 | 17 | 99,8 |
| S100B | chr21 | 7 | 1285 | 1285 | 0 | 100 |
| SCN9A | chr2 | 60 | 11110 | 10869 | 241 | 97,8 |
| SLC6A4 | chr17 | 34 | 7356 | 7305 | 51 | 99,2 |
| SOD2 | chr6 | 12 | 2075 | 2059 | 16 | 99,2 |
| TH | chr11 | 20 | 2610 | 2496 | 114 | 95,6 |
| TLR4 | chr9 | 23 | 5981 | 5981 | 0 | 100 |
| TNF | chr6 | 10 | 1875 | 1875 | 0 | 100 |
| TRPA1 | chr8 | 43 | 6540 | 6540 | 0 | 100 |
| TRPM8 | chr2 | 39 | 6921 | 6759 | 162 | 97,7 |
| TRPV1 | chr17 | 37 | 5901 | 5624 | 277 | 95,3 |
| TRPV4 | chr12 | 27 | 4039 | 3979 | 60 | 98,5 |
| TSPO | chr22 | 8 | 1419 | 1368 | 51 | 96,4 |
