# Supplementary material for: Development of an AmpliSeqTM Panel for Next-Generation Sequencing of a Set of Genetic Predictors of Persisting Pain
Source: Front Pharmacol. 2018 Sep 19;9:1008. doi: 10.3389/fphar.2018.01008 (PMC6156278; doi:10.3389/fphar.2018.01008)
Supplement: Supplementary file 3 [file Table_3.DOCX]

Supplementary Table 3: Genetic regions that are missing from the present NGS gene panel.

| Gene | Chromosome | Chr start | Chr end |
| --- | --- | --- | --- |
| OPRD1 | chr1 | 29138628 | 29139053 |
| CSF1 | chr1 | 110453491 | 110453709 |
| CSF1 | chr1 | 110471688 | 110471698 |
| CSF1 | chr1 | 110473468 | 110473471 |
| CHRNB2 | chr1 | 154540580 | 154540609 |
| IL10 | chr1 | 206941016 | 206941392 |
| SCN9A | chr2 | 167052258 | 167052260 |
| SCN9A | chr2 | 167052559 | 167052589 |
| SCN9A | chr2 | 167053087 | 167053233 |
| SCN9A | chr2 | 167053585 | 167053648 |
| TRPM8 | chr2 | 234873246 | 234873321 |
| TRPM8 | chr2 | 234927155 | 234927239 |
| TRPM8 | chr2 | 234927551 | 234927554 |
| GDNF | chr5 | 37813969 | 37814004 |
| GDNF | chr5 | 37839728 | 37839744 |
| ABHD16A | chr6 | 31655583 | 31655702 |
| HLA-DRB1 | chr6 | 32546834 | 32546906 |
| HLA-DQB1 | chr6 | 32632667 | 32632869 |
| FKBP51 | chr6 | 35548613 | 35548629 |
| FKBP51 | chr6 | 35549080 | 35549099 |
| FKBP51 | chr6 | 35549405 | 35549424 |
| FKBP51 | chr6 | 35656483 | 35656639 |
| FKBP51 | chr6 | 35656555 | 35656639 |
| FYN | chr6 | 112194145 | 112194475 |
| OPRM1 | chr6 | 154360888 | 154360994 |
| OPRM1 | chr6 | 154360888 | 154360994 |
| OPRM1 | chr6 | 154441725 | 154441818 |
| OPRM1 | chr6 | 154444841 | 154444856 |
| OPRM1 | chr6 | 154446906 | 154447279 |
| OPRM1 | chr6 | 154448250 | 154448274 |
| OPRM1 | chr6 | 154451261 | 154451281 |
| OPRM1 | chr6 | 154451594 | 154451617 |
| SOD2 | chr6 | 160113901 | 160113912 |
| SOD2 | chr6 | 160114201 | 160114206 |
| ADCY1 | chr7 | 45613784 | 45613795 |
| ADCY1 | chr7 | 45614125 | 45614265 |
| ADCY1 | chr7 | 45760126 | 45760157 |
| RELN | chr7 | 103124298 | 103124322 |
| RELN | chr7 | 103151272 | 103151306 |
| PTPRZ1 | chr7 | 121513321 | 121513342 |
| OPRK1 | chr8 | 54139856 | 54139883 |
| OPRK1 | chr8 | 54155379 | 54155381 |
| OPRK1 | chr8 | 54164176 | 54164196 |
| OPRK1 | chr8 | 54164176 | 54164196 |
| GRIN1 | chr9 | 140033589 | 140034028 |
| GRIN1 | chr9 | 140040152 | 140040191 |
| GRIN1 | chr9 | 140055758 | 140055893 |
| GRIN1 | chr9 | 140058015 | 140058046 |
| GRIN1 | chr9 | 140062331 | 140062880 |
| GRIN1 | chr9 | 140062331 | 140062880 |
| RET | chr10 | 43572491 | 43572785 |
| RET | chr10 | 43615188 | 43615192 |
| GFRA1 | chr10 | 118032914 | 118032918 |
| DRD4 | chr11 | 637457 | 637523 |
| DRD4 | chr11 | 639516 | 639570 |
| DRD4 | chr11 | 639622 | 640331 |
| DRD4 | chr11 | 640375 | 640443 |
| DRD4 | chr11 | 640703 | 640712 |
| TH | chr11 | 2185577 | 2185647 |
| TH | chr11 | 2187684 | 2187720 |
| TH | chr11 | 2189345 | 2189353 |
| BDNF | chr11 | 27722528 | 27722625 |
| BDNF | chr11 | 27722528 | 27722625 |
| GRM5 | chr11 | 88242570 | 88242697 |
| DRD2 | chr11 | 113281363 | 113281469 |
| DRD2 | chr11 | 113345795 | 113345806 |
| TRPV4 | chr12 | 110221367 | 110221388 |
| TRPV4 | chr12 | 110271129 | 110271168 |
| P2RX7 | chr12 | 121624373 | 121624379 |
| LTB4R2 | chr14 | 24780341 | 24780432 |
| LTB4R2 | chr14 | 24780341 | 24780432 |
| GPR132 | chr14 | 105516165 | 105516180 |
| GPR132 | chr14 | 105516815 | 105516894 |
| GPR132 | chr14 | 105517478 | 105517484 |
| GPR132 | chr14 | 105522063 | 105522068 |
| GPR132 | chr14 | 105524249 | 105524337 |
| GABRA5 | chr15 | 27112486 | 27112497 |
| TRPV1 | chr17 | 3469480 | 3469509 |
| TRPV1 | chr17 | 3495779 | 3496027 |
| DLG4 | chr17 | 7100132 | 7100160 |
| DLG4 | chr17 | 7106725 | 7106743 |
| DLG4 | chr17 | 7107082 | 7107134 |
| DLG4 | chr17 | 7120509 | 7120953 |
| SLC6A4 | chr17 | 28522248 | 28522268 |
| SLC6A4 | chr17 | 28522573 | 28522590 |
| SLC6A4 | chr17 | 28523135 | 28523149 |
| NF1 | chr17 | 29422376 | 29422390 |
| NF1 | chr17 | 29496883 | 29496933 |
| NF1 | chr17 | 29528411 | 29528467 |
| HCN2 | chr19 | 589867 | 590323 |
| HCN2 | chr19 | 590554 | 590584 |
| HCN2 | chr19 | 603518 | 603559 |
| HCN2 | chr19 | 613467 | 613513 |
| HCN2 | chr19 | 613826 | 613857 |
| HCN2 | chr19 | 615892 | 616473 |
| HCN2 | chr19 | 616582 | 616613 |
| OXT | chr20 | 3052833 | 3052844 |
| PRNP | chr20 | 4667389 | 4667407 |
| PLCB1 | chr20 | 8112947 | 8113037 |
| ABHD12 | chr20 | 25371217 | 25371314 |
| ABHD12 | chr20 | 25371584 | 25371589 |
| HRH3 | chr20 | 60790925 | 60790940 |
| HRH3 | chr20 | 60791382 | 60791386 |
| HRH3 | chr20 | 60791712 | 60791731 |
| HRH3 | chr20 | 60794887 | 60795348 |
| RUNX1 | chr21 | 36164651 | 36164668 |
| COMT | chr22 | 19929417 | 19929445 |
| COMT | chr22 | 19956806 | 19956918 |
| CACNG2 | chr22 | 36959232 | 36959244 |
| CACNG2 | chr22 | 36959583 | 36959599 |
| CACNG2 | chr22 | 36960129 | 36960154 |
| TSPO | chr22 | 43548044 | 43548095 |
| TSPO | chr22 | 43548044 | 43548095 |
| OPRD1 | chr1 | 29138628 | 29139053 |
| CSF1 | chr1 | 110453491 | 110453709 |
| CSF1 | chr1 | 110471688 | 110471698 |
| CSF1 | chr1 | 110473468 | 110473471 |
| CHRNB2 | chr1 | 154540580 | 154540609 |
| IL10 | chr1 | 206941016 | 206941392 |
| SCN9A | chr2 | 167052258 | 167052260 |
| SCN9A | chr2 | 167052559 | 167052589 |
| SCN9A | chr2 | 167053087 | 167053233 |
| SCN9A | chr2 | 167053585 | 167053648 |
| TRPM8 | chr2 | 234873246 | 234873321 |
| TRPM8 | chr2 | 234927155 | 234927239 |
| TRPM8 | chr2 | 234927551 | 234927554 |
| GDNF | chr5 | 37813969 | 37814004 |
| GDNF | chr5 | 37839728 | 37839744 |
| ABHD16A | chr6 | 31655583 | 31655702 |
| HLA-DRB1 | chr6 | 32546834 | 32546906 |
| HLA-DQB1 | chr6 | 32632667 | 32632869 |
| FKBP51 | chr6 | 35548613 | 35548629 |
| FKBP51 | chr6 | 35549080 | 35549099 |
| FKBP51 | chr6 | 35549405 | 35549424 |
| FKBP51 | chr6 | 35656483 | 35656639 |
| FKBP51 | chr6 | 35656555 | 35656639 |
| FYN | chr6 | 112194145 | 112194475 |
| OPRM1 | chr6 | 154360888 | 154360994 |
| OPRM1 | chr6 | 154360888 | 154360994 |
| OPRM1 | chr6 | 154441725 | 154441818 |
| OPRM1 | chr6 | 154444841 | 154444856 |
| OPRM1 | chr6 | 154446906 | 154447279 |
| OPRM1 | chr6 | 154448250 | 154448274 |
| OPRM1 | chr6 | 154451261 | 154451281 |
| OPRM1 | chr6 | 154451594 | 154451617 |
| SOD2 | chr6 | 160113901 | 160113912 |
| SOD2 | chr6 | 160114201 | 160114206 |
|  |  |  |  |
